# CoINcIDE: A framework for discovery of patient subtypes across multiple datasets

**DOI:** 10.1186/s13073-016-0281-4

**Published:** 2016-03-09

**Authors:** Catherine R. Planey, Olivier Gevaert

**Affiliations:** The Stanford Center for Biomedical Informatics Research (BMIR), Department of Medicine, Stanford University, 1265 Welch Road, Stanford, CA 94305 USA

## Abstract

Patient disease subtypes have the potential to transform personalized medicine. However, many patient subtypes derived from unsupervised clustering analyses on high-dimensional datasets are not replicable across multiple datasets, limiting their clinical utility. We present CoINcIDE, a novel methodological framework for the discovery of patient subtypes across multiple datasets that requires no between-dataset transformations. We also present a high-quality database collection, curatedBreastData, with over 2,500 breast cancer gene expression samples. We use CoINcIDE to discover novel breast and ovarian cancer subtypes with prognostic significance and novel hypothesized ovarian therapeutic targets across multiple datasets. CoINcIDE and curatedBreastData are available as R packages.

**Electronic supplementary material:**

The online version of this article (doi:10.1186/s13073-016-0281-4) contains supplementary material, which is available to authorized users.

## Background

Subtyping patient disease populations using high-dimensional molecular data and unsupervised clustering algorithms has transformed how researchers and clinicians interpret and quantify heterogeneity within a disease. Early gene expression cancer subtyping research showed that gene expression patterns can stratify patients into subtypes with distinct survival patterns [1]; such subtypes have the potential to drive personalized patient treatment regimens [2] and risk prediction models [3]. Unfortunately, while many studies report novel subtypes for various diseases [2, 4, 5], these subtypes are rarely routinely implemented in clinical practice. A major hurdle is that subtypes derived from high-dimensional data platforms are oftentimes not replicable [6]. In a recent opinion article discussing trustworthy experiments, replicability is defined as ‘the chance that an independent experiment targeting the same scientific question will produce a consistent result’ [7]. In the case of patient subtypes, this means subtypes with similar signaling patterns can be found across multiple datasets. Replicability is a key aspect of defining whether an analysis is trustworthy or not; if a clinician cannot trust the analysis that produced the subtypes, then there is little hope for widespread adoption and a true translation from bench to bedside.

There are two large hurdles to producing replicable patient subtypes: a lack of curated disease-specific dataset collections and a lack of methods that discover consensus across clusterings from multiple datasets. When a collection of several datasets is available, there exist few widely adopted approaches to clustering multiple datasets to derive patient subtypes. Arguably the most popular approach is to concatenate all of the datasets into a single data matrix and then cluster this matrix. While this method can find interpretable signal [8], a large drawback is that the datasets must first be transformed using batch correction techniques to remove dataset-specific noise [8, 9]. While these methods smooth out signal variances across datasets that may indeed be noise artifacts, these variances might also be true signaling patterns. An example of the latter case is when datasets are from different targeted clinical trials with different latent disease subtypes. An additional drawback to concatenation is that it provides a single clustering, and thus valuable information about the consensus of the datasets within each subtype is lost. In supervised replicability analyses, it is common to not concatenate data matrices, but to confirm consensus of signaling patterns within, and then across, each dataset by conducting a meta-analysis with metrics such as effect size [10]. Here, an effect size for each feature (gene) is first computed within each dataset using binary supervised labels, and then a summary effect size across all datasets is computed; features with a large summary effect size are interpreted as being robust to dataset-specific noise artifacts and more likely to represent signal that highly distinguishes patients that do not share the same binary label. This meta-analysis approach has shown promise in high-dimensional molecular datasets across various platforms without any batch correction transformations to discover repeatable signal in supervised analyses [11].

However, an analogous meta-analysis method does not exist in the unsupervised realm for patient subtypes; consensus clustering and ensemble clustering evaluate cluster stability within a single dataset [12, 13] and methods to discover repeatable feature, for example, gene, subtypes, oftentimes rely upon the fact that feature labels are shared across datasets [14], which is not the case for patient labels across different institutions or clinical trials. There have been efforts to cluster across clinical datasets, but these methods assume the starting point of the analysis is an existing set of pre-defined edges (relationships) between nodes, which is normally not the case for *de novo* clustering analyses [15]. We propose a novel methodological framework called CoINcIDE that discovers robust patient subtypes, or meta-clusters, across multiple datasets. CoINcIDE builds upon the In-Group-Proportion metric [16], a method that quantifies the replicability of a single set of subtypes applied to a single external dataset. CoINcIDE does not require batch correction techniques, as it expands upon the meta-analysis approach to discover consensus across individual clusterings from each dataset, as opposed to a single concatenated matrix.

**Fig. 1:**
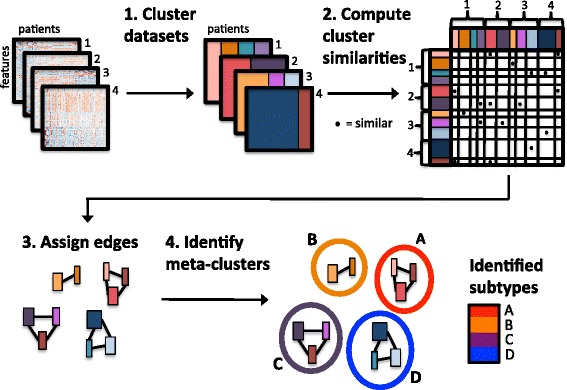
CoINcIDE steps. The four main steps to CoINcIDE, a method to discover replicable patient subtypes by finding consensus across dataset-specific clusterings from multiple datasets. The first step of CoINcIDE is to derive these dataset-specific clusterings. The second step is to compute cluster-cluster similarities between each cluster-cluster pair, resulting in an adjacency matrix (clusters within the same dataset are not compared). The third step is to assign an edge between cluster pairs whose similarity passes set magnitude and significance thresholds, with the weight of the edge equaling the similarity magnitude. This creates a network. The fourth step is to then discover meta-clusters via network community detection methods. The resulting meta-clusters are the final subtypes

Here we present a comprehensive framework called CoINcIDE: Clustering Intra and Inter DatasEts (Fig. 1). CoINcIDE enables researchers to discover truly replicable subtypes and is implemented as an R package providing functionality from initial data processing to final meta-cluster functional analyses. Next, we present a high-quality database collection of 24 breast cancer gene expression datasets encompassing 15 studies with linked outcomes and treatment information as a second R package. We apply CoINcIDE on this breast cancer collection and a previously developed ovarian cancer dataset collection [17]. We show that CoINcIDE validates known breast cancer subtypes and discovers ovarian cancer subtypes with prognostic significance and novel hypothesized therapeutic targets, all across multiple datasets.

## Methods

We have developed a comprehensive suite of resources, tools, and methods to enable researchers to conduct robust meta-cluster analyses and discover replicable patient subtypes in the form of two R packages: CoINcIDE, which implements a set of novel methods for the discovery of patient subtypes across multiple datasets and curatedBreastData, a breast cancer dataset collection providing 24 high-quality curated breast cancer microarray datasets.

### Breast cancer collection: curatedBreastData

We used 17 gene expression microarray datasets from the curatedBreastData Bioconductor package; this package includes primary human tissue breast cancer datasets collected from the National Center for Biotechnology (NCBI) Gene Expression Omnibus (GEO) [18]. Datasets with sample sizes over 30 and a minimum 35-gene set from the full PAM50 gene set [19] (Additional file 1: Table S1) were used for analyses. Datasets with sample sizes under 30 did not comprise unique studies, but rather were small sets of microarrays from larger studies (also included in curatedBreastData) that had been run on different platforms. These filters resulted in 2,235 pre-treatment patient tumor sample microarrays encompassing 17 datasets and 15 studies across six different platforms. See Table 1 for dataset names and platform details; sample sizes ranged from 46 to 286 and the average number of genes per platform was approximately 11,000.

**Table 1:** Characteristics of curated breast cancer datasets

Dataset	ID	Batch ID	GEO platform ID	Commercial platform name	Samples (n)	Genes (n)
1	12093		GPL96	Affymetrix Human Genome U133A	136	11,723
2	1379			Arcturus 22 k human oligonucleotide	60	11,723
3	16391			Affymetrix Human Genome U133 Plus 2.0	48	15,199
4	16446			Affymetrix Human Genome U133A	114	16,326
5	17705	JBI		Affymetrix Human Genome U133A	103	10,565
6	17705	MDACC		Affymetrix Human Genome U133A	195	11,026
7	19615			Affymetrix Human Genome U133 Plus 2.0	115	16,652
8	20181			Affymetrix Human Genome U133A	53	10,171
9	20194			Affymetrix Human Genome U133A	261	11,748
10	2034			Affymetrix Human Genome U133A	286	11,020
11	22226			Agilent-012391 Whole Human Genome Oligo G4112A	127	18,841
12	22358			AFFY Human Phase3 v1.0 - C02	121	17,253
13	25055	MDACC_M		Affymetrix Human Genome U133A	221	11,459
14	25065	MDACC		Affymetrix Human Genome U133A	71	11,158
15	25065	USO		Affymetrix Human Genome U133A	54	10,822
16	32646			Affymetrix Human Genome U133 Plus 2.0	115	18,260
17	9893			MLRG Human 21 K V12.0	155	13,154

All breast cancer datasets are from the Gene Expression Omnibus (GEO). ‘Batch dataset’ does not refer to a lab replicate batch, but rather a group of microarrays from a larger study that were run on a different platform, or collected from a different site. These batch labels were inferred from GEO sample file names. GSE = GEO series ID prefix

### Ovarian cancer collection: curatedOvarianData

We used 24 gene expression microarray datasets from the curatedOvarianData Bioconductor package [17]. These datasets contain primary tissue samples from pre-treatment ovarian tumors with various histological types. Datasets were used that had over 80 % of genes from the meta-rank gene sets as described in later sections. This filter resulted in 3,105 samples, 10 microarray platforms, and 24 datasets with two of the datasets derived from the same GEO study. Table 2 gives details for each dataset; sample sizes ranged from 28 to 578 and the average number of genes per platform was approximately 15,000.

**Table 2:** Characteristics of curated ovarian cancer datasets

Dataset	ID	Batch ID	GEO platform ID	Commercial platform name	Samples (n)	Genes (n)
1	E.MTAB.386		GPL6104	Illumina HumanRef-8 V2	129	10,560
2	GSE12470		GPL887	Agilent G4110b	53	18,940
3	GSE13876		GPL15718	Operon Human v3	157	20,822
4	GSE14764		GPL96	Affy U133a	80	13,769
5	GSE17260		GPL6480	Agilent G4112f	110	20,867
6	GSE18520		GPL570	Affy U133 Plus 2.0	63	20,440
7	GSE19829	GPL570	GPL570	Affy U133 Plus 2.0	28	20,440
8	GSE19829	GPL8300	GPL8300	Affy U95 v2	42	9,674
9	GSE20565		GPL570	Affy U133 Plus 2.0	140	20,440
10	GSE2109		GPL570	Affy U133 Plus 2.0	204	20,440
11	GSE26193		GPL570	Affy U133 Plus 2.0	107	20,440
12	GSE26712		GPL96	Affy U133a	195	13,769
13	GSE30161		GPL570	Affy U133 Plus 2.0	58	20,440
14	GSE32062		GPL6480	Agilent G4112f	260	20,867
15	GSE32063		GPL6480	Agilent G4112f	40	20,867
16	GSE44104		GPL570	Affy U133 Plus 2.0	60	20,440
17	GSE49997		GPL2986	ABI Human Genome Survey V2	204	16,048
18	GSE6008		GPL96	Affy U133a	172	8,744
19	GSE6822		GPL80	Affy Hu6800	66	5,251
20	GSE9891		GPL570	Affy U133 Plus 2.0	285	20,440
21	PMID15897565		GPL96	Affy U133a	63	13,769
22	PMID17290060		GPL96	Affy U133a	117	13,769
23	PMID19318476		GPL96	Affy U133a	42	13,769
24	TCGA		GPL3291	Affy HT U133a	578	13,769

Datasets were collected from several repositories, including GEO. GSE = GEO series ID prefix; this prefix is only included for datasets taken directly from GEO

### CoINcIDE

CoINcIDE encompasses four steps to discover and visualize replicable patient subtypes by finding consensus across dataset-specific clusterings from multiple datasets. The clusters are represented as nodes in a network, and the final subtypes are meta-clusters of tightly connected clusters within this network. The four main steps of CoINcIDE are outlined in Fig. 1.

#### Step 1: Select features and cluster each individual dataset

The input to our proposed method is a collection of datasets. Each matrix has genes or features in the rows and patients or samples in the columns. Based on extensive analysis (Additional file 1: Table S2; Additional file 2: Supplemental Methods), for microarray gene expression applications, we suggest consensus clustering [12] implemented with Hartigan Wong’s k-means algorithm [20] using one random start and 90 % resampling of samples along with a rounded Proportion of Ambiguous Clusters (PAC) score [21] as the optimal single-dataset clustering method (see Additional file 1: Table S2, Additional file 2: Supplemental Methods, and Additional file 3: Figures S1 and S2 for details).

Each individual dataset is clustered using a gene set that represents a union of the genes present across all microarrays. The user can decide the minimum number of genes that have to overlap between different datasets with suggested thresholds ranging from 70 % to 100 %. If the user does not have a pre-defined gene set, CoINcIDE also includes a meta-ranking gene selection method (see Additional file 2: Supplemental Methods for details).

#### Step 2: Compute similarities between clusters from different datasets

The CoINcIDE cluster-cluster metrics and significance test build upon the In-Group-Proportion (IGP) [16]. After each dataset is clustered in CoINcIDE’s step 1, clusters from dataset 1 and clusters from dataset 2 are compared as follows: centroids are derived from the clusters within dataset 1. Then, each cluster from dataset 2 is compared to these centroids. Patients from dataset 2, cluster A (‘2-A’) are each assigned using Pearson’s correlation to their respective nearest centroid in dataset 1. The centroid for which the highest fraction of patients from cluster ‘2-A’ are assigned is deemed the best cluster fit for cluster ‘2-A’; let us assume in this case cluster ‘1-C’ is the best fit. Two cluster-cluster metrics are then calculated: a nearest neighbor fraction, which is the number of patients in cluster ‘2-A’ assigned to cluster ‘1-C’ divided by the total number of patients in cluster ‘2-A’, and a similarity metric, which is the overall mean of Pearson’s correlation matrix between cluster centroid ‘1-C’ and all patients in cluster ‘2-A’. This is done for each cluster in dataset 2.

For the optimal ‘2-A’-‘1-C’ cluster-centroid set match, a *P* value is computed by generating null centroid sets based on the centroid sets in dataset 1 using the IGP null distribution methods [16]; the final *P* value is the number of times the real patients from ‘2-A’ were assigned to a null centroid set that resulted in a mean similarity metric and a nearest neighbor fraction greater than the true ‘2-A’-‘1-C’ values. We suggest 500 null centroid iterations to compute this *P* value. CoINcIDE uses the third null centroid set method proposed by Kapp and Tibshirani; this is the same method used in the authors’ R package, clusterRepro [16]. Finally, the roles of datasets 1 and 2 are reversed so that the centroids are derived from dataset 2 and the clusters in dataset 1 are treated as individual datasets. Cluster ‘2-A’ and cluster ‘1-C’ are only considered to be potentially similar clusters if they are identified as the most similar cluster-cluster pair in both iterations with datasets 1 and 2. Assuming this is the case, the nearest neighbor fraction and similarity metric are averaged, and only these cluster-cluster pairs are evaluated in step 3.

#### Step 3: Derive cluster network by assigning edges to similar clusters

Cluster-cluster pairs that pass user-defined nearest neighbor fraction, mean similarity metric and *P* value thresholds are assigned an edge. The weight of this edge is the mean similarity metric. We recommend an averaged nearest neighbor fraction of 0.7 and a *P* value threshold of 0.01 (applied to both *P* values from the cluster-cluster pair) to produce highly significant clusters with strongly homogenous patient make-up. A user can test various mean similarity thresholds, which will produce slightly different CoINcIDE meta-clusters. Because CoINcIDE takes into the account the similarity value of each edge (see Step 4), a few edges with low mean similarity weights will not highly alter the final meta-cluster patient assignments. On the other hand, aggressively pruning edges by using a similarity threshold near the maximum possible value of one will certainly remove clusters, and perhaps entire datasets, that contain reliable signaling patterns, albeit with some added noise.

We recommend an unbiased method to select the minimum mean similarity threshold, which involves inspecting the frequencies of mean similarity values across all datasets between all clusters (including mean similarities between non-optimal clusters.) We fit these frequencies to a Gaussian density curve for visualization and inspection; we recommend using a threshold value close to the similarity metric value that occurs at a local maximum or peak that is greater than a 0.1 baseline threshold.

For the two breast cancer PAM50 analyses we used similarity thresholds of 0.15 and 0.25 based on these density curves (Additional file 3: Figure S3A-B). The final meta-cluster patient assignments are not highly affected by small changes in the similarity threshold; running the first breast cancer analysis also using a threshold of 0.25 produced the same results. For the 50, 264, and 2020-gene set non-PAM50 analyses, similarity thresholds of 0.4, 0.5, and 0.5 were chosen, respectively, as these values represented a local maxima in the density curve for these experiments (figure not shown.) Using the density curve method again, we chose a threshold of 0.5 for both ovarian analyses (Additional file 3: Figure S3C-D). In the second ovarian analysis’s density curve, there are two local maxima above the recommended 0.1 threshold, one at 0.5 and one at 0.7 (Additional file 3: Figure S3D). We thus ran two separate CoINcIDE analyses using each maxima as a threshold to better understand how the resulting networks differ.

#### Step 4: Identify meta-clusters using network community detection methods

The Girvan-Newman community detection algorithm is then used to identify meta-clusters. This algorithm takes as input the edge and edge weight matrices derived from a sample-sample adjacency matrix, and outputs the final discovered meta-clusters [22] (Fig. 1). CoINcIDE implements the Girvan-Newman algorithm using the R package igraph [23]. To ensure meta-clusters of a reasonable size, meta-clusters with clusters from less than three unique datasets are removed from the final network.

### CoINcIDE evaluation with simulated dataset clusters

Seven different sets of clustered datasets were simulated from a real gene expression dataset containing four tissue types using Eigen decomposition methods (see Additional file 2: Supplemental Methods for details.) Each set contained 10 datasets constructed in a similar manner. Unless noted, a cluster was defined to contain all patient samples of the same tissue type. All clusters contained the logged expression of 200 simulated genes (see Additional file 3: Figure S4 for example simulated expression heatmaps.) The first set contained datasets with evenly sized clusters of 50 patient samples each for all four possible tissue types (Additional file 3: Figure S4B). The second set contained clusters derived in a similar manner to set one, but each cluster was assigned a random size ranging from 1 to 100 samples; the third set extended the methods of the second set by allowing a random number of clusters per dataset ranging from 2 to 4 clusters, and the fourth set allowed the number of clusters to randomly range between 1 and 4. The fifth and sixth sets combined the different cluster sizes from set three with the number of clusters in each dataset ranging from 2 to 4 and then 1 to 4, respectively. In the seventh and final set, two of the clusters from set 1 were replaced by clusters with a random mixture of tissue types (Additional file 3: Figure S4C).

For each of these seven sets, random normal noise with a mean of zero and increasing standard deviation from 0 to 2.4 was added to each of the 10 datasets. Each set was simulated 50 times, and true positive rates (TPR) and false positive rates (FPR) from the CoINcIDE meta-clusters were computed and averaged across all 50 iterations. A true positive was defined as an edge being connected between two clusters of the same tissue type. Finally, several minimum mean similarity thresholds ranging from 0.0 to 1.0 were tested. The minimum nearest neighbor fraction was held at 0.7, the individual *P* value maximum threshold was held at 0.01 and subtypes with less than three unique datasets were removed to match the thresholds used in all CoINcIDE analyses with real data.

### CoINcIDE evaluation with breast cancer PAM50 centroid clusters

We first implemented CoINcIDE under a highly controlled, semi-supervised clustering scenario to ensure that CoINcIDE can re-discover known signal. We used the PAM50 centroid sets that define five breast cancer subtypes using 50 genes [24] to derive highly distinct, clear clusters; these are well-established centroid sets [3, 25, 26]. The original PAM50 centroids for each of the five breast cancer subtypes (Normal, Basal, Luminal A, Luminal B, and HER2) were downloaded online from the UNC Microarray Database [27]. HUGO gene symbols were updated to their latest version using the HGNChelper R package [28]. No dataset had less than 35/50 of these genes; this minimum 35-gene set contained the key hormonal signaling genes ESR1 and ERBB2 (Additional file 1: Table S1). Each group of patients assigned to a specific PAM50 subtype (centroid) within a dataset was defined as a cluster (the maximum number of PAM50 genes found in each dataset was used to make these assignments.) Pearson’s correlation was used to assign a patient to the optimal PAM50 subtype, as this is the similarity metric used by the commercial PAM50 platform algorithm [29].

### CoINcIDE application with *de novo* PAM50 gene set clusters

The PAM50 feature set was used again, allowing the maximum number of PAM50 genes found in a specific dataset to cluster that individual dataset. Each dataset was now *de novo* clustered using the suggested methods from CoINcIDE Step 1.

### Comparison of CoINcIDE with concatenated matrix clustering

CoINcIDE was compared with the method of concatenating datasets and then clustering them to compare their abilities to discover replicable and biologically intuitive subtypes. Because concatenation requires that all features are found across all datasets, CoINcIDE was re-run using only the minimum 35-gene PAM50 gene set found across all 17 datasets (Additional file 1: Table S1) for a fair evaluation; a similarity threshold of 0.3 was implemented based on the density plot (figure not shown).

All 17 breast cancer datasets were included in the concatenated data matrix. Concatenated matrices were tested with three transformation methods: no transformation, gene-wise Batch Mean Centering (BMC) and ComBat [9]; each transformation was run before clustering the concatenated matrix (see Additional file 2: Supplemental Methods for details). The effects of the BMC and ComBat transformations on the actual PAM50 centroid classifications were also investigated by using the PAM50 centroid classification of each patient as the actual subtype, before any transformations, after BMC, and alternatively after ComBat. A supervised analysis using the baseline non-transformed datasets was also run using the full PAM50 gene set, to ensure that using the smaller intersecting PAM50 gene set on the BMC and ComBat supervised analyses did not significantly alter AUC values. Survival analyses were conducted for all breast cancer analyses (see Additional file 2: Supplemental Methods for details). Finally, to inspect the effect of BMC on CoINcIDE, BMC was applied to each individual dataset using the maximum number of PAM50 genes found in each dataset and then CoINcIDE was run, using the same parameters as the initial *de novo* PAM50 CoINcIDE analysis. The effect of ComBat on CoINcIDE was not inspected because ComBat cannot be run on an individual dataset.

### CoINcIDE application with breast cancer meta-rank gene *de novo* clusters

CoINcIDE was then run using the same k-means clustering scheme as in the above methods section with three meta-ranked gene lists to test CoINcIDE on differing gene set sizes that contained none of the PAM50 genes included in the earlier clustering gene sets. Gene sets of 50, 264, and 2,020 genes were selected via the meta-ranking algorithm (PAM50 genes were removed before the meta-ranking algorithm was run.) The 50-gene set test was included because the PAM50 gene set includes 50 genes. This gene set could not include the top 20 dataset-specific genes for each dataset, as this resulted in more than 50 genes (Additional file 1: Table S3; see Additional file 2: Supplemental Methods for details on the meta-rank algorithm), but the latter two feature sets did include these additional top-ranked genes by dataset. Initial global meta-rank sets of 200 and 2,000, respectively, were chosen, and then any non-intersecting genes that were ranked in the top 20 genes by mean absolute difference for a specific dataset were included. This resulted in two features sets with 264 and 2,020 genes (Additional file 1: Tables S4 and S5, respectively.)

### CoINcIDE application with ovarian cancer meta-rank gene *de novo* clusters

Finally, we tested CoINcIDE using the same k-means clustering scheme as in the above methods section with two meta-ranked gene lists to test CoINcIDE differing gene set sizes. The two gene sets were chosen in exactly the same manner as the breast cancer 200 and 2,000 initial global-rank sets with additional dataset-specific intra-ranked genes. This resulted in two features sets with 240 and 2,014 genes (Additional file 1: Tables S6 and S7, respectively.)

## Results

### CoINcIDE: A framework for subtype discovery across multiple datasets

CoINcIDE is a novel framework that identifies patient subtypes, or meta-clusters, from clustering individual datasets and then assigning edges between similar clusters across datasets to create a network and identify subtypes through community detection (Fig. 1) [22]. The resulting subtype network provides a robust platform for evaluating the effect sizes of genes within each subtype across multiple datasets and an intuitive visualization technique to better understand cluster-cluster and dataset-dataset interactions. To demonstrate our work to cluster across multiple datasets and discover replicable subtypes, we first use simulated datasets, and then we use two collections of cancer data: a breast cancer collection and an ovarian cancer collection. The breast cancer collection constitutes 2,719 patients from 24 breast cancer gene expression studies encompassing 34 datasets (Table 1) [30]. The ovarian cancer collection contains 3,105 patients from 24 gene expression microarray datasets (Table 2) [17]. We first validate CoINcIDE using simulated data to illustrate the performance of CoINcIDE in a controlled environment. Subsequently we apply CoINcIDE on these two cancer dataset collections to showcase its abilities on a disease with well-known subtypes, that is, breast cancer, and one without established subtypes, ovarian cancer.

### CoINcIDE re-discovers ground truth subtypes *in silico* and in a breast cancer collection

**Fig. 2:**
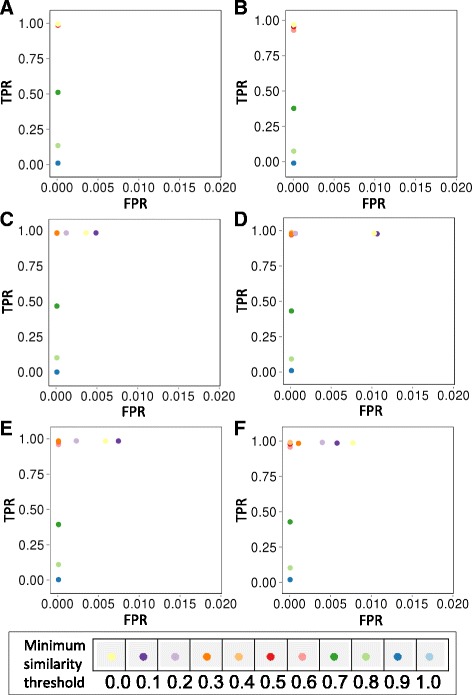
True positive rates (TPR) and true negative rates (TNR) of simulations. TPR plotted against FPR for the first six CoINcIDE logged gene expression simulation scenarios for varied minimum mean similarity thresholds. The FPR is truncated at 0.02 because no FPR values ever reached above this threshold. **a**-**f** were produced using the set standard deviation random normal noise level of 0.8 (Additional file 3: Figure S5A-G plot in detail each simulation scenario at more noise levels.) **a** The results for the high quality simulation scenario with an equal cluster size and equal number of clusters, (**b**) results for the random cluster size and equal number of clusters, (**c**) results for the equal cluster size and random number of clusters with a minimum of two clusters per dataset, (**d**) results for the equal cluster size and random number of clusters with a minimum of one cluster per dataset, (**e**) results for the random cluster size and random number of clusters per dataset with a minimum of two clusters per dataset, and (**f**) results for the random cluster size and random number of clusters per dataset with a minimum of one cluster per dataset scenario. The seventh simulation is not shown here due to space constraints but plots for this simulation scenario for equal cluster size but 50 % random/noisy clusters can be found in Additional file 3: Figures S5G and S6G

We used a gene expression dataset containing four different lung tissue types to simulate highly distinct patient clusters using different scenarios: equal cluster sizes, mixed cluster sizes, and then mixes of both number of cluster sizes and numbers of clusters per dataset (see Methods and Additional file 3: Figure S4). Across seven simulation scenarios for varying levels of noise and mean similarity metric thresholds, CoINcIDE re-discovered the tissue-specific subtypes with high TPRs and low FPRs (Fig. 2). A minimum similarity threshold of 0.3 maintained high TPRs and zero FPRs for all simulations; only when this threshold was lowered to 0.0 did we observe FPRs over 1.0 % (Fig. 2, Additional file 3: Figure S5A-G). The TPR for all seven simulation scenarios with increasing noise levels decreased as the minimum mean similarity threshold was increased (Fig. 2, Additional file 3: Figure S6A-G). The final simulation, serving as the negative control, had a lower TPR rate for all similarity thresholds, as noisy clusters were never assigned edges to any other clusters (Additional file 3: Figure S5G and Figure S6G). Simulations with a random number of clusters that allowed datasets to have only one cluster tended to have a slightly lower TPR for all noise levels (Additional file 3: Figure S6D and F).

**Fig. 3:**
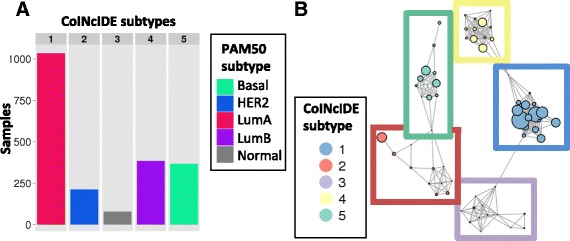
Breast PAM50 supervised centroid clustering results. **a** Bar plots summarizing patients in the CoINcIDE PAM50 centroid clustering meta-clusters or subtypes by their PAM50 classification. **b** Resulting cluster-cluster network for PAM50 centroid clustering across 16 datasets; one dataset did not have clusters that met the *P* value, nearest neighbor fraction (NNF) and mean similarity thresholds (0.01, 0.7, 0.15, respectively.) Node size is scaled to the relative number of samples in each cluster

Next, for our first breast cancer analysis, we used the supervised PAM50 centroid classifier that defines five breast cancer subtypes using 50 genes [24] to derive highly distinct, clear clusters. We defined each group of patients assigned to a specific PAM50 subtype within a dataset as a cluster. CoINcIDE correctly re-discovered all five PAM50 subtypes, with all patients in the network assigned to their true PAM50 subtype (Fig. 3a). These CoINcIDE meta-clusters were not affected by small variations in the similarity threshold level used; for example, while the threshold used was 0.15 for this CoINcIDE analysis based off of the similarity density curve (Additional file 3: Figure S3A), applying a threshold of 0.3 to both this PAM50 feature set breast cancer analysis and the subsequent *de novo* PAM50 clustering analysis described below did not change any of the final meta-cluster results.

The corresponding PAM50 centroid CoINcIDE network shows the relationships between the five meta-clusters (Fig. 3b, Additional file 3: Figure S7); except for one cluster, the Basal meta-cluster which is highly separated from all other meta-clusters. Clusters from one dataset (dataset 17 in Table 1) had no edges that passed the CoINcIDE thresholds for the PAM50 centroid classifier analysis, and thus were not present in the final network (Additional file 3: Figure S7). This same pattern was observed for all of the breast *de novo* clustering analyses described in later sections. Clusters from the three two-channel microarray datasets (datasets 1, 12, and 13 in Additional file 3: Figure S7) tended to be more weakly connected than clusters from the other datasets, which were all one-channel microarrays (Table 1).

**Fig. 4:**
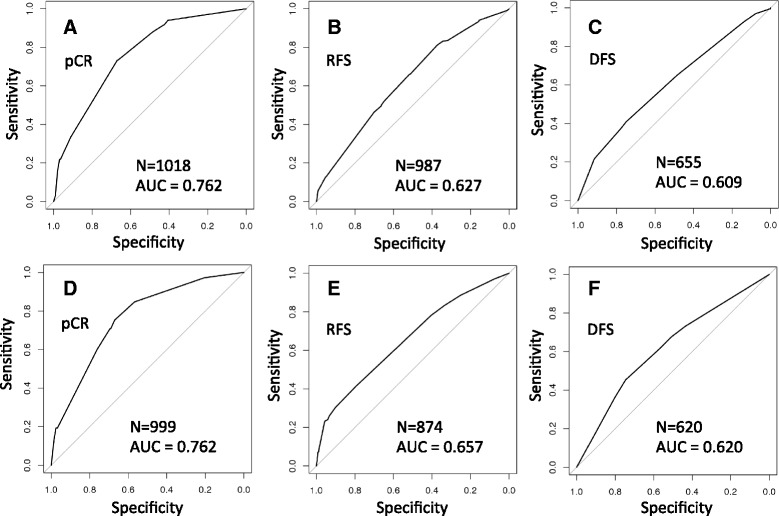
CoINcIDE breast PAM50 analyses ROC curves. ROC curves and AUC calculations for the breast PAM50 centroid clustering (**a-c**) and the PAM50 *de novo* clustering (**d-f**) CoINcIDE analyses. Each patient’s meta-cluster status and treatment information was included in a logistic regression model; the binary response variable used is printed under each figure letter. N = number of patients used in model

In logistic regression models combining treatment status variables and PAM50 centroid CoINcIDE meta-cluster assignments for each patient, the AUCs for predicting binary pathological complete response (pCR), relapse-free survival (RFS), and disease-free survival (DFS) were 0.762, 0.627, and 0.609, respectively (Additional file 1: Table S8, Fig. 4a-c). Adding the meta-cluster assignments in addition to baseline treatment status models was significant (*P* value <2.2E-16, 2.24E-05, and 1.51E-03; see Additional file 1: Table S8 and Additional file 2: Supplementary Methods for details).

### CoINcIDE identifies known breast cancer subtypes using *de novo* clusterings from 17 datasets

**Fig. 5:**
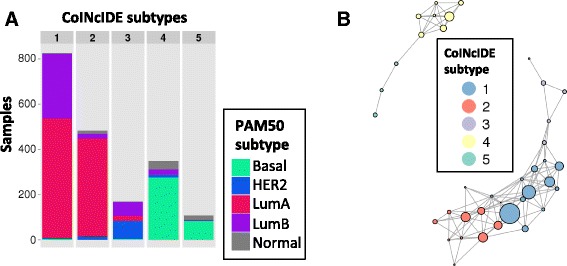
CoINcIDE PAM50 gene set *de novo* clustering analysis. **a** Bar plots summarizing patients in the CoINcIDE PAM50 *de novo* clustering meta-clusters or subtypes by their PAM50 classification. **b** Resulting cluster-cluster network for PAM50 *de novo* clustering across 16 datasets; one dataset did not have clusters that met the *P* value, nearest neighbor fraction, and mean similarity thresholds (0.01, 0.7, 0.25, respectively.) Node size is scaled to the relative number of samples in each cluster

Next, we used CoINcIDE to find *de novo* clusters in the breast cancer cohort using the PAM50 gene set. CoINcIDE discovered five subtypes (Fig. 5a); one was a Luminal A/Luminal B mixture, one was predominantly Luminal A, one was a HER2/Luminal B mixture, and two were predominantly Basal. The second Basal meta-cluster contained only three datasets (Additional file 3: Figure S8). The AUCs for treatment status plus meta-cluster assignment were 0.762, 0.657, and 0.620 for predicting pCR, RFS, and DFS, respectively (Fig. 4d-f); the corresponding *P* values for adding meta-cluster assignments to baseline treatment status were <2.2E-16, 4.40E-09, and 1.04E-03 (Additional file 1: Table S8). In the final network, several edges crossed between the Luminal A and Luminal A/B meta-clusters (Fig. 5b).

**Fig. 6:**
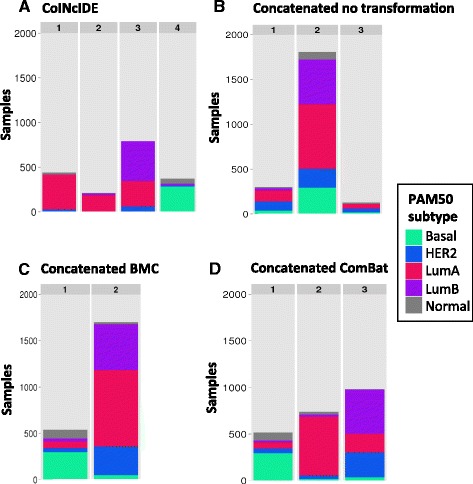
CoINcIDE versus concatenated results. Bar plots summarizing the counts for each PAM50 centroid subtype for each subtyping result from (**a**) PAM50 restricted 35-gene set CoINcIDE k-mean clustering, (**b**) concatenated matrix with no transformation, (**c**) concatenated matrix with gene-wise batch mean centering (BMC), and (**d**) concatenated matrix with ComBat. All analyses used k-means consensus clustering using the Proportion of Ambiguous Clusters (PAC) to select the number of clusters. These bar plots used only the 35-gene set to assign patients to PAM50 centroid subtypes (Additional file 1: Table S1). PAM50 centroid assignments were made on the pre-transformed concatenated datasets to allow for direct comparison between all four subtype discovery methods. See Additional file 3: Figure S9B for the corresponding CoINcIDE network figure for (**a**)

Next, we compared CoINcIDE to concatenating all 17 datasets using only an intersecting 35-gene PAM50 feature set, as concatenation requires an intersection, as opposed to a union, feature set (see Methods for details and Additional file 1: Table S1 for the intersection gene set). CoINcIDE discovered meta-clusters of similar make-up to the PAM50 *de novo* analysis reported above, but it did not discover a HER2 meta-cluster and instead of 2 Basal meta-clusters, it discovered 2 Luminal A meta-clusters (Additional file 3: Figure S9A-B). Concatenated clustering with no between-dataset transformation, which acted as a baseline concatenation model, discovered three subtypes (clusters); these clusters were highly heterogeneous in terms of PAM50 subtype signal (Fig. 6b). Concatenated clustering with gene-wise batch mean centering (BMC) discovered 2 subtypes, one predominantly Basal and one mixed Luminal A, B, and HER2 (Fig. 6c). Concatenated clustering with ComBat discovered three subtypes, one predominantly Basal, one predominantly Luminal A, and one mixed Luminal B/HER2 (Fig. 6d). The AUC values for predicting pCR, RFS, and DFS each of these concatenated clusterings were all fairly similar and ranged from 0.583 to 0.606 (Additional file 1: Table S8 and Additional file 3: Figure S10D-H). The status of each patient’s PAM50 subtype status was also calculated after each transformation for comparison; patient PAM50 subtype status was heavily altered by the between-dataset transformation methods; BMC altered the PAM50 subtype classification of 821 patients and ComBat altered the classification of 673 patients when compared to the baseline PAM50 classifications (Additional file 3: Figure S11A-B). When the ComBat and BMC classification versions were directly compared, 848 patients’ classifications differed. We also analyzed whether a transformation like BMC would alter CoINcIDE results. When BMC was applied to each breast cancer dataset before running CoINcIDE using the full PAM50 gene set, CoINcIDE discovered a similar number of original input clusters (45) and final clusters in the CoINcIDE network (37) as the full CoINcIDE PAM50 analysis without BMC (44 and 38, respectively). CoINcIDE discovered six subtypes that were similar in PAM50 status make-up to the non-BMC CoINcIDE analysis but with two as opposed to one predominantly Luminal A subtype (Additional file 3: Figures S12 and 5B.) The predictive AUC values for pCR, RFS and DFS were 0.762, 0.660, and 0.630, respectively (see Additional file 1: Table S8 for details).

We then investigated whether the CoINcIDE *de novo* PAM50 analyses produced subtypes with similar prognostic significance as subtypes derived in a supervised manner using the PAM50 centroid classifications directly as patient subtypes (see Supplemental Methods in Additional file 2 for details). Transforming the datasets using BMC or ComBat before the supervised centroid analysis did not improve prognostic performance, and in some cases the performance decreased after these transformations (see Additional file 3: Figure S10I-Q and Additional file 1: Table S8 for details). CoINcIDE, using either the intersecting or full PAM50 gene set to derive *de novo* clusters for each dataset, performed comparably or better in terms of AUC compared to these supervised analyses (Additional file 1: Table S8).

Finally, as our last evaluation of CoINcIDE using the curated breast datasets, we ran *de novo* clustering analyses using meta-ranked gene sets based on gene expression variance without using PAM 50 genes (see Methods for details.) We derived gene sets of 50, 264, and 2,020 genes. All three non-PAM50 *de novo* CoINcIDE analyses produced subtypes with similar patient make-up as the PAM50 *de novo* CoINcIDE analysis (Additional file 3: Figure S13A, C, and E). All three of the non-PAM50 analyses were more predictive of outcomes than any of the concatenated clustering analyses (Additional file 1: Table S8); in particular, the non-PAM50 264-gene set CoINcIDE analysis produced subtypes with an AUC of 0.757 when combined with treatment status to predict pCR (Additional file 1: Table S8, Additional file 3: Figure S10U). This result is comparable to those of both the semi-supervised PAM50 centroid classification and *de novo* PAM50 CoINcIDE analyses and better than the models produced by the supervised PAM50 analyses (Additional file 1: Table S8).

### CoINcIDE identifies novel subtypes in ovarian cancer with prognostic significance and associated therapeutic targets

Next, we used CoINcIDE on the ovarian cancer datasets to discover novel subtypes using *de novo* meta-ranked gene sets in the same manner as the non-PAM50 CoINcIDE analyses (see Methods for details.) Two gene lists, one small (that is, 200 meta-ranked genes + 40 additional non-intersecting intra-rank genes) and one large (that is, 2,000 meta-ranked genes + 14 intra-ranked genes) were used, resulting in two CoINcIDE *de novo* clustering analyses (Additional file 1: Tables S6 and S7, respectively). These analyses incorporated 24 datasets from the curatedOvarianData collection [17].

**Fig. 7:**
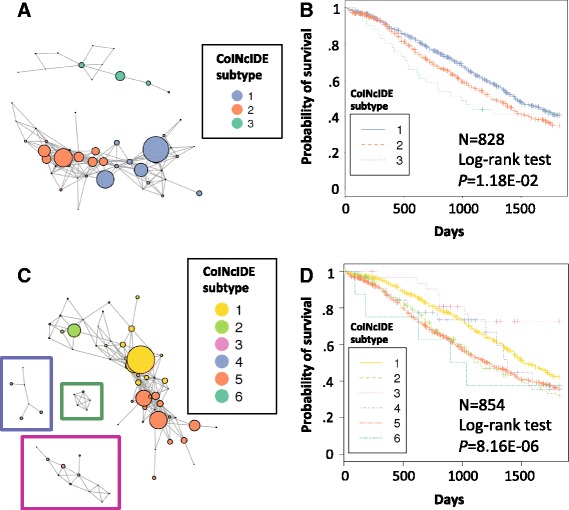
CoINcIDE ovarian short and long gene list meta-clusters. CoINcIDE results on 24 ovarian datasets from *de novo* clusterings using k-means consensus with the PAC score. **a**, **c** Resulting cluster-cluster networks for the short and long gene list clusterings, respectively. Node size is scaled to the relative number of samples in each cluster. **b**, **d** Five-year survival curves for the short and long gene list clusterings, respectively, as stratified by the final CoINcIDE subtypes depicted in (**a**) and (**c**). The symbols along the survival curves represent actual patient dataset points. A minimum mean similarity metric threshold of 0.5 was used to derive the long gene list results (Additional file 3: Figures S17 and S18 show results using a threshold of 0.7). N = number of events used in the model

Using the ovarian cancer 240-gene *de novo* clusters as inputs, CoINcIDE discovered three meta-clusters, or subtypes (Fig. 7a-b); subtypes 1 and 2 contained predominantly patient tumor samples with serous histology and subtype 3 contained mixed tumor histologies (Additional file 3: Figure S14A). From the TCGA dataset, two of the serous clusters were assigned to the two different serous subtypes, while the third serous cluster was not assigned any edges and thus was not included in the final network (Additional file 3: Figure S15A).

An effect size analysis was run on the dataset clusters assigned to each CoINcIDE subtype to identify marker genes for each subtype. A GSEA analysis using genes with an effect size of at least 0.5 for each subtype revealed distinctive signaling patterns; subtype 1 was enriched in gene sets for immune signaling, subtype 2 was enriched in gene sets for classic oncogenes such as *PTEN*, *P53*, and *KRAS* and cell development, and subtype 3 was enrichments in gene sets for VEGF and RAF pathways. Each subtype contained distinct potential drug target genes as defined by the Druggable Genome [31] with an effect size greater than 0.75 (Additional file 1: Tables S9 and S10). These subtypes significantly stratified patients both by overall survival and survival with a 5-year cutoff (Fig. 7b) (*P* values of 0.0479 and 0.0118, respectively). The mixed histology subtype contained sparse outcomes data and had the worst outcome (Fig. 7b).

Using the larger 2,014-gene set, CoINcIDE discovered six subtypes (Fig. 7c-d); subtypes 1 to 3 contained predominantly patient tumor samples with serous histology, subtypes 4 and 5 contained mixed histologies, and subtype 6 contained predominantly mucinous tissue samples; the three serous subtypes contained the majority of the patients in the network (Additional file 3: Figure S14B). One of the 24 input datasets was not used because it did not contain 80 % of these 2,014 meta-ranked genes, leaving 23 datasets for analysis (Additional file 3: Figure S15B).

We again used an effect size analysis to identify marker genes for each subtype and analyzed them using GSEA. This analysis revealed that subtype 1 was enriched in gene lists for immune signaling and Huntington’s and Parkinson’s disease, subtype 2 was enriched in gene lists for DNA repair and cell cycle signaling, subtype 3 was enriched in gene lists for classic oncogenes such as Notch and mTOR signaling, subtype 4 was enriched in gene lists for metabolism signaling, subtype 5 was enriched in gene sets for HIV/immune signaling, and subtype 6 was enriched in gene lists for apoptosis. Each subtype contained distinct potential drug target genes as defined by the Druggable Genome [31] with an effect size greater than 0.75 (Additional file 1: Tables S11 and S12).

These six ovarian cancer subtypes significantly stratified patients both by overall survival and survival with a 5-year cutoff (*P* values of 6.84E-06 and 8.16E-06, respectively). Because the non-serous subtypes contain sparse outcomes data, we cannot make strong inferences about the clinical utility of these subtypes, and thus we will focus our discussion on the three serous subtypes. The 5-year Kaplan Meier survival curve shows a distinct survival stratification between the serous immune and the serous DNA repair and oncogene subtypes (meta-clusters 1 versus 2 and 3, Fig. 7d). A similar analysis with only the three serous subtypes was also highly significant (figure not shown; *P* value of 7.93E-06). Meta-clusters 1, 2, 3, 4, and 5 contained 735, 200, 33, 32, and 789 patients, respectively; meta-cluster 6 contained only eight patients with outcomes data. When we removed meta-cluster 6 from the overall survival and survival analyses, the remaining five meta-clusters still significantly stratified patients (*P* values of 2.22E-06 and 2.97E-06, respectively).

To compare the prognostic significance of the full set of six CoINcIDE subtypes against that of standard clinical variables, we also ran univariate Cox proportional hazards models using histological type, tumor grade, and tumor stage. Histological type and tumor grade were weakly prognostic for overall survival (*P* values of 4.00E-02 and 3.26E-03, respectively) and with a 5-year cutoff (*P* values of 1.00E-01 and 1.30E-02, respectively). Tumor stage significantly stratified patients by both overall survival and with a 5-year cutoff (*P* values of 8.80E-12 and 1.35E-09, respectively). The CoINcIDE subtypes were not strongly defined by tumor stage (Additional file 3: Figure S16), suggesting that CoINcIDE captures novel clinically relevant signal.

When the second global density curve maxima similarity threshold of 0.7 (Additional file 3: Figure S3D) was used, CoINcIDE discovered seven subtypes; 5/7 of these subtypes had greater than three datasets and closely mirrored the subtypes found in the 0.5 threshold analysis, including the three large serous clusters (Additional file 3: Figure S17A-B). These subtypes significantly predicted overall and 5-year cutoff continuous outcomes (Additional file 3: Figure S18) with *P* values of 2.52E-04 and 3.46E-04, respectively. Several of these meta-clusters (meta-clusters 5, 6, and 7) had less than 30 outcomes recorded; if these clusters are removed, the overall and 5-year cutoff survival *P* values were higher, but still significant at 6.63E-03 and 2.81E-03, respectively.

We further confirmed the stability of the three serous meta-clusters from the large gene list CoINcIDE analysis (using the initial 0.5 similarity threshold) by removing the TCGA dataset and re-running CoINcIDE. Removing this large, high-quality dataset did not change any of the other dataset clusters’ final assignments in the three serous subtypes (Additional file 3: Figure S19A-B); the assignments for the three non-serous subtypes also did not change because none of these contained clusters from the TCGA dataset. These CoINcIDE subtypes without the TCGA dataset still significantly stratified patients by overall survival and with a 5-year cutoff (*P* values of 1.05E-04 and 3.67E-05, respectively).

## Discussion

We present here CoINcIDE, a framework for discovery of patient subtypes across multiple datasets. The simulated tissue cluster data results show that CoINcIDE performs well with reasonable noise levels, clusters with differing sample sizes, and datasets with differing numbers of clusters. The TPR slightly decreased for simulations that allowed datasets to have a single cluster, in part because CoINcIDE does not allow for the comparison of clusters that are both derived from datasets with only one cluster. CoINcIDE is conservative in that it maintains a low FPR even as noise levels increase; even when the minimum mean similarity threshold was set to 0.0, the FPR rate never increased above 10 %. CoINcIDE also does not assign edges between low-quality, noisy clusters, as seen in the final simulation where 50 % of the clusters were samples randomly selected from all tissue types (Additional file 3: Figures S5G and S6G). These results give us confidence that CoINcIDE can identify true subtypes in scenarios that mimic real-life datasets with varying noise levels, numbers of clusters, and sizes of clusters.

The CoINcIDE PAM50 centroid cluster breast cancer analysis re-discovered the PAM50 subtypes, confirming CoINcIDE’s accuracy on a large database of real gene expression datasets. We acknowledge that completely accurate PAM50 subtypes can only be achieved using the commercially available platform, nor are the PAM50 subtypes a ground truth, but simply a set of subtypes known to be replicable [32]. However, using the PAM50 centroids to assign patients to PAM50 subtypes, we have illustrated that CoINcIDE accurately captures known clinically relevant subtypes. Additionally, the pCR, or treatment response, AUC of 0.762 closely matches the reported AUC of 0.78 from the initial PAM50 publication [3], showing that CoINcIDE also produces meta-clusters with expected correlative patterns to an external response variable.

The CoINcIDE breast cancer networks discovered also reflect known hormonal signaling patterns in breast cancer; for example, the full PAM50 set *de novo* clustering Basal meta-cluster was highly separately from the other meta-clusters, and contained tightly interconnected clusters (Fig. 3a). This trend was also seen throughout the non-PAM50 gene set analyses (Additional file 3: Figures S11A, C, and E). Basal breast tumors are considered to be fairly distinct from other tumor subtypes, in terms of treatment response, gene expression, and mutation patterns [33]. Clusters of various sizes and hormonal status, from various microarray platforms with various mixtures of subtypes, were included in the final breast cancer networks, highlighting CoINcIDE’s ability to overcome dataset-specific noise without any additional dataset transformations beyond baseline intra-dataset normalization.

The CoINcIDE meta-cluster network visualizations are an intuitive data exploration tool to help researchers identify such potential biases. For example, although it did not heavily affect the final overall meta-clusters, the two-channel dataset clusters in the breast analyses had far fewer edges assigned to other clusters than the one-channel dataset clusters (this platform bias was not observed in the ovarian cancer networks.) It is also easy to identify a highly replicable, robust subtype as a meta-cluster with many nodes (clusters) tightly connected by many edges with very few edges spanning across to other meta-clusters.

The PAM50 gene set *de novo* cluster CoINcIDE analysis using k-means consensus clustering showed that CoINcIDE can discover biologically intuitive signal when each dataset is *de novo* clustered in an automatic, unbiased fashion. A consistent pattern seen in all breast cancer analyses was that the CoINcIDE subtypes discovered tended to be more significantly prognostic (that is, lower *P* values) in outcomes models for treatment response (pCR) than long-term outcomes (RFS or DFS); in fact, the *de novo* clustering analysis achieved the same pCR AUC as the PAM50 centroid clustering pCR AUC (Additional file 1: Table S8).

The concatenated dataset analyses show how transformation techniques to remove dataset-specific artifacts can significantly alter actual biological signal found in a dataset. This emphasizes the importance of methods like CoINcIDE that require no between-dataset normalization, especially when researchers are searching for finer-grained subtypes whose assignments may change heavily with any transformations. However, while applying no transformation, BMC or ComBat resulted in highly different concatenated clustering results (Fig. 6), applying BMC to each individual dataset did not heavily alter CoINcIDE’s results, suggesting that CoINcIDE is also reasonably robust to different normalization procedures. CoINcIDE also discovered either clearer subtypes in terms of known PAM50 signal and/or presented more finer-grained subtypes for greater exploratory analyses in comparison to the concatenated analyses. CoINcIDE also identified a potential outlier (or highly distinct) dataset whose clusters were never assigned edges to any clusters in other datasets for any gene set tested. This dataset did not fall out as a separate cluster in any of the concatenated clusterings, but rather was smoothed over the different subtypes.

Similar AUC results as those from the PAM50 *de novo* CoINcIDE analysis were achieved when using *de novo* meta-ranked gene sets that did not include the PAM50 gene set; these results outperformed all of the concatenated clustering results, regardless of whether BMC or ComBat was applied (Additional file 1: Table S8). These prognostic results using CoINcIDE also performed comparably, and oftentimes better, than a completely supervised approach using the PAM50 centroid classifications, with or without transforming the data *a priori* using BMC or ComBat (Additional file 1: Table S8). We acknowledge that these results do not reflect the accuracy or usefulness of the commercial PAM50 platform performed in a controlled laboratory setting; rather, they show that CoINcIDE can produce subtypes with similar prognostic significance than those derived from much more controlled supervised methods. It also appears that for supervised analyses, transformations such as BMC or ComBat have negligible effects in terms of producing subtypes that can accurately predict outcomes (when combined with treatment information), but these transformations have much stronger negative effects when unsupervised cluster analyses are used to derive the subtypes. However, the latter approach is oftentimes needed when a researcher or clinician is not aware *a priori* of the key patient subtypes in a disease.

An additional benefit to CoINcIDE over concatenated dataset analyses includes shorter analysis run-times; k-means clustering on a large concatenated dataset can be computationally intensive. Finally, CoINcIDE also provides metrics to interpret the replicability and quality for each specific subtype. The CoINcIDE R package reports the number of datasets with clusters assigned to each subtype that passed significance and similarity thresholds, and how many edges for that subtype were assigned to clusters within the same subtypes. A user can easily interpret not only these quantitative metrics, but also the resulting network visualizations. The CoINcIDE package includes between-dataset normalization methods like ComBat so that they can be compared alongside a CoINcIDE analysis.

The ovarian analyses showed that CoINcIDE can provide important biological insight into a disease for which clear gene expression subtypes have proven elusive [34]; this ambiguity in signal is manifested in the CoINcIDE networks for both the short and long gene list analyses that have several edges spanning different meta-clusters (Fig. 7a and c). However, the effect size analyses on each CoINcIDE meta-cluster provide biological hypotheses for which genes may be the most robust in differentiating ovarian subtypes, in particular the serous subtypes. For example, in the short-gene list CoINcIDE ovarian analysis, the Druggable Genome [31] gene *CYP4B1* had the highest effect size (0.676, Additional file 1: Table S9) in the serous meta-cluster 1. The expression of this gene has been confirmed stratify non-platinum-resistant serous ovarian cancer patients that do not and do have recurrences after a taxane regimen [35]. While this effect size is relatively modest, it is a robust measurement across several datasets, and suggests that *CYP4B1* may provide insights into therapy for ovarian cancer patients tailored by subtype. In the long-gene list CoINcIDE ovarian subtypes, *CYP4B1* had an even higher effect size (0.853, Additional file 1: Table S11) for the serous meta-cluster 1, further highlighting its potential use as a druggable target.

The other two serous meta-clusters from the long-gene list analysis, meta-clusters 2 and 5, had poorer outcomes in general (Fig. 7d). CoINcIDE also identified potential druggable targets within each of these meta-clusters, such as the gene *ODC1* which has an effect size of 1.092 in meta-cluster 2 (Additional file 1: Table S11) There is not extensive literature on *ODC1* in serous ovarian cancer, but it has been suggested that this gene’s expression decreases when platinum is added to ovarian cancer cell lines [36], suggesting *ODC1* may indeed have therapeutic relevance for meta-cluster 2. Beyond providing druggable hypotheses, these CoINcIDE subtypes (meta-clusters) driven by these effect size patterns also significantly stratify patients by length of survival (Fig. 7d), giving us further confidence in their clinical utility.

CoINcIDE also highlighted the shortcomings of using only a single dataset such as the TCGA dataset to cluster tissue samples. For example, we observed two persistent serous subtypes across both the small and large gene list ovarian analyses; the long gene list analysis produced a third serous subtype that incorporated the third TCGA serous cluster not included in the short gene list CoINcIDE network. This third subtype contained far fewer clusters and less edges interconnecting the clusters, suggesting that this third meta-cluster should be viewed as more exploratory, or that the gene set used does not capture the signal in this meta-cluster as clearly as in the two other serous meta-clusters.

The initial TCGA ovarian publication reported four serous subtypes: ‘immunoreactive’, ‘proliferative’, ‘mesenchymal’, and ‘differentiation’ [37]; the three serous meta-clusters discovered by the CoINcIDE long-gene list analysis follow roughly similar trends in that one was enriched in immune response, one in DNA repair and one in classical oncogene signaling. Recent TCGA analyses on the Broad Institute’s GDAC website [38] that used non-negative matrix factorization and hierarchical consensus clustering suggest that there are three, as opposed to four, stable serous ovarian meta-clusters, the same number we discovered in our long-gene set analysis (see the caption of Additional file 3: Figure S20 for data details.) The CoINcIDE serous ovarian meta-cluster patient assignments roughly correspond to these recent GDAC webserver patient assignments, but they are not identical (Additional file 3: Figure S20). The differences in the number of clusters and enrichment patterns between the initial TCGA published subtypes, the GDAC webserver results and the CoINcIDE subtypes reflect a broader, continued debate on the number and replicability of ovarian serous subtypes. A recent report suggests that serous ovarian cancer subtypes are a ‘holy grail’ [34]. We acknowledge that further work, especially *in vivo* cell line tests, must be done to validate the ovarian subtypes we have discovered using CoINcIDE, but their prognostic significance across multiple datasets provides a robust platform from which to validate them further.

More importantly, the TCGA ovarian publication stated that the reported four expression subtypes did not significantly stratify TCGA patients by survival duration [37], while the CoINcIDE subtypes for both the small and large gene list analyses significantly stratified patients by overall survival, not just for the TCGA dataset, but across several datasets. Outcomes data were sparse for the mixed histology subtypes, and thus broad survival trends for these subtypes cannot be readily inferred, but the CoINcIDE analysis still provided robust effect sizes of genes that distinguish these subtypes from the serous subtypes. A stand-alone TCGA analysis would not have provided this information, because it would have only contained samples with serous histology.

Additionally, we showed that the CoINcIDE subtypes remained the same upon removal of the TCGA dataset, emphasizing that multiple smaller datasets can provide stable CoINcIDE subtypes. The success of CoINcIDE in finding subtypes that are both biologically intuitive and predictive of survival is of course dependent upon the input feature (gene set) and the single-dataset clustering algorithm, but it is an effective framework to confirm that the resulting subtypes are highly replicable across numerous datasets and thus stand a chance of being implemented one day in routine clinical practice.

## Conclusions

In the era of big data, multiple datasets have now been collected for a single disease, necessitating meta-analysis cluster frameworks. CoINcIDE harnesses the power of multiple datasets to provide novel approaches for unsupervised clustering across multiple datasets. CoINcIDE has the ability to discover both replicable and prognostically significant subtypes without any additional dataset-specific transformations, unlike the current established method of concatenation. CoINcIDE also does not require a strictly intersecting feature set across all datasets, and the final meta-cluster network provides a rich platform to infer both potential meta-cluster and dataset-specific trends.

Finally, the framework proposed here is not limited to gene expression data; CoINcIDE can be implemented using various clustering algorithms, and even distance, as opposed to similarity, metrics. The CoINcIDE framework and the breast cancer collection are available as R packages to the community; it is our hope that these resources and methods provide a platform for further development of novel methods to cluster across multiple datasets.

### Software and data availability

Both curatedBreastData and CoINcIDE are available as R packages. The curatedBreastData package can be downloaded from http://bioconductor.org/packages/release/data/experiment/html/curatedBreastData.html and original data processing scripts can be found at https://github.com/kplaney/curatedBreastCancer. The CoINcIDE R package can be found at https://github.com/kplaney/CoINcIDE.

## Additional files

Additional file 1: All supplementary tables (labeled with the prefix ‘S’ in the main manuscript) and their corresponding legends. (PDF 1829 kb) Additional file 2: Additional methods details to supplement the main Methods section. (PDF 471 kb) Additional file 3: All supplementary figures (labeled with the prefix ‘S’ in the main manuscript) and their corresponding legends. (PDF 7043 kb)

## Abbreviations

CoINcIDE: Clustering Intra and INter DatasEts
DFS: disease-free survival
ER: estrogen
GEO: Gene Expression Omnibus
HER2: human epidermal growth factor 2
pCR: pathological complete response
RFS: relapse-free survival
TCGA: The Cancer Genome Atlas
