# Supplementary material for: CoINcIDE: A framework for discovery of patient subtypes across multiple datasets
Source: Genome Med. 2016 Mar 9;8:27. doi: 10.1186/s13073-016-0281-4 (PMC4784276; doi:10.1186/s13073-016-0281-4)
Supplement: Additional file 1: — All supplementary tables (labeled with the prefix ‘S’ in the main manuscript) and their corresponding legends. (PDF 1829 kb) [file 13073_2016_281_MOESM1_ESM.pdf]

Additional file 1

Supplementary Tables

Supplementary Table 1: PAM50 intersecting gene list. The list of 35 genes from the full 50-gene PAM50 feature set that were present across all 17 breast cancer datasets.

Supplementary Table 2: Comparison of methods to select the number of clusters within a dataset. These tables summarize the number of clusters, as compared with the expected number of clusters, for several datasets. The expected number of clusters for each dataset was estimated using provided immunohistochemistry (IHC) ER and HER2 status and/or commercial Pam50 platform results; 8 datasets that had this data were used to select the optimal clustering and select K method. As seen in the results below, Hartigan Wong's k-means consensus clustering with 1 random start and with 90% resampling of samples for each of the 500 iterations with a rounded PAC score discovered a total number of clusters for each dataset consistent with the expected number of clusters.

8 clustering and select K number of cluster methods combinations were evaluated for each dataset:

1. km_short_Nstart15_pItem9: k-means using the restricted 35-gene PAM50 gene set with nstart=15 (15 random starts). If consensus clustering was run, pItem=.9, or 90% of the patients were resampled for each iteration.
2. km_short_Nstart1_pItem9: k-means using the restricted 35-gene PAM50 gene set with nstart=1 (1 random start). If consensus clustering was run, pItem=.9, or 90% of the patients were resampled for each iteration.
3. km_short_Nstart15_pItem8: k-means using the restricted 35-gene PAM50 gene set with nstart=15 (15 random starts). If consensus clustering was run, pItem=.8, or 80% of the patients were resampled for each iteration. For hierarchical clustering, this result is equivalent to the km_short_Nstart15_pItem9 result.
4. hc_short_pItem9: Hierarchical clustering using the using the restricted 35-gene PAM50 gene set. If consensus clustering was run, pItem=.9, or 90% of the patients were resampled for each iteration.
5. hc_short_pItem8: Hierarchical clustering using the using the restricted 35-gene PAM50 gene set. If consensus clustering was run, pItem=.8, or 80% of the patients were resampled for each iteration.
6. km_full_Nstart15_pItem9: k-means using full number of PAM50 genes found in each dataset with nstart=15 (15 random starts). If consensus clustering was run, pItem=.9, or 90% of the patients were resampled for each iteration.
7. km_full_Nstart1_pItem9: k-means using the full number of PAM50 genes found in each dataset with nstart=1 (1 random start). If consensus clustering was run, pItem=.9, or 90% of the patients were resampled for each iteration.
8. hc_full_pItem9: Hierarchical clustering using the full number of PAM50 genes found in each dataset If consensus clustering was run, pItem=.9, or 90% of the patients were resampled for each iteration.

Supplementary Table 3: The full list of meta-ranked genes used to cluster each dataset (excluding genes that were not present in a specific dataset) for the non-PAM50 50-gene feature set CoINcIDE analysis. See the Supplemental Methods in Additional File 3 for more details on the meta-ranking algorithm.

Supplementary Table 4: The full list of meta-ranked genes used to cluster each dataset (excluding genes that were not present in a specific dataset) for the non-PAM50 264-gene feature set CoINcIDE analysis. See the Supplemental Methods in Additional File 3 for more details on the meta-ranking algorithm.

Supplementary Table 5: The full list of meta-ranked genes used to cluster each dataset (excluding genes that were not present in a specific dataset) for the non-PAM50 2020-gene feature set CoINcIDE analysis. See the Supplemental Methods in Additional File 3 for more details on the meta-ranking algorithm.

Supplementary Table 6: The full list of meta-ranked genes used to cluster each dataset (excluding genes that were not present in a specific dataset) for the ovarian short gene list CoINcIDE analysis. See the Supplemental Methods in Additional File 3 for more details on the meta-ranking algorithm.

Supplementary Table 7: The full list of meta-ranked genes used to cluster each dataset (excluding genes that were not present in a specific dataset) for the ovarian long gene list CoINcIDE analysis. See the Supplemental Methods in Additional File 3 for more details on the meta-ranking algorithm.

Supplementary Table 8: AUC results for prediction of pCR, RFS and DFS for breast cancer patients using patient subtypes and the treatment each patient had in logistic regression models. Star symbols denote the p-values for the Chi-squared contrast test run for each model comparing the predictive significance of adding the patients' subtypes on top of the baseline treatment variable-only model; * denotes p<=0.05, ** denotes p<=0.005 and *** denotes p<=2.2E-16. See Supplemental Methods for details on treatment variables and the binary outcomes models. The column names are the type of model; for example, pCR~M +Rx denotes a model predicting binary pCR using meta-cluster (subtype) status and treatment status. "Baseline" is used to denote where no batch effect transformations were applied for the concatenated and supervised analyses. Unless noted, no transformation was used for the CoINcIDE analyses. "Full" in terms of the PAM50 gene set denotes that all available PAM50 genes were used and "intersecting" denotes the 35-gene set from the full PAM50 gene set that was found in all datasets (the batch effect methods gene-wise Batch Mean Centering (BMC) and ComBat methods require all genes to be found in all datasets.) Data was not available for the concatenated clustering BMC analysis predicting pCR because only one of the two subtypes contained patients with recorded pCR values, meaning subtype status could not be used as a differentiating variable in a linear model.

Supplementary Table 9: Effect sizes of druggable genes by meta-cluster for ovarian meta-rank short gene list analysis. Hedge's g mean difference effect sizes from the CoINcIDE analysis for genes specifically from the Druggable Genome by Hopkins and Groom summarize how relatively overexpressed, in terms of logged expression values, a gene is for patients within a certain meta-cluster, as compared to patients in other meta-clusters. M1=meta-cluster one, etc. # datasets M1 = the number of datasets used to calculate the effect size for this gene in meta-cluster one. The top 10 genes in terms of effect size above a minimum 0.5 threshold are reported for each meta-cluster (unless there were less than 10 such genes.) NA = effect size was below 0.5.

Supplementary Table 10: The full list of druggable genes with an effect size of at least 0.5 for all subtypes from the ovarian short gene list CoINcIDE analysis (Table S9 provides only the top-ranking genes for each subtype for easier interpretation.) See the legend for Table S9 for more details on the interpretation of the effect size.

Supplementary Table 11: Effect sizes of druggable genes by meta-cluster for ovarian long meat-rank gene list analysis. Hedge's g mean difference effect sizes from the ovarian long gene list CoINcIDE analysis for genes from the Druggable Genome by Hopkins and Groom are reported. See Table S9 for details on column names and interpretation.

Supplementary Table 12: The full list of druggable genes with an effect size of at least 0.5 for all subtypes from the ovarian long gene list CoINcIDE analysis (Table S11 provides only the top-ranking genes for each subtype for easier interpretation.) See the legend for Table S9 for more details on the interpretation of the effect size.

Supplementary Table 13: Dataset information for the PAM50 semi-supervised centroid CoINcIDE analysis. The number/label used in Figures such as Figure S7 in Additional File 1 is provided along with original dataset ID. The number of dataset-specific clusters as determined by k-means consensus clustering is also provided (these cluster assignments were used as inputs to derive cluster-cluster similarity and significance metrics.)

Supplementary Table 14: Dataset information for the PAM50 de novo unsupervised CoINcIDE analysis. See Table S13 for details on the column headers. The number/label corresponds to the node labels in Figure S8 in Additional File 1.

Supplementary Table 15: Dataset information for the PAM50 intersecting (35-gene) feature set de novo unsupervised CoINcIDE analysis used to compare results against concatenated clustering. See Table S13 for details on the column headers; Figure S9-B in Additional File 1 provides summary visualizations for this analysis.

Supplementary Table 16: Dataset information for the non-PAM50 50-gene feature set de novo unsupervised CoINcIDE analysis used to compare results against concatenated clustering. See Table S13 for details on the column headers; Figure S13A-B in Additional File 1 provides summary visualizations for this analysis.

Supplementary Table 17: Dataset information for the non-PAM50 264-gene feature set de novo unsupervised CoINcIDE analysis used to compare results against concatenated clustering. See Table S13 for details on the column headers; Figure S13C-D in Additional File 1 provides summary visualizations for this analysis.

Supplementary Table 18: Dataset information for the non-PAM50 2020-gene feature set de novo unsupervised CoINcIDE analysis used to compare results against concatenated clustering. See Table S13 for details on the column headers; Figure S13E-F in Additional File 1 provides summary visualizations for this analysis.

Supplementary Table 19: Dataset information for the ovarian cancer short gene list CoINcIDE de novo clustering analysis. See Table S13 for details on the column headers. The number/label used in Figures such as Figure S15A in Additional File 1 is provided along with original dataset ID.

Supplementary Table 20: Dataset information for the ovarian cancer long gene list CoINcIDE de novo clustering analysis. See Table S13 for details on the column headers. The number/label used in Figures such as Figure S15B in Additional File 1 is provided along with original dataset ID.

Supplementary Table 1

**Genes**
GRB7
CEP55
MYBL2
KRT5
CDC20
UBE2C
CDH3
EXO1
MELK
MIA
KRT14
BAG1
BIRC5
BLVRA
CCNB1
CCNE1
CDC6
CENPF
EGFR
ERBB2
ESR1
FOXA1
FOXC1
KIF2C
KRT17
MAPT
MDM2
MKI67
MMP11
MYC
PGR
RRM2
SFRP1
SLC39A6
TYMS

Supplementary Table 3

Genes
SCGB2A2
TFAP2B
SCGB1D2
LTF
PIP
S100P
NPY1R
TFF3
CXCL13
PEG10
SCUBE2
KRT15
CXCL9
CEACAM6
UBD
TFF1
KRT23
SCGB2A1
PROM1
FABP4
AQP3
APOD
S100A8
VTCN1
IGKV3D-15
STC2
IGKC
SLPI
CA2
ALDH3B2
BAMBI
HLA-DQA1
S100A9
COL11A1
TMC5
MUC1
AGR2
ABAT
CHI3L1
FGFR3
DNAJC12
GSTM3
DUSP4
CXCL10
HLA-DQB1
SPP1
MAOB
IFIT1
ADH1B
CXCL14

Supplementary Table 2

GSE2034: expected number of clusters: between 2 and 4					
**Clustering method**	**Gap Test**	**Consensus Fraction**	**Mean Consensus**	**PAC**	**Rounded PAC**
km_short_Nstart15_pItem9	5	2	2	3	3
km_short_Nstart1_pItem9	4	2	2	3	3
km_short_Nstart15_pItem8	5	2	2	3	3
hc_short_pItem9	2	2	2	2	2
hc_short_pItem8	2	2	2	2	2
km_full_Nstart15_pItem9	7	4	4	4	4
km_full_Nstart1_pItem9	1	4	4	4	4
hc_full_pItem9	2	2	2	2	2

GSE25055_MDACC_M: expected number of clusters: between 3 and 5					
**Clustering method**	**Gap Test**	**Consensus Fraction**	**Mean Consensus**	**PAC**	**Rounded PAC**
km_short_Nstart15_pItem9	8	2	2	2	9
km_short_Nstart1_pItem9	5	2	2	3	3
km_short_Nstart15_pItem8	8	2	2	2	2
hc_short_pItem9	2	2	2	2	2
hc_short_pItem8	2	2	2	2	2
km_full_Nstart15_pItem9	8	2	2	2	2
km_full_Nstart1_pItem9	2	2	2	2	2
hc_full_pItem9	2	2	2	2	2

GSE22226_GPL1708: expected number of clusters: between 4 and 5					
**Clustering method**	**Gap Test**	**Consensus Fraction**	**Mean Consensus**	**PAC**	**Rounded PAC**
km_short_Nstart15_pItem9	3	4	2	3	4
km_short_Nstart1_pItem9	2	4	2	4	4
km_short_Nstart15_pItem8	3	4	2	4	4
hc_short_pItem9	1	2	2	2	2
hc_short_pItem8	1	2	2	2	2
km_full_Nstart15_pItem9	3	3	2	2	5
km_full_Nstart1_pItem9	3	5	2	5	5
hc_full_pItem9	1	2	2	2	2

GSE20181: expected number of clusters: between 1 and 2					
**Clustering method**	**Gap Test**	**Consensus Fraction**	**Mean Consensus**	**PAC**	**Rounded PAC**
km_short_Nstart15_pItem9	1	2	2	2	2
km_short_Nstart1_pItem9	1	2	2	2	2
km_short_Nstart15_pItem8	1	2	2	2	2
hc_short_pItem9	1	2	2	2	2
hc_short_pItem8	1	2	2	2	2
km_full_Nstart15_pItem9	1	2	2	2	2
km_full_Nstart1_pItem9	1	2	2	2	2
hc_full_pItem9	1	2	2	2	2

GSE19615: expected number of clusters: 4					
**Clustering method**	**Gap Test**	**Consensus Fraction**	**Mean Consensus**	**PAC**	**Rounded PAC**
km_short_Nstart15_pItem9	4	2	2	2	2
km_short_Nstart1_pItem9	4	2	2	2	4
km_short_Nstart15_pItem8	4	2	2	2	2
hc_short_pItem9	3	4	3	4	4
hc_short_pItem8	3	4	3	4	4
km_full_Nstart15_pItem9	4	2	2	2	2
km_full_Nstart1_pItem9	4	2	2	2	2
hc_full_pItem9	3	9	3	3	3

GSE16446: expected number of clusters: 2					
**Clustering method**	**Gap Test**	**Consensus Fraction**	**Mean Consensus**	**PAC**	**Rounded PAC**
km_short_Nstart15_pItem9	3	2	2	2	2
km_short_Nstart1_pItem9	3	2	2	2	2
km_short_Nstart15_pItem8	3	2	2	2	2
hc_short_pItem9	2	4	2	1	1
hc_short_pItem8	2	4	2	1	1
km_full_Nstart15_pItem9	2	3	3	3	3
km_full_Nstart1_pItem9	2	3	3	3	3
hc_full_pItem9	2	2	2	2	2

GSE12093: expected number of clusters: between 1 and 2					
**Clustering method**	**Gap Test**	**Consensus Fraction**	**Mean Consensus**	**PAC**	**Rounded PAC**
km_short_Nstart15_pItem9	1	3	3	3	3
km_short_Nstart1_pItem9	1	3	3	3	3
km_short_Nstart15_pItem8	1	3	3	3	3
hc_short_pItem9	1	2	2	1	1
hc_short_pItem8	1	2	2	1	1
km_full_Nstart15_pItem9	3	4	3	3	3
km_full_Nstart1_pItem9	3	4	4	3	3
hc_full_pItem9	1	10	2	1	1

GSE25065_MDACC: expected number of clusters: between 3 and 5					
**Clustering method**	**Gap Test**	**Consensus Fraction**	**Mean Consensus**	**PAC**	**Rounded PAC**
km_short_Nstart15_pItem9	3	2	2	2	2
km_short_Nstart1_pItem9	2	2	2	2	2
km_short_Nstart15_pItem8	3	2	2	2	2
hc_short_pItem9	2	2	2	2	2
hc_short_pItem8	2	2	2	2	2
km_full_Nstart15_pItem9	3	2	2	2	2
km_full_Nstart1_pItem9	3	2	2	2	2
hc_full_pItem9	2	2	2	2	2

Supplementary Table 4

**Genes**				
COL11A1	COMP	CRABP2	NQO1	MT1G
MMP7	CCND1	S100A4	SEMA3C	HOXB5
DEFB1	RGS1	FGFR3	SPRY2	MAG
C7	HOXD3	INHBB	GREB1	MYH11
MAL	IFI6	MGP	FILIP1L	CCL21
LUM	CTGF	GAS1	NDN	IRX5
SST	CFB	CD200	ALDH1A1	VSNL1
NNMT	ID1	SFRP1	PTX3	HOXA5
VCAN	LOX	FGF9	KRT7	TGFA
MFAP5	SERPINA1	GSTT1	HLA-DPA1	HTR3A
INHBA	SERPINE2	TNFSF10	NUAK1	TUBB4A
CDKN2A	SCG5	NPTX2	CCNE1	SPARC
CXCL10	GATA6	FXYD3	IGFBP4	SERPINE1
FOS	BCAT1	PLAT	ST6GALNAC2	COL6A1
KLK10	CDH11	RGS2	TFAP2C	PDE6B
CHI3L1	ACTA2	HIST1H1C	CXCL1	CXCL11
TFAP2A	FAP	ADM	NR4A2	S100P
GPX3	TSPAN8	NID2	SPON1	ST13
RARRES1	IDO1	CRIP1	ZNF423	CITED2
TAGLN	MMP9	ISLR	SFN	IL6
CYP4B1	TNC	RARRES3	PMP22	CD9
S100A8	MX1	CXCL9	LY6E	ABCA4
IGF2BP3	CXCL2	SLPI	CBS	NEFH
KLK7	NMU	CDH2	TNFAIP2	NTRK2
TFPI2	CYP1B1	COL6A2	CD163	IL10RB
PNOC	SERPINA5	IFI44	SDC1	SOCS3
TNNT1	MMP2	BST2	KAL1	RASA2
CDH6	CLU	ATF3	SPP1	TROAP
KLK6	CTSK	COL6A3	C1R	STAR
GLDC	TSPAN7	SULT1C2	MATN2	STXBP1
MSLN	S100A9	IGFBP2	AKAP12	GRIK3
UCHL1	S100A1	SNAI2	THY1	TNFAIP6
HOXB6	ATP6V1B1	PTGS1	COL15A1	BMP7
ISG15	PDGFRA	WT1	FBLN1	SLC15A2
APOA1	CXCL12	C1S	BIRC3	EPHA5
CLDN10	CCL20	C1QB	GABRE	TNFRSF8
FOLR1	LAMB1	PLAU	GSTM3	DDC
IL8	HBB	RBP1	EFS	AGR2
CCNA1	ID4	GBP1	OAS2	EPYC
VCAM1	GPNMB	LOXL1	MEOX1	MPZ
CP	COL5A1	TIMP3	NPR1	
COL3A1	HMGA2	SOX9	GPM6B	
S100A2	DUSP1	RARRES2	COL1A2	
SERPINF1	CYR61	HLA-DPB1	AR	
ERBB4	CRYAB	IFI27	HIST2H2BE	
COL10A1	GJA1	GDF15	IFIT3	
LAMA3	TDO2	TRIM29	OAS1	
FN1	PPAP2C	CCL5	BGN	
MMP11	FOSB	APOE	FHL1	
AEBP1	ACTG2	SPOCK2	COL9A2	

Supplementary Table 5

Genes

COL11A1	COL9A2	CDKN3	ATP6V1C1	AIF1	RAD23B	PGRMC1	ILF3	TBCD	ZNF652	MAG
MMP7	PHLDA2	MNDA	PIK3R1	HRH1	DYRK2	GRB14	CEBPB	CCBL2	NFATC1	CCL21
DEFB1	PDZRN3	ANPEP	IER2	AP1S2	MYL6B	DCXR	ATP5G1	CTBS	CDK5	VSNL1
C7	CSTA	ELOVL6	SETBP1	UNG	CDKN2B	APPL2	LST1	APEX1	PLA2G4C	TUBB4A
MAL	ANXA3	UBE2C	TLE2	MAOA	AGA	KNTC1	NCOA2	YARS	CDC6	PDE6B
LUM	BAMBI	MARCKS	NEFH	GSTA4	LPGAT1	ACVR1	SDHB	WNK1	TCEA1	ABCA4
SST	TGFBI	PCSK5	PRNP	ITGB1BP1	ILF2	FASN	ASNA1	WFS1	CYB5B	NTRK2
NNMT	LPHN2	PAM	LMNB1	PSD3	TOMM20	RAD21	CHEK1	RSU1	DAP	RASA2
VCAN	DPYD	FABP6	TIMP2	PDLIM7	SP110	SCG2	CPQ	EHHADH	MR1	GRIK3
MFAP5	CSRP2	LTBP1	IGFBP7	KCNH2	AGL	EML1	PTP4A1	CUL4A	PRCP	EPHA5
INHBA	SRGN	IFIT2	CCR1	IL6	EFNB3	SMAD6	ARHGEF10	CCDC28A	ATF1	TNFRSF8
CDKN2A	NRCAM	PPIC	IL4R	ADARB1	EPHA1	MRPS27	DLD	LRRC23	FUBP1	DDC
CXCL10	TPM2	MAOB	TACC2	HMGCR	AHNAK	BNIP3L	TXNDC9	SS18L1	PPP2CA	EPYC
FOS	EGR1	A2M	AADAC	RABAC1	ME3	MAN2A1	KDELR1	GUK1	TAF7	MPZ
KLK10	PDGFRL	NDP	VWF	FBL	PLAGL2	CASP8	MAP7	PIK3C2B	SLC33A1	
CHI3L1	DPYSL3	SERPINB1	MSH2	SEPW1	PNN	CASP4	TPST1	RTN4	PSME1	
TFAP2A	TOP2A	DSC2	PRDX2	PPIF	MERTK	SLC30A1	SNCA	CAPN9	IARS2	
GPX3	ALCAM	SPOCK1	NET1	DST	HSPA13	DYNLT3	HMGN3	MAP4K5	GNL2	
RARRES1	LAMB3	PPAP2B	PDLIM1	NR2F6	CYFIP2	RCN1	OVGP1	GSTZ1	PPP2R5E	
TAGLN	HPN	CITED2	IQGAP2	PUF60	KDELR2	LRRC32	ITPKB	ALG5	SLC1A4	
CYP4B1	TLE4	FGFR2	AQP3	RAC2	LGALS3BP	NCAPH	ZNF195	GSTP1	LGALS2	
S100A8	SRPX	LRP8	PHLDA1	MEF2C	PGK1	NAT1	PTPN14	TNPO1	KEAP1	
IGF2BP3	DUSP5	GCH1	CRIM1	GCLM	GFPT1	CLIP2	NUDT1	REEP5	GNB5	
KLK7	RBPMS	ERBB3	ROR1	L1CAM	SIX1	AFF1	LSP1	TIA1	SRPR	
TFPI2	EMP1	MTSS1	FSCN1	PFKM	GLRX	PKM	IL2RB	AGPS	CTR9	
PNOC	HTRA1	OPLAH	SLC7A1	TGFB2	NAP1L3	MED21	VGLL1	UQCRC2	NCBP1	
TNNT1	KRT5	MELK	GADD45A	SKIL	SEMA3F	MPDZ	RPS6KA5	TOP2B	CDC42EP1	
CDH6	ANXA1	NFIB	PDLIM4	APP	UVRAG	ECI1	NDUFA1	ORC3	ORC5	
KLK6	CDKN1C	TP53I3	SOX4	HMOX1	HEBP2	EIF2S3	CACNA2D2	MTMR6	PPP1CA	

GLDC	TAP1	LRRC17	WARS	PBX1	BAX	TROAP	IQGAP1	FMO1	TEP1
MSLN	HIST1H2AC	SSPN	TXN	LRRC6	NKG7	EBP	SRPK1	RPA3	CBFB
UCHL1	THBS1	GATM	F3	C3AR1	ARHGEF6	ATP5O	ADAMTS2	USP14	CTBP1
HOXB6	CAV1	RFC4	PLA2G4A	TCIRG1	ENTPD3	MGST2	SLC35A3	PPP2R1B	TTLL12
ISG15	BIK	VDR	EDN1	MVP	ANP32A	EHMT2	CDC42EP4	DLEU1	PPP1R7
APOA1	PROS1	PFKP	CD58	FAS	ALDH6A1	MLF2	MTR	TRAM1	TRAM2
CLDN10	FHL2	PTPRC	ATP2B2	SIRPA	TSPO	DDX21	AP2M1	SYPL1	CKAP5
FOLR1	ESR1	FUT8	ANXA4	GNAI1	YWHAH	PRIM1	ZNHIT3	GNLY	SLC1A1
IL8	CCND2	IL7R	GPR56	QSOX1	ERBB2	PRKCB	ABL1	GTF2E2	SEPT9
CCNA1	MYB	MFAP4	DAPK1	B3GALNT1	ETHE1	CD38	HNRNPH3	SLCO2A1	CHKA
VCAM1	IGF1	EPHX1	SORD	SLC19A2	ESPL1	ADAM17	BRD8	TMED9	ARL1
CP	MX2	EMP2	GZMB	ROR2	BAZ1A	TUBG1	ZNF22	ATP6AP1	PPP2R1A
COL3A1	SPARCL1	GZMA	GALE	AURKB	HOXC4	RALGPS1	OCLN	EIF3I	KCNQ1
S100A2	CXCL11	IGFBP6	HADH	NQO2	NFIL3	ABCD3	ATP6V0D1	SRSF9	MCC
SERPINF1	PRKAR2B	RUNX1	FYB	GCNT2	HOXB3	TOPBP1	SPTBN1	TRAK2	EXTL2
ERBB4	SMARCD3	MAF	AURKA	IRF8	RUVBL1	TGIF2	CCNH	SNRPD3	LSM1
COL10A1	ALDH1A3	TGFA	MARCKSL1	ENOSF1	EPHA2	ATP6V1B2	CFLAR	GCNT1	IGBP1
LAMA3	CX3CR1	FSTL1	LAMA2	STXBP1	NR3C2	PDHA1	NBR1	PSMF1	TSG101
FN1	MT1G	PFN2	FCGR2A	ANXA6	ZFP36L2	HDHD1	PPP3CA	KBTBD11	TGDS
MMP11	LAPTM5	ST3GAL1	LYN	SLC39A6	GPC1	FUT3	ZNF593	VAV1	CDC45
AEBP1	PODXL	ADRA2A	IL6ST	LIPA	RCBTB1	SKP2	PSMD10	PIGK	RDH5
COMP	PLOD2	RUNX1T1	LCP1	FERMT2	CEBPG	PRCC	CDK4	TTC3	FAAH
CCND1	SDC2	ALPL	PEX6	SCD	HSD11B2	VAMP8	KLHL21	ING3	RASA1
RGS1	DDIT4	IDH2	HK2	ACOX2	IL1B	ITGA5	RIN2	PKP4	TRIM13
HOXD3	GMPR	EZH2	BCL3	ATP2B4	GGCT	SSR4	KRT10	SMAD2	EIF2S1
IFI6	IER3	KCNN4	LAMA4	MCM3	RAB9A	CLK1	PPT1	PIP4K2A	CX3CL1
CTGF	DLGAP5	UBE2L6	AHR	SMAD7	MRPS2	DFNA5	CDK7	SOD3	PEX13
CFB	MUC1	KLF5	RASSF2	HLTF	RIPK2	ACTN4	RECQL	RRAGA	NAAA
ID1	CXCR4	PLSCR1	ITGB5	TRAF5	ACPP	ICA1	CNPY2	MYH14	DYNLT1
LOX	CFI	LGALS1	TJP2	IFNGR1	IPO5	SHROOM2	NUP160	SFXN3	TCF7
SERPINA1	HOXB7	CDC7	JAG1	SALL2	HDAC2	TCF15	ERN1	EXT2	RAB11FIP3
SERPINE2	EPS8	SIK1	PRKD1	PDXK	EPCAM	PSMD8	PSMA5	JAK1	ZFAND5

SCG5	CD74	FYN	CPM	PKIA	ACAT1	USP13	MAP2K1	DPAGT1	PLIN3
GATA6	GPRC5A	OSBPL3	CAPG	CASP6	SLC25A5	F12	AUH	HIVEP2	TJP1
BCAT1	PLIN2	RGS4	NR4A1	SCRN1	SPAG1	LRP1	PCNXL2	SPTBN2	IRF2
CDH11	CTSS	EVI2B	LMO2	IMPDH2	PIK3CD	HPRT1	NFE2	CDR2	BMPR2
ACTA2	HOXA5	VIM	NMI	TLR2	CYP2J2	BAZ2B	OSTF1	CYB561	MRPL23
FAP	PI3	PRKCA	CYTIP	TLE1	LPL	ST13	KIF13B	SLC35B1	KIAA0513
TSPAN8	ASS1	PCNA	PDGFRB	PGM1	SLCO2B1	GPR143	SOCS2	MAP3K8	DNAJA2
IDO1	DHCR24	GPR183	DHRS7	SLC5A1	PCK2	CCNC	KIAA0196	PRKAR1A	NMB
MMP9	FCER1G	SH3YL1	EFR3A	TSC22D1	LMOD1	F8	WNT2	STAT3	LYST
TNC	TP53	FOXJ1	EFNA1	FLNA	RRAD	DBN1	ZFYVE16	CDC16	NDUFV1
MX1	NCALD	CNN1	IRF1	MCM5	PCCA	DTNA	KCNJ8	GLUL	TXNRD1
CXCL2	TPX2	TPM1	PLXNB1	NR3C1	TRAF4	DCBLD2	FLI1	CCT5	NDUFB6
NMU	CENPF	HTR3A	CDH1	RCBTB2	PPP2CB	SEPHS2	MAP4	SPSB1	KIFAP3
CYP1B1	FRZB	KIF2C	CD36	P4HA1	CAT	CYB5R1	ITPR2	SEC23B	TANK
SERPINA5	CCL2	PRKX	NFKBIA	TAPBP	AHDC1	POR	CLCN3	ZMPSTE24	RAP1A
MMP2	COL6A1	SORL1	CTNNAL1	SLC11A2	FCGRT	LTA4H	ECH1	STARD13	ZFP36L1
CLU	COL16A1	MYO6	PGD	BMPR1B	ACTN1	ADAM19	HK1	DNAJB1	RAB6B
CTSK	WFDC2	TNFRSF11B	MN1	LMO4	TLR5	CREBBP	INPP5D	RNF6	HNRNPF
TSPAN7	RUNX3	PRSS8	HMMR	VBP1	TFDP1	TP53BP2	VAMP1	TSC2	RAB8A
S100A9	KLF4	LAMC2	CELSR2	TSPAN5	CD3D	CDKN1B	PRPF8	RNF14	GNB1
S100A1	COL4A1	SCNN1A	BTG2	PDIA5	SRGAP3	PMS1	SYNCRIP	SLC31A1	SLC4A2
ATP6V1B1	PRKCI	FBN2	JUN	RRAS	PKD2	SCP2	MTX2	RAB2A	SCARB1
PDGFRA	CPVL	VEGFA	CTSH	THBD	ST5	SMAD3	PSMD12	UROS	SLC4A3
CXCL12	GPRC5B	ODC1	OASL	JUP	RPS6KA3	CSF2RB	CAPZA2	ZNF140	AP2B1
CCL20	IGF1R	COL4A2	PRKACB	NCF2	PKN1	HSD17B6	IDH3A	GNB2	SSSCA1
LAMB1	FBLN5	RAPGEF3	CEACAM1	ACP1	MCM6	ATP5D	SMTN	DESI2	DLG1
HBB	GUCY1B3	GPC4	PRPS2	ANGPT2	RAD54L	PDK1	AKAP11	ZNF711	P2RX7
ID4	XAF1	IRS1	CYB5A	CD47	PPP1R3C	HLA-DOB	NME4	ETFB	HNRNPAB
GPNMB	HOXB2	MSX1	LGMN	COL18A1	ALDOC	GRN	HMGCS1	RBBP7	STXBP2
COL5A1	GBP2	TK1	SQLE	THRB	SMAD4	YES1	CDC25A	TAF11	MYL5
HMGA2	SOD2	IRF7	SLC39A14	ETFA	VEGFC	ABCA5	IBTK	RAE1	TAX1BP1
DUSP1	CD44	FGF13	JAM3	OXCT1	SSFA2	PSPH	ATP2A2	EPHB4	KCNN3

CYR61	NT5E	IL11RA	GFPT2	NFE2L3	GYG2	LEPR	CLDN5	ITGB6	TM9SF1
CRYAB	SERPINE1	APOD	P4HA2	IL18	MRE11A	C5AR1	FARP1	PPOX	GSTO1
GJA1	FMOD	CORO1A	ENPP1	TRIP6	GAA	HSPA14	PDHX	XRCC5	PRKAB1
TDO2	MYC	ISG20	NID1	CPD	ST14	ATP6V0B	SREBF1	MARCH6	LTBR
PPAP2C	FZD2	RCN2	CIRBP	SLBP	ANXA5	RNF144A	MAP2K6	SLC25A24	SMAD5
FOSB	SNCG	VLDLR	ITGAV	BARD1	PIK3CA	S100A11	ENG	ETF1	MYH9
ACTG2	MT1X	IL6R	POU2AF1	RBM3	INPP4B	GPC3	STX16	TAF12	NFKBIE
CRABP2	CKB	DSG2	DCHS1	BTC	TMX1	PEA15	TMED3	ZDHHC17	DHX15
S100A4	HSPA2	KCNMA1	H1F0	FEN1	TNFRSF14	GLS	KIF22	ITGB3BP	FNDC3A
FGFR3	FAT1	TGFBR2	NPR3	SCRIB	RFC5	HIVEP1	MYO1F	ELN	COPS2
INHBB	CD55	MLLT11	TWIST1	KRT15	ADD2	BLVRA	MCF2L	CHN2	ATP1A1
MGP	TPBG	ASNS	NCAM1	THBS3	CACNA1A	GLO1	TALDO1	PAFAH1B1	SLC25A11
GAS1	MEST	LPAR1	SERPINH1	THBS4	UCHL3	CSE1L	TM9SF2	PDCD6	ST3GAL4
CD200	SLC2A1	TGM1	EIF4EBP1	BAG1	ACTL6A	HIF1A	SRD5A1	CLIP1	TSC1
SFRP1	IL15	ARNT2	UPP1	TAP2	GCLC	CD164	VAMP7	MEF2A	RBM42
FGF9	CLDN3	MFHAS1	TFPI	AXL	NNT	SLC25A46	NDUFA5	TAF4	CDH18
GSTT1	IGFBP3	DHRS3	MAP3K5	CEBPA	CALB2	PSME4	WEE1	RNFT2	RTCA
TNFSF10	SLIT2	HMGA1	AQP5	GSR	YAP1	NRAS	WRB	EP300	HAT1
NPTX2	SPARC	BCL2	FZD5	TNFRSF1B	DNAJB6	PTK7	REST	LIG1	ATP5G3
FXYD3	CD69	SEMA3A	CYBA	WWP1	ADD3	UCK2	INPPL1	SNAP23	ATP5H
PLAT	ENPP2	FCGR2B	DAB2	AOC3	CD93	CPNE3	SMARCE1	BMP4	GM2A
RGS2	ALOX5	ACP5	CD99	ZNF185	TBX2	HEXB	MTIF2	SLC16A2	MAP3K4
HIST1H1C	COL4A5	TXNIP	LAPTM4B	PTGER2	NDUFS4	AES	PKIG	TXLNA	PSMC6
ADM	MFGE8	ELF3	ELF4	GCHFR	PTPN3	RGL2	STAT6	FDXR	CSTF1
NID2	PLA2G16	NUCB2	RALBP1	C1QBP	TCF7L2	IL15RA	TIE1	IARS	MARCO
CRIP1	CPE	GUCY1A3	GPR137B	ZMIZ1	DNAJB9	ETS2	TMEM147	PSMD6	CAPNS1
ISLR	LOXL2	RAP1GAP	LAMA5	S100P	APLP1	PALM	ICT1	PSMD13	CEP164
RARRES3	MLF1	MCM2	ZNF165	SMPDL3A	PIK3CG	STIP1	PTS	RPGR	FLNC
CXCL9	ETV5	CCNA2	BDH1	HRSP12	PPP2R2B	AKAP1	PSMB5	HSD17B4	EIF4G1
SLPI	BMP7	SLC7A5	CDK6	CAPN2	MOCS1	AHCY	RFX3	AKAP17A	CEP57
CDH2	PLS3	HES1	GPSM2	DHRS11	SNTB1	ARHGAP5	SLC35A2	NPC1	GANAB
COL6A2	STAT1	PSMB8	EZR	GPD1L	HSPH1	CCDC85B	NUP153	CNTN1	RFC2

IFI44	MYH11	TRIP13	PDIA4	PAICS	CAST	NUP155	SH3PXD2A	NR1H3	PSEN1
BST2	BIRC5	FBLN2	AKT3	SCARB2	HERPUD1	PFDN4	FAM50A	NCOR2	CAPN6
ATF3	BTG3	MTHFD2	STOM	GRB10	TMPO	IDH1	ANKRD17	SKP1	RNF4
COL6A3	NEDD9	KRT14	MAMLD1	HLA-DOA	KCNJ2	NINL	PAK2	ENTPD1	GTF2H1
SULT1C2	QPCT	UCP2	PLD3	GABRP	FPR1	PSMB2	PDK3	RAB11A	CAPN3
IGFBP2	RNASE1	GNG11	HSD11B1	FKBP5	NCKAP1L	RPA1	FBXL5	ESYT1	N4BP2L2
SNAI2	SGK1	RND3	ATP2B1	NELL2	HIBCH	TARBP1	BPGM	YWHAE	PAXIP1
PTGS1	CD53	PLS1	TKT	CORO2A	SMARCA4	ABR	AGPAT2	MLEC	DDX1
WT1	CA8	AQP9	SMAD1	SLC35F2	SOCS3	PABPC4	DBI	CHERP	HPCAL1
C1S	TRIM22	NCAPD2	PLA2G7	DDX39A	BST1	BCR	OGT	SUZ12	CFDP1
C1QB	TNFAIP6	CFD	RFC3	PRKCD	SLC39A7	PSMD14	TBPL1	PNPLA6	TCF12
PLAU	ECM2	SDC4	SERPINI1	MSN	LAMP2	ENO1	NRP2	GPX4	SHC1
RBP1	ITGB4	ADAM9	ELK3	NAIP	FOSL2	WSB2	TUBGCP3	TLR1	TSC22D2
GBP1	CAV2	RGS5	COX7A1	TOB1	ASPH	ARL3	TRIM38	PLK1	KIAA0100
LOXL1	G0S2	EPAS1	LCP2	TGFB1	SLA	STAB1	MFSD10	CSNK2A1	PAX6
TIMP3	ALDH2	GEM	CST6	GPR37	KLF9	FLNB	FRK	ALDH3A2	SHMT1
SOX9	TYMS	PLAUR	SPINT2	IRAK1	ZNF266	PRKCH	GNA15	TENC1	PSMB3
RARRES2	SLC6A8	ST3GAL6	MSMO1	SERPINB6	BTN3A1	AKT1	POLD2	TDG	ACO1
HLA-DPB1	TGFBR3	MEIS1	CSRP1	CDC25B	NME3	DMD	PPP2R2A	NARS	PMM1
IFI27	RHOB	CDR2L	ABCG1	EPHB6	NDUFB7	CETN2	LRPPRC	UQCRC1	CAP1
GDF15	KLF6	RYR1	AQP1	TPD52	LAGE3	CENPE	EED	CD81	ORC2
TRIM29	XK	RAB40B	GRIA2	STMN1	DZIP1	PRKCQ	PSMC5	EIF4G3	SRRM2
CCL5	DUSP6	EBAG9	DNMT1	GATA3	CD46	HSPG2	KIAA0247	HMGN4	UGP2
APOE	FZD7	GJB1	TNNC1	ASAH1	PRKDC	LGALS8	ZNF45	PDK4	PPP2R5A
SPOCK2	FST	KCNK1	PBX3	PAFAH1B3	GABBR1	GBE1	TMEM187	DPP4	USO1
NQO1	SMARCA2	STIL	TSTA3	WIPF1	ALG13	COX7B	ADK	UCHL5	CDK2AP1
SEMA3C	FBXO21	CDK14	MMP12	CKMT2	CDK2	TSPAN3	PIM1	POLR2F	DIS3
SPRY2	AGR2	NR2F2	CCNG1	EIF2AK2	SMC1A	NUP210	GGCX	FARSA	IDH3B
GREB1	IFI16	TCF4	CA9	FRY	CCNG2	CASP7	GPD2	CHMP2A	DAD1
FILIP1L	CD14	CA2	PTPRK	PTPRF	LMNA	CLNS1A	CD4	CCT6A	PTPN12
NDN	MT2A	BCL6	IL1RAP	FEZ1	BIN1	CCR5	UBE3C	PLCG2	IRX5
ALDH1A1	IL1R1	RTN1	BCL2A1	VRK1	AMT	EXO1	POLD4	COL7A1	LRRC8B

PTX3	CCL8	CKS2	FBP1	RB1	RRM1	SEPT6	PCGF2	HSF2	MYD88
KRT7	TTK	TFRC	LRRN2	MFN1	BLVRB	HSD17B8	SLC12A2	SS18	TRRAP
HLA-DPA1	RNASE6	TRO	NAB1	PNPLA4	TMC6	LPIN1	PHKB	CDK9	RUNX2
NUAK1	ALOX5AP	EDNRA	FOLR3	SYT11	DUSP4	NFIC	MLLT3	FEM1B	MRPL19
CCNE1	ZFP36	ABHD3	KIF23	ABCC3	TRIM28	DLAT	PAEP	CD247	GZMH
IGFBP4	NETO2	HMGB2	GRB7	SHMT2	PDCD10	SRI	RAB21	PPP2R5C	CXCL3
ST6GALNAC2	TPD52L1	CCDC6	LBR	PCBD1	PIK3CB	VILL	PURA	STK38	LASP1
TFAP2C	SELENBP1	MST1R	LITAF	MSH6	ITPR1	GPAA1	NOP2	GOLGA2	SRSF5
CXCL1	TM4SF1	FOXO1	ABLIM1	ATP7B	APLP2	MGMT	GUSB	ALG8	PTPRB
NR4A2	MMD	PDE9A	RGS16	PCCB	SNRPB	ZYX	MAP4K1	BECN1	OLR1
SPON1	TUSC3	NPAS2	STC1	ME1	GRINA	BRD3	ZNF7	CTNNB1	VPS45
ZNF423	SATB1	SMARCA1	LAMC1	SELL	SEC14L1	GCA	SLK	HINT1	IRF3
SFN	KDELR3	LSR	LIMK2	SLC29A2	PCM1	SLC20A1	FXR1	SDS	COX6B1
PMP22	CD52	GPR161	BSG	FMO2	IL13RA1	NAP1L1	POLE2	RAD23A	CASP10
LY6E	ID3	IFI35	PLAG1	MYBL2	INSR	TIMM17A	FAM120A	AKAP7	STK25
CBS	AIM1	KANK1	PYGL	GALNT1	SMARCC1	PDCD2	ATRN	SPTAN1	PPRC1
TNFAIP2	WNT7A	CD2	SCPEP1	MMP1	GALC	NUP205	LPP	CAMKK2	GLG1
CD163	ATP1B1	NOTCH3	CFTR	COL9A3	PTGS2	EBNA1BP2	GLB1	CALCOCO2	VAMP3
SDC1	SLC16A1	JUNB	TST	CLIC4	SMPDL3B	NR4A3	TNF	PRDX3	PSMC2
KAL1	TSPAN13	TPM4	SYK	ITGA6	NUP88	RORA	DUT	RHOC	ZNF529
SPP1	KIAA0101	PFKFB3	USP1	SLC6A12	FUCA1	AVL9	TMEM59	NDUFA2	PCMT1
C1R	HNMT	GSN	CENPA	MAP4K4	DUSP2	P4HB	PLEK	POLR2H	FAM193A
MATN2	ARHGDIB	ANK3	PDLIM5	PDE4A	STT3A	PLEC	DVL1	ARID1A	STRN3
AKAP12	MAD2L1	IL10RA	DCLK1	IL1RN	IGF2R	GOT1	IL10RB	DFFA	FGFBP1
THY1	NDRG1	AIFM1	FKBP4	SKAP1	RDX	E2F3	SSB	ROCK2	ARHGEF7
COL15A1	FGL2	MKI67	SAT1	AZIN1	PROCR	PTDSS1	ADA	CAD	LANCL1
FBLN1	CNN3	PNP	PDE4DIP	ARHGAP32	NCK1	ILVBL	GLRB	SLC2A5	FCHSD2
BIRC3	ST6GAL1	CTSC	KIF1B	PRDX4	MBP	FAM189A2	POLR2L	F2R	MPP6
GABRE	SLC7A11	CSF1R	OAT	ASL	PPFIBP1	LAD1	COL5A3	CHP1	EMC1
GSTM3	PLTP	SLC15A2	MAGI2	TPST2	CHD1	PON3	USP11	PFKL	ATOX1
EFS	CDK1	CDKN1A	DDR2	SEC23A	DPM1	ICAM3	CDC25C	NDUFS7	AIMP2
OAS2	F2RL1	SYT17	ERMP1	ZBTB16	EPB41L3	COX6C	FOXN3	CDO1	TPR

MEOX1	TNFAIP3	ITPR3	STK3	KIT	CD8A	MAPK1	SMG7	CCT3	FNTA
NPR1	PDE4B	PSIP1	TRIM14	KANK2	ARF3	ZNF239	DECR1	TFAM	NUCB1
GPM6B	EGR3	HOXB5	CD9	CBLB	NT5C2	TES	DCK	RPA2	ECHS1
COL1A2	CCNB1	PCSK6	TNFSF4	ITGA7	ABCA3	NTF3	PRMT1	BLM	USP48
AR	SRPX2	NRIP1	TCEA2	CCL11	HSF1	ATM	MPP1	PTGER4	TRIP12
HIST2H2BE	LTBP2	LIF	CCL18	CTSD	ANKRD46	COL14A1	MAPRE2	PHF3	SERTAD2
IFIT3	EMP3	EPHA4	PTCH1	STAR	KLC1	NEO1	EPRS	AHCYL1	SLC25A4
OAS1	CELF2	FDFT1	TCFL5	ATF2	TARS	VCL	INPP5A	PVRL2	RBM25
BGN	CYC1	CLDN7	PON2	ERO1L	CCNO	WSB1	RAD51	AFG3L2	HTATSF1
FHL1	MPZL2	TIMP1	CD97	EPHX2	IDS	PPP1R16B	ACVR1B	CYB5R3	SEL1L
COL9A2	MYO10	WASF1	GHR	OSMR	EGFR	METAP2	HSPA4	MAD1L1	TAF5
PHLDA2	PIK3R3								

Supplementary Table 6

Genes

SCGB2A2	NELL2	DHRS2	PSCA
TFAP2B	C8orf4	FGFR2	NOVA1
SCGB1D2	ANXA3	LYZ	ATP6V0A4
LTF	PTGER3	SGCE	CPB1
PIP	MMP9	TRIP13	DIO1
S100P	IFI44	KCNK1	ELF5
NPY1R	KRT7	CD2	MMP1
TFF3	GGH	CCL5	PTPRT
CXCL13	GALNT6	RHOBTB3	MMP13
PEG10	PDE4B	MGP	LRP2
SCUBE2	CKMT1B	C10orf116	GABRP
KRT15	TOP2A	CFD	COL1A2
CXCL9	SAA1	ASPM	S100A2
CEACAM6	TMPRSS3	AZGP1	SEMA3C
UBD	HOXC10	GREB1	MRPS30
TFF1	SYNM	LRRC15	CXCL11
KRT23	PPP1R3C	HBA1	GFRA1
SCGB2A1	GRP	PHLDA2	ERBB4
PROM1	ID4	CRYAB	S100B
FABP4	CA12	CRIP1	RTN1
AQP3	COL14A1	TBC1D9	CYP2B6
APOD	ASS1	EFEMP1	CYP2B7P1
S100A8	ASPN	KRT6B	HBB
VTCN1	IGHM	ACTG2	EN1
IGKV3D-15	PTPRC	CLU	NR2F2
STC2	PLAT	GJA1	BMPR1B
IGKC	ERAP2	AURKA	SOX10
SLPI	IFI27	STAT1	GNAZ
CA2	AREG	STC1	FABP7
ALDH3B2	GALNT3	PMAIP1	POSTN
BAMBI	MMP7	PSPH	CHD2
HLA-DQA1	RBP1	VAV3	EMP1
S100A9	ANXA9	NTRK2	HTATSF1
COL11A1	ZNF238	G0S2	SPDEF
TMC5	CD36	IGFBP2	S100A7
MUC1	IGFBP5	RND3	EPHA4
AGR2	CSTA	PRC1	PALB2
ABAT	COMP	CAMK2N1	HTR2B
CHI3L1	HSPA2	PSD3	ZCWPW1
FGFR3	EIF5A	REEP1	C18orf25
DNAJC12	S100A14	SCNN1A	CYP2W1
GSTM3	CD52	FAM129A	ADIPOQ
DUSP4	CX3CR1	C1orf115	SNRNP200
CXCL10	NFIB	SRGN	TM2D1
HLA-DQB1	COL10A1	COL1A1	CRABP1
SPP1	EVL	DCN	PSMB7
MAOB	TM4SF1	GATA3	SLC52A1
IFIT1	LPL	AKR1C2	U2SURP
ADH1B	MYBL1	SYBU	TAF11
CXCL14	CILP	BST2	JRKL
TOX3	ARNT2	CYP1B1	PTGER1
EHF	HIST1H1C	GBP1	COL6A2
CFB	PDZK1IP1	CD24	CST1
LRRC17	PCSK6	HOXB2	BUB3
SERPINA3	NQO1	AKR1C3	TRMT1
IGJ	HLA-DRB4	NEK2	SUGP1
SERPINA5	PAPSS2	CELSR1	CEACAM5
SLC1A1	SERPINA1	GRIA2	AHSA1
MX1	HIST2H2AA4	PDZK1	PSMA6
C4A	RARRES1	HMGCS2	PEG3
CLDN3	TGFBR3	SYT13	KCNE4
WFDC2	THBS4	SLC7A2	CDC27
TSPYL5	OAS1	AGTR1	PPM1F
IFI6	CCL19	NDP	CYP24A1
ISG15	CLGN	CNTNAP2	HBG1
IFI44L	HIST2H2BE	SYT1	KCNAB1

Supplementary Table 7

Genes

SCGB2A2	FASN	CDH11	MBP	CYP51A1	C8orf33	PMP22	WRB	TSPAN15	DDAH1
TFAP2B	RARRES3	INHBA	PRNP	RBMS1	EPRS	CXCR7	PTP4A2	AQP1	ZNF552
SCGB1D2	CRABP2	SLC2A10	SLC6A8	PON2	TUBB6	ZBTB20	FANCI	KLHL24	ZYX
LTF	SQLE	MEGF9	HERC5	TMX1	LCP1	ACOT7	ELL3	GAS6	RAB1A
PIP	NUSAP1	WLS	ZNF652	RNASE1	HEPH	IRF8	HEY1	AUH	TSKU
S100P	PDCD4	ATF3	YWHAZ	SLC5A6	FUT8	NME7	BCL6	SEMA3G	CLK4
NPY1R	IL7R	LY75	DCXR	KCNN4	FGFR1	PAM	ATRX	ARL4A	AGAP1
TFF3	FBP1	UBE2E3	MEST	KANK1	TPBG	NOX4	CIRBP	LYPD3	SRI
CXCL13	CYP2B6	CXCR4	PIK3R1	ASNS	RFTN1	BUB1	FMOD	TP53TG1	LRRFIP1
PEG10	NEAT1	MYL9	DEPTOR	CYFIP2	ALDH2	OAT	MT1H	METTL18	SAC3D1
SCUBE2	TPD52L1	APOBEC3B	CAV1	EPB41L4B	S100A13	LPXN	UBXN4	MARCH2	CUL4B
KRT15	ENPP2	COL5A2	CTSD	RABEP1	FKBP4	LIMK2	IL1R1	CSNK1A1	VDAC1
CXCL9	LIMCH1	LY96	CNN3	CD302	ATP6V0E2	DNAJC15	SLIT2	PDIA6	SNRNP70
CEACAM6	AMIGO2	DHCR7	RUNX3	EEF1A2	TMEM47	TCF7L1	CAT	GSPT1	COX11
UBD	BACE2	ITGB4	RGS1	GDF15	PGRMC1	PRKAR1A	MTMR2	PDXK	SLC27A3
TFF1	FHL1	MAOA	HIST3H2A	EVI2B	PECAM1	AGPAT5	EMP3	MXRA7	IL2RG
KRT23	C6orf211	IFI16	AKR7A3	PYGL	PLAC8	ELN	CDV3	RAB11A	SERP1
SCGB2A1	COL8A2	HIST1H2BG	FCGR3A	MTUS1	GBP2	PTGES	CYB5R1	CD9	LEPROTL1
PROM1	SH3BGRL	DDX58	UBE2S	GMNN	SPR	STEAP3	ITFG1	MXI1	FANCL
FABP4	AKR1C1	BTG2	AKAP12	SRPX	BSPRY	SPTSSA	RUFY3	LGALS9	MORF4L1
AQP3	RAI2	ARL4C	FZD7	PDZRN3	ZMYND8	SMYD3	COPS8	CDKN2A	ST14
APOD	CD3D	COX6C	SREBF1	PPIC	N4BP2L1	PFKM	NAA15	TPGS2	ANKRD10
S100A8	TUSC3	ADAM12	AKAP9	C9orf91	NEDD4L	TACC1	C15orf63	CEP350	ENDOD1
VTCN1	METRN	RAB31	KCNMA1	APLP2	GMFG	ACACB	RAD51AP1	ABLIM3	SLC29A1
IGKV3D-15	TNFSF10	KRT8	SLC39A4	ZNF395	GOLPH3L	HRSP12	ARF1	ASRGL1	CTSF
STC2	CADM1	APOE	LAGE3	ARHGAP8	ITPR2	CKS1B	AGPS	RASSF2	LAP3
IGKC	ACOX2	NNMT	ABCD3	KRT18	STK17A	FOXM1	RAP2C	CTSO	ADCY9

SLPI	ERBB4	IQGAP2	LTBP1	TM9SF1	MCL1	CPNE3	QPRT	SKP2	SMAD3
CA2	SYT17	HLA-DMA	CXCL12	ANO1	GALC	MAPK13	MRPS28	SNX4	ARHGEF10
ALDH3B2	FHL2	SRSF6	EFHD1	OSBPL1A	NDRG2	DPY19L4	SIK3	GPI	IDH1
BAMBI	HSPB8	SORBS1	NUCB2	THBS1	PPIF	CREBL2	MAP9	IMPDH2	OXCT1
HLA-DQA1	UGCG	DHCR24	APOBEC3G	IFI30	DBN1	TMEM251	SYNE2	CACYBP	SEC23B
S100A9	SEMA3C	MGLL	PPM1H	RAB25	TGFB3	FADS2	LAMB1	SMCHD1	CTSL1
COL11A1	ATP2A3	F12	SLC27A2	CD53	NGFRAP1	SVIL	ITPR3	ALOX5AP	MGST3
TMC5	LAPTM4B	CHPT1	CHKB	DUSP10	ARHGEF6	CDC42	GALE	DHRS7	TCTA
MUC1	RAB11FIP1	KCNE4	GPM6B	ABCA8	TRIM29	MS4A4A	PCCB	ZFAND1	FGFR1OP
AGR2	AR	NETO2	CYTIP	HNMT	STOM	GLT8D2	ALG13	PDIA4	SRP72
ABAT	ATHL1	C1S	HSPA1A	KIAA0040	PSMD12	RNASET2	HMMR	RABAC1	SAMD9
CHI3L1	IRS1	TCF7L2	OSR2	MXRA5	BLVRB	MET	CCND2	SECISBP2L	ARGLU1
FGFR3	ADM	HMGB3	HN1	WWP1	CDC42EP3	MIR22HG	PION	PCSK5	AIDA
DNAJC12	KYNU	ITGA6	C1QB	IL32	CEACAM5	SH3YL1	AHNAK	SEC23A	SRSF7
GSTM3	CTSS	TMEM176B	HLA-DPA1	SPATS2L	CAV2	FUCA1	WWC1	RABGAP1L	TBL1XR1
DUSP4	ASPH	DPT	DENND1B	NUDT21	BLNK	PCBP2	GMFB	TYMP	NXT2
CXCL10	GZMA	ANXA1	PLS3	SLC38A1	ABCA3	ETNK1	CALU	SMC3	DONSON
HLA-DQB1	LTB	DUSP5	IFIH1	CBR3	CCL4	DNAJB6	PKP4	TMX4	MAT2A
SPP1	CCL8	MX2	CEACAM1	PSIP1	FCER1G	ANKHD1-EIF4	RBM25	TMEM208	TSC22D3
MAOB	TTC39A	ODC1	EZH2	CEBPD	ID2	CDK19	SPEN	RNASE6	DNAJC10
IFIT1	GREM1	TXNIP	RPS21	CITED2	GPR56	TCEAL1	CCDC47	WSB1	CTTN
ADH1B	SLC7A5	TNFRSF21	CASP1	HILPDA	MEIS3P1	PPAT	ELF1	CDC42EP4	PRKCH
CXCL14	PSMB8	SPON2	CLEC2B	BTN3A3	TMEM30B	GGT1	PDGFRA	RBM8A	SPA17
TOX3	CYBRD1	GABRP	BTN3A2	SLAMF8	TNNT1	CHI3L2	MSH2	FAM176B	NMRK1
EHF	TMEM45A	ARMCX2	CDKN1C	APP	LAMP1	TIA1	SLC7A1	SSBP2	MRPS31
CFB	GSTP1	DUSP6	LRBA	RGCC	CPB1	RAD51C	MAP4K5	SPOP	KIAA0907
LRRC17	HBB	BEX4	TBL1X	BHLHE41	SOCS2	MAP7	SEPT10	EXOSC4	EIF4E
SERPINA3	FAM134B	PALLD	BGN	LYPLA1	TLE2	GAPDH	TJP2	CHD9	EFNA4
IGJ	GSTT1	GSTA4	SERPINF1	ZC2HC1A	TK1	FBLN5	PPP1CB	TNFAIP2	MTPAP
SERPINA5	ELF5	NR4A2	HLA-DRA	HLA-A	PTPRF	WFS1	KPNA3	PAICS	HS2ST1

SLC1A1	HIST1H2AC	RCAN1	SNRPN	CRYZ	ZFP36L2	RPL39L	MED13L	TAF1D	GPX7
MX1	CXADR	FERMT2	EPAS1	DKK3	ME2	FUS	ID1	SPINT1	EIF2AK2
C4A	SLC19A2	SLC39A8	MT1F	PROS1	TMEM132A	GFPT1	BIRC3	FOXN3	MACF1
CLDN3	VCAN	OAS3	GLI3	TNS1	SDC4	MAFB	SETBP1	RBCK1	H3F3A
WFDC2	FZD6	PPL	C10orf10	RTN1	PTPLAD1	SMARCA2	LGALS1	ACP6	TBC1D8
TSPYL5	MYB	GZMK	XIST	PEX11A	TOB1	NID2	LPGAT1	RPA1	CCT2
IFI6	CYR61	POU2AF1	WISP2	HLA-G	TMSB15A	ZWILCH	RCHY1	EMP2	CRNKL1
ISG15	SLC24A3	MMP2	HLA-DPB1	USP1	AKAP2	NME3	COPZ2	SMARCD3	BMI1
IFI44L	EGR3	TAP1	TNFAIP3	TRIB3	HOMER1	HIGD1A	AMD1	PLA2G12A	LMCD1
NELL2	OAS2	KIF5C	SAMSN1	PLEKHF2	UPF3A	BSG	RUNX1	BOLA2	PTDSS1
C8orf4	SFRP4	ISG20	ANPEP	IGFBP4	GM2A	PREPL	HADH	POGK	FOLR1
ANXA3	ADIPOQ	AK4	PFN2	AP2B1	PLEKHB1	CKAP4	CAPG	UBA7	NAPA
PTGER3	DARC	LAPTM5	JAG1	PALMD	GNG12	TSPAN31	PMVK	HLA-DMB	CDKN1A
MMP9	DACH1	GOLGA8A	GPRC5B	PBX1	RAD21	DESI2	CFH	FNBP1L	PQBP1
IFI44	CCND1	ECM2	MICB	TPM2	UCP2	HSPD1	TAPBPL	UQCRC2	RABEP2
KRT7	EGR1	LY6E	CSDA	TRIL	DDX3X	DIEXF	RBM47	BOP1	PLGRKT
GGH	MATN2	DTL	RMND1	MCM4	ABHD11	WIPI1	SCPEP1	LCK	WDR11
GALNT6	FN1	PRKAR2B	SMC4	HLA-C	CORO1A	TNFAIP8	SRPX2	CLDN7	C6orf120
PDE4B	MRPS30	HTRA1	FOS	NUDT4	LGR4	CERS2	HSP90AA1	SDHC	AKAP1
CKMT1B	DST	GPR137B	VCAM1	GSN	LZTFL1	FAM208B	SEPHS2	PAFAH1B3	LUC7L3
TOP2A	IL17RB	ACTA2	CD74	S100A6	SYNCRIP	TIMP1	MLEC	PXMP4	ZNF302
SAA1	BUB1B	SPOCK1	LGALS3BP	MCM2	RAB2A	GGPS1	IGSF3	MTHFD2	SYNGR2
TMPRSS3	FABP5	TMEM97	ALDH3A2	TUBB2A	GADD45A	HLA-DRB1	AGRN	EMCN	TBCE
HOXC10	SCD	LDHB	TLE1	MYO5C	ATXN1	SYPL1	LGALS3	PCGF2	MMP1
SYNM	RSAD2	ACP5	RHOB	EFS	WIPF1	SELL	PPAP2C	PGRMC2	ARHGAP5
PPP1R3C	AMFR	GYG2	PLIN2	NPDC1	FAM63A	CASD1	ZEB1	DHRS3	ABHD10
GRP	SMARCA1	ALCAM	C1R	METTL7A	TNFRSF12A	GMDS	STT3A	SGCB	VEZF1
ID4	PERP	KDM4B	TPM1	SRRM2	TRAM1	STIP1	MIS18A	FBXO11	PSME3
CA12	SOX9	KIAA1324	RBBP8	DBNDD2	PLAUR	LBR	ST3GAL1	DHPS	LARP1
COL14A1	SLC7A8	CCNB2	PDGFRL	COL4A2	PELI1	SNX10	PLP2	PLSCR4	NKTR

ASS1	ITM2A	ECM1	TRAF5	LUM	ISYNA1	SLC30A1	KIAA0182	PDLIM2	TCP1
ASPN	APOC1	POSTN	DEGS1	NBN	ANGPTL2	BTG1	DENND2D	ME3	SSR1
IGHM	SPON1	ADAM9	TIMP2	MLF1	CXCL2	NEU1	TOB2	FAM82B	TSPYL1
PTPRC	OLFM1	INHBB	FARP1	MAN1A1	C16orf45	GIMAP6	NOTCH2	S100A1	TMED2
PLAT	SCCPDH	SERPINE2	FAS	MAST4	DNAJC1	TWIST1	EPCAM	RNPEP	ZNF177
ERAP2	PSMB9	FAM20B	PFKP	ADAMTS5	KPNA2	LTBP2	KIF16B	RFC4	TOP1
IFI27	SEPT6	MDK	CD47	RECK	GIMAP5	CREB3L1	PRRX1	VSIG4	TUBB3
AREG	ENPP1	CTSK	CLMN	MBNL2	ERAP1	SH3BP4	MEIS2	RPL10	STMN1
GALNT3	H2BFS	AOC3	CD163	MRPL13	SSX2IP	TMEM230	PLCB4	PNN	TMED10
MMP7	CSRP2	CYP4B1	BIK	FRZB	COL18A1	EVI2A	SSFA2	ITGB3BP	SEC16A
RBP1	CALD1	CCR1	LCP2	PPP1R1A	COL16A1	PDE4DIP	FEN1	UBE2J1	RAP1GDS1
ANXA9	TSPAN6	SLC1A4	SCD5	DCTD	ACADSB	ELOVL5	APEH	WTAP	GCOM1
ZNF238	PYCARD	LEPR	CKB	BNIP3	ICAM2	FLNB	PDS5B	PRIM1	PRPS1
CD36	SLC44A4	LMO4	FGL2	MCAM	HMHA1	GEM	HNRNPD	PNISR	APTX
IGFBP5	SDC1	COLEC12	QSOX1	TOM1L1	CELSR2	HLA-F	MANSC1	C1QTNF3	WDR61
CSTA	BCL2A1	SRD5A1	IRF1	PIK3R3	KIF4A	BNIP3L	AACS	ANGEL2	GLG1
COMP	IRS2	MFAP5	CST3	ACOT2	FBLN2	TM2D1	SLC25A32	STAT3	FKBP1B
HSPA2	TNC	RAC2	PTGIS	C11orf75	SEZ6L2	IL2RB	ERMP1	PKM	WDR1
EIF5A	COL4A5	PLK2	NRIP1	HSPA13	NDN	DHX9	SLC5A3	PIGF	KTN1
S100A14	DDIT4	KCNS3	KIF13B	LONP2	ATP6V1C1	C1QBP	CPM	UBE2G1	REEP5
CD52	BHLHE40	LEF1	CLIC4	KDELR3	TCEAL4	GUCY1B3	SEC61A1	TXNRD1	CNIH4
CX3CR1	ALDH1A1	IER3	DCAF10	IFIT3	PHLDA1	DPYSL3	MRPL15	MYO1B	SAMHD1
NFIB	SPAG16	TFRC	SLA	ZNF22	FUBP1	F2RL1	PRKCB	LAMC1	URI1
COL10A1	GHR	NET1	LDLR	CD46	SKAP1	MREG	MARCH8	PPP1R14B	CNPY2
EVL	SORD	ITPR1	KDM5B	IFRD1	TNFRSF1B	HMGA1	DNAJB9	CREB1	ALDH7A1
TM4SF1	AGTR1	SELENBP1	MTSS1	ADAMDEC1	ATP8B1	SH3BP5	TES	ECI1	CCL3L3
LPL	CCNG2	FBN1	XBP1	ENAH	BASP1	TNIK	ADI1	WBP11	CANX
MYBL1	MAD2L1	INPP4B	JUP	MAN2A1	MSR1	ABLIM1	CSRP1	SFPQ	FAM115A
CILP	GOLM1	CELF2	HRASLS2	FLNA	PRKDC	AKR7A2	OSBPL8	CORT	MARCH5
ARNT2	CLSTN2	PDLIM3	KAL1	TRPS1	NR2F1	ENTPD1	ATP6V1H	TRIM37	BCAS1

HIST1H1C	KIT	ITGB5	SERPING1	SAP30	AGA	RUNX1T1	GLUD1	CTSB	HMGCR
PDZK1IP1	C3	CTSC	TFAP2A	SP110	CD164	FBXO21	CRISPLD2	KLRK1	JHDM1D
PCSK6	FXYD3	NFIL3	PDGFC	IFT122	SLC22A18	ZNF185	SLC31A2	RBM5	FBXO28
NQO1	FGF13	IFITM1	VIM	C6orf62	ARHGAP29	SGK1	PCMTD2	HN1L	MPZL2
HLA-DRB4	HSPB1	GAS1	EMP1	SLC16A6	NAV2	KCTD9	TP53	SF1	GAS2L1
PAPSS2	IGF1R	LAMA2	GTF2H2B	S100A4	PCOLCE	HSP90B1	GCLC	C2CD2	SP100
SERPINA1	QDPR	HLA-B	CYBA	HOPX	MED21	POLR1B	WNK1	C6orf108	SMYD2
HIST2H2AA4	HIST1H2BH	NDRG1	TRAF3IP3	WBP5	ZCCHC24	SLMO2	ECHDC1	BBX	VWF
RARRES1	FAIM3	BTG3	CEP57	PAWR	HES1	SELPLG	MED13	PCMT1	HIBCH
TGFBR3	NEBL	TRIM2	PLSCR1	TMPO	PPID	HSPA6	SLC33A1	MSH6	EPHB4
THBS4	MNDA	THBS2	ITGB2	SCRN1	LDOC1	TGFBR2	FBXO3	TGIF1	ARF4
OAS1	PLOD2	COBL	CCNE2	NME1	PDE4A	N4BP2L2	MINA	TLR5	ATP2A2
CCL19	GINS1	EIF2S3	ABCC5	GATA2	ACTN1	TJP3	SPATA20	FOLR2	GNG11
CLGN	PLA2G16	FYB	CDKN3	P4HTM	ENOSF1	DDX17	KDELR2	HIST1H4J	LHFP
HIST2H2BE	COL6A2	GUCY1A3	CYB5A	TIAM1	DSP	TMEM135	YBX1	AKT3	ICA1
DHRS2	GINS2	MLF1IP	SUB1	RDX	SMARCA4	CDC25B	PEX2	KIAA0196	TMEM204
FGFR2	UGDH	TPSB2	PLBD1	MXRA8	TWF1	MT2A	PCBD1	DHFR	NUCB1
LYZ	PEG3	LYN	SPDEF	COTL1	DDX60	THBD	TFPI	C14orf1	TSPAN12
SGCE	TAGLN	PRR15L	PDGFD	FDFT1	TPM4	ERBB3	TUFT1	COL4A1	PAK2
TRIP13	OPN3	TSPAN1	TGFBI	MAP1B	INSR	EPS8	MRPS14	IL10RA	SYNJ2
KCNK1	MYLK	MARCKS	GATM	CD8A	AKR1B1	SLC2A3	TTK	EPPK1	PPP2CB
CD2	COL1A2	SPARCL1	ZNF91	TACSTD2	ANKRD46	HCK	PTEN	TNFRSF10B	CDCP1
CCL5	MMP3	WWTR1	IL1RN	LSR	THEMIS2	MYCBP2	PIR	ZNF24	PHKB
RHOBTB3	ZDHHC11	HERC6	FBXL7	KCTD12	CENPN	RAMP1	BDH2	AZIN1	EFHC1
MGP	GPX3	PRSS8	IFI35	IFNGR1	RASA1	P4HB	EFEMP2	SAR1A	NUTF2
C10orf116	FMO2	MB	FYN	MICAL2	CYB561	QKI	SEMA3F	SEH1L	SMARCC1
CFD	SYCP2	PFKFB3	EZR	HSPH1	BUB3	RPRD1A	RRM1	TAGLN2	FAM13A
ASPM	GALNT7	ADD3	IRF6	PILRB	CD99	LAMA3	TRIB1	RAB27A	VPS13C
AZGP1	SLC52A2	SLC9A3R1	C14orf45	DPYSL2	DLG5	SEMA5A	POLR3K	CDKN1B	MSN
GREB1	MAGED2	HCP5	ARHGAP32	ENPP4	VDAC3	MAF	PTP4A1	SAP18	CD58

LRRC15	IMPA2	FNDC3B	FAP	HLA-E	PTPRO	TNFAIP6	DSG2	PARP12	TBC1D16
HBA1	FAT1	LGALS8	ZWINT	B4GALT5	RARRES2	RHOH	IL13RA1	H2AFV	RGS16
PHLDA2	TPD52	ADRA2A	GRB10	AMMECR1	CADPS2	EIF4EBP1	CERS4	SLC35F2	PNP
CRYAB	MTFR1	KCTD3	GLUL	C1orf63	ECI2	DDAH2	S100A10	EXOSC8	RAB40B
CRIP1	IL6ST	CARD10	LOX	CERS6	APRT	ZNF587	TM7SF2	CHD1L	CD151
TBC1D9	CRIP2	LOXL1	KAT6B	CD55	RARA	MT1G	SRSF11	TKT	SNX1
EFEMP1	TIMP3	ALDH6A1	ENO2	LXN	PSME4	TMEM176A	SEC24D	EEF1D	FAM173A
KRT6B	STEAP1	C3orf14	NBL1	SHANK2	HNRNPH1	ARFGEF1	NR3C1	SLC35A1	PPAP2A
ACTG2	PLAU	SPARC	FAM198B	IDH2	CD59	COL6A3	SCO2	SSPN	NUAK1
CLU	IGF2	RBPMS	CYBB	IRF9	RBBP4	ARID5B	ECHDC3	COG7	ZKSCAN1
GJA1	MYO10	MAFF	KLF4	SERHL2	FSTL1	SPTAN1	ERI2	PDCD6	FOXO3
AURKA	SEL1L3	MCCC2	FAR2	MPHOSPH6	USP18	HSDL2	MKL2	CXorf40B	ARHGDIA
STAT1	PXDN	WNT5A	TUBA4A	TMEM158	ECT2	H1F0	HSP90AB1	ALDH1A3	G3BP2
STC1	ISOC1	RGS2	F13A1	FAM60A	TSTA3	CYC1	SRPR	ZNF83	IFT46
PMAIP1	GSTM2	WWOX	PTRF	SLC16A1	CTGF	COPA	MFAP3L	SEPT11	DICER1
PSPH	AEBP1	LRIG1	ARMCX1	SIAH2	COX7A1	MBNL1	BCCIP	TARBP1	GRIA2
VAV3	CENPA	AGL	EPHX1	SATB1	TRA2A	MTERFD1	KLHDC10	SON	PDZK1
NTRK2	XAF1	SECTM1	ABCG1	SEPP1	ALG8	WSB2	RSL1D1	HOMER3	HMGCS2
G0S2	GSTM1	IRX5	VEGFA	PCM1	CD93	TACC2	MOCOS	TPP1	SYT13
IGFBP2	TPX2	SERPINI1	MFAP2	GPD1L	ANP32E	PTK2	PHB	IDI1	SLC7A2
RND3	CAP2	AIM1	NEDD9	MS4A6A	ZFP36	SET	POLR2E	SLC11A2	NDP
PRC1	DUSP1	GALNT10	C1orf106	C18orf1	C14orf132	POLI	KRR1	ZNF423	CNTNAP2
CAMK2N1	ITGBL1	CLEC7A	SERPINH1	FLRT2	CRIM1	COL15A1	SNAI2	CD4	SYT1
PSD3	APBB2	GPNMB	GLIPR1	CSAD	USP34	IFITM2	DAAM1	HNRNPA1	PSCA
REEP1	GCH1	PNMAL1	LOXL2	ENC1	CTBP2	ACSL3	HMGB2	RPL31	NOVA1
SCNN1A	HIST1H2BK	EFNB2	AUTS2	DLGAP5	PNPLA4	POLB	EFNA1	PIP4K2A	ATP6V0A4
FAM129A	ACSL1	CD48	PTPRK	POMZP3	HMBOX1	RIN2	TMEM164	SLC43A3	DIO1
C1orf115	MT1E	NREP	MBOAT2	CPVL	CSGALNACT1	GPC1	MYCBP	ECHDC2	PTPRT
SRGN	RGS5	PDLIM5	FXYD5	TMEM123	ADAMTS1	ELF3	KAZN	LIMA1	MMP13
COL1A1	SDC2	DCLK1	ESRP1	SASH1	HPN	TSPAN13	AAGAB	MFN1	LRP2

DCN	ST6GAL1	SLC12A2	PDLIM1	RRBP1	PLAGL1	CANT1	SNX7	SOWAHC	S100A2
GATA3	CECR1	TCF4	ZSCAN18	ENSA	FAH	ACTR2	PICALM	C19orf66	S100B
AKR1C2	TRIM22	MT1X	SUSD4	PLEK	GDE1	OPTN	ACAT1	SLC25A11	EN1
SYBU	PTN	MRC1	FADS1	A2M	PRKD3	HEBP1	SLC19A1	LAMP2	BMPR1B
BST2	COL3A1	CKS2	PADI2	CTSZ	ACTB	CYLD	IQCK	FTO	SOX10
CYP1B1	CRAT	SLC16A3	TMED7	TRIM14	IRF7	TST	ATP6V0E1	YWHAE	GNAZ
GBP1	SGK3	CCL2	SIDT1	CD14	HIF1A	SNRNP25	SCARB2	ZNF107	FABP7
CD24	TRIB2	CDH1	ACLY	CISH	PLEKHA5	DAB2	ACBD3	CDS1	CHD2
HOXB2	GPC4	HSD17B11	STK39	TTC3	LMNB1	FRY	KIF20A	DSCC1	HTATSF1
AKR1C3	COL5A1	ANK3	PTPN13	AIF1	TMEM50B	KIAA1551	PRPF4B	ARMC1	S100A7
NEK2	SORL1	IGFBP3	PMEPA1	LTBP3	YES1	EPB41L3	DNAJC7	BARD1	EPHA4
CELSR1	COL6A1	SC5DL	NECAB3	KMO	HCLS1	LAMP3	AASDHPPT	PRKACB	PALB2
IGF1	KIAA0101	GULP1	DNALI1	GLRX	MRC2	ATAD2	COG2	PKN2	HTR2B
SORBS2	RNASE4	ANKRD36B	LAD1	RAB26	FKBP1A	HMG20B	EPHX2	ARHGDIB	ZCWPW1
KIAA1467	NAMPT	CPE	PLTP	FKBP11	PRELP	PIEZO1	NOTCH2NL	COX17	C18orf25
GPRC5A	PRSS23	CPD	PTGER4	ROBO1	NCF2	PLS1	PSMG1	JUN	CYP2W1
PBK	CYP2B7P1	SULF1	GAMT	GPSM2	P4HA1	SRSF1	POLR2K	ZNF239	SNRNP200
RASGRP1	FBLN1	ME1	ERLIN2	TYROBP	TRIP6	CASP6	CYB5R4	MTA1	CRABP1
CDK1	ALOX5	SOD2	ASB13	MMD	CXCL11	UBE2I	AGT	HSD17B4	PSMB7
HIST1H2BD	ATP1B1	C1QA	SFN	SCAMP1	CHST15	OGT	HTATIP2	NFATC2IP	SLC52A1
TSPAN5	MYH11	NR2F2	ZFP36L1	ASAH1	DAPK1	ETFB	TERF1	GLOD4	U2SURP
GFRA1	MYO6	RRAS2	CDC42BPA	MRPS7	RAB7L1	HOXC4	SLC26A2	PPP2R2A	TAF11
TBX3	OBSL1	TFAP2C	RACGAP1	ENO1	PPP3CA	IQGAP1	MPC2	HOXB7	JRKL
ST6GALNAC2	CPA3	CD44	SOX4	IKBKB	SLC7A11	EPS8L1	PIAS1	UBE2L6	PTGER1
CST1	SUGP1	PSMA6	PPM1F	HBG1	TRMT1	AHSA1	CDC27	CYP24A1	KCNAB1

Supplementary Table 8

Method and feature set	pCR~M + Rx	RFS~M + Rx	DFS~M + Rx
**Supervised centroid classification with PAM50 feature set**			
Baseline PAM50 full	0.740***	0.623**	0.609**
Baseline PAM50 intersecting	0.736***	0.630**	0.585*
BMC PAM50 intersecting	0.734***	0.617**	0.606**
ComBat PAM50 intersecting	0.730***	0.618**	0.617**
**Unsupervised concatenated with intersecting PAM50 feature set**			
Baseline PAM50	0.599	0.584**	0.593**
BMC	Data not available	0.602*	0.577**
ComBat	0.599	0.606**	0.583**
**Unsupervised CoINcIDE**			
PAM50 full centroids (semi-supervised)	0.762***	0.627**	0.609**
PAM50 intersecting	0.762***	0.682**	0.614**
PAM50 full set	0.762***	0.657**	0.620**
PAM50 full set after BMC	0.762***	0.660**	0.630**
meta-rank 50 set	0.712**	0.606	0.666**
meta-rank 264 set	0.757***	0.627	0.647**
meta-rank 2020 set	0.719**	0.657*	0.646**

Supplementary Table 9

Gene	Effect size M1	Effect size M2	Effect size M3	# Datasets M1	# Datasets M2	# Datasets M3
CYP4B1	0.676	NA	NA	15	16	8
KLK7	0.762	0.622	NA	15	16	8
NPR1	0.518	NA	NA	15	16	8
SERPINF1	NA	1.556	NA	15	16	8
MMP11	NA	1.946	NA	15	16	8
AEBP1	NA	1.652	NA	15	16	8
FAP	NA	2.498	NA	15	16	8
MMP2	NA	1.733	NA	15	16	8
CTSK	NA	1.992	NA	15	16	8
PLAU	NA	1.965	NA	15	16	8
TIMP3	NA	1.630	NA	15	16	8
NUAK1	NA	1.500	NA	14	15	7
SERPINE1	NA	1.272	NA	15	16	8
FGFR3	NA	NA	0.551	15	16	8
ALDH1A1	NA	NA	1.649	15	16	8
DDC	NA	NA	0.596	15	16	8

Supplementary Table 10

Gene	Effect size M1	Effect size M2	Effect size M3	# Datasets M1	# Datasets M2	# Datasets M3
CYP4B1	0.67645	NA	NA	15	16	8
KLK7	0.76228	0.62205	NA	15	16	8
NPR1	0.51752	NA	NA	15	16	8
MMP7	NA	0.74977	NA	15	16	8
GPX3	NA	0.53194	NA	15	16	8
KLK6	NA	0.90881	NA	15	16	8
SERPINF1	NA	1.55637	NA	15	16	8
MMP11	NA	1.94638	NA	15	16	8
AEBP1	NA	1.65150	NA	15	16	8
CFB	NA	0.54361	NA	14	15	7
FAP	NA	2.49819	NA	15	16	8
MMP9	NA	0.74418	NA	15	16	8
TNC	NA	0.79770	NA	15	16	8
CYP1B1	NA	0.94609	NA	15	16	8
MMP2	NA	1.73269	NA	15	16	8
CTSK	NA	1.99157	NA	15	16	8
PDGFRA	NA	0.62973	1.05301	15	16	8
DUSP1	NA	0.70201	NA	15	16	8
PLAT	NA	0.73061	NA	15	16	8
COL6A2	NA	1.28045	NA	15	16	8
COL6A3	NA	1.34866	NA	15	16	8
PTGS1	NA	0.59014	NA	15	16	8
C1S	NA	1.24295	NA	14	15	8
PLAU	NA	1.96483	NA	15	16	8
TIMP3	NA	1.63039	NA	15	16	8
SPOCK2	NA	0.64128	NA	15	16	8
NUAK1	NA	1.50000	NA	14	15	7
CD163	NA	1.03071	NA	15	16	8
KAL1	NA	0.94836	NA	15	16	8
C1R	NA	1.17179	NA	15	16	8
SERPINE1	NA	1.27157	NA	15	16	8
COL6A1	NA	1.07178	NA	15	16	8
FGFR3	NA	NA	0.55076	15	16	8
ALDH1A1	NA	NA	1.64857	15	16	8
DDC	NA	NA	0.59553	15	16	8

Supplementary Table 11

Gene	Effect size M1	Effect size M2	Effect size M3	Effect size M4	Effect size M5	Effect size M6	# Datasets M1	# Datasets M2	# Datasets M3	# Datasets M4	# Datasets M5	# Datasets M6
CYP4B1	0.853	NA	NA	NA	NA	NA	16	9	9	6	16	5
KLK7	0.829	NA	NA	NA	NA	NA	16	9	9	6	16	5
CFB	0.675	NA	NA	NA	NA	NA	16	9	9	6	16	5
PTGS1	0.770	NA	NA	NA	NA	NA	16	9	9	6	16	5
NPR1	0.642	NA	NA	NA	NA	NA	16	9	9	6	16	5
GMPR	0.705	NA	NA	NA	NA	NA	16	9	9	6	16	5
PRKCI	0.750	NA	NA	NA	NA	NA	16	9	9	6	16	5
AIFM1	0.838	NA	NA	NA	NA	NA	16	9	9	6	16	5
BDH1	0.686	NA	NA	NA	NA	NA	16	9	9	6	16	5
AFG3L2	0.668	NA	NA	NA	NA	NA	16	9	9	6	16	5
FZD2	NA	0.916	NA	NA	NA	NA	15	8	8	5	15	5
MEST	NA	0.970	NA	NA	NA	NA	16	9	9	6	16	5
CDC7	NA	0.753	NA	NA	NA	NA	16	9	9	6	16	5
ODC1	NA	1.092	NA	NA	NA	NA	16	9	9	6	16	5
ACP1	NA	0.744	NA	NA	NA	NA	16	9	9	6	16	5
APPL2	NA	0.749	NA	NA	NA	1.222	16	9	9	6	16	5
CACNA2D2	NA	0.844	NA	NA	NA	NA	16	9	9	6	16	5
CDK4	NA	0.882	NA	NA	NA	NA	16	9	9	6	15	5
MAP2K6	NA	0.541	1.132	NA	NA	NA	16	9	9	6	16	5
TOP2B	NA	0.738	NA	NA	NA	NA	16	9	9	6	16	5
CAD	NA	0.890	NA	NA	NA	NA	16	9	9	6	16	5
F2RL1	NA	NA	0.699	NA	NA	1.781	16	9	9	6	16	5
SLC7A5	NA	NA	1.196	NA	NA	NA	16	9	9	6	16	5
PCSK6	NA	NA	1.106	0.609	NA	1.028	16	9	9	6	16	5
DHRS7	NA	NA	1.000	1.063	NA	0.915	16	9	9	6	16	5
TST	NA	NA	1.013	NA	NA	1.723	16	9	9	6	16	5
MAOA	NA	NA	1.049	0.521	NA	2.386	16	9	9	6	16	5
HMGCR	NA	NA	1.201	NA	NA	0.796	16	9	9	6	16	5
CPD	NA	NA	0.989	NA	NA	1.355	16	9	9	6	16	5
GABRP	NA	NA	0.947	NA	NA	0.964	16	9	9	6	16	5
ABCC3	NA	NA	0.832	NA	NA	2.490	16	9	9	6	16	5
KIT	NA	NA	0.601	1.697	NA	0.508	16	9	9	6	16	5
PTP4A1	NA	NA	1.276	NA	NA	1.040	16	9	9	6	16	5
PDGFRA	NA	NA	NA	1.480	0.880	NA	16	9	9	6	16	5
NR2F2	NA	NA	NA	1.407	NA	0.661	16	9	9	6	15	5
PDGFRB	NA	NA	NA	1.400	0.922	0.684	16	9	9	6	16	5
PRKACB	NA	NA	NA	0.980	NA	2.079	16	9	9	6	16	5
AKT3	NA	NA	NA	2.480	NA	NA	16	9	9	6	16	5
HSD11B1	NA	NA	NA	1.416	NA	NA	16	9	9	6	16	5
SERPINB6	NA	NA	NA	0.671	NA	1.797	16	9	9	6	16	5
ITPR1	NA	NA	NA	1.934	NA	NA	16	9	9	6	16	5
COL14A1	NA	NA	NA	1.879	NA	NA	16	9	9	6	16	5
CPQ	NA	NA	NA	1.440	NA	NA	16	9	9	6	16	5
TENC1	NA	NA	NA	1.638	NA	NA	16	9	9	6	16	5
MMP11	NA	NA	NA	NA	1.381	NA	16	9	9	6	16	5
FAP	NA	NA	NA	NA	1.758	NA	16	9	9	6	16	5
PLAU	NA	NA	NA	NA	1.506	NA	16	9	9	6	16	5
CD163	NA	NA	NA	NA	1.003	NA	16	9	9	6	16	5

KAL1	NA	NA	NA	NA	0.879	NA	16	9	9	6	16	5
LOXL2	NA	NA	NA	NA	1.360	NA	16	9	9	6	16	5
CSF1R	NA	NA	NA	NA	0.902	NA	16	9	9	6	16	5
SERPINH1	NA	NA	NA	NA	1.024	NA	16	9	9	6	16	5
C3AR1	NA	NA	NA	NA	0.828	NA	16	9	9	6	16	5
ADAM19	NA	NA	NA	NA	0.911	NA	16	9	9	6	16	5
MST1R	NA	NA	NA	NA	NA	2.130	16	9	9	6	16	5
FBP1	NA	NA	NA	NA	NA	1.859	16	9	9	6	16	5
SLC5A1	NA	NA	NA	NA	NA	1.902	16	9	9	6	16	5
DHRS11	NA	NA	NA	NA	NA	1.983	16	9	9	6	16	5
MMP1	NA	NA	NA	NA	NA	2.376	16	9	9	6	16	5
PIK3C2B	NA	NA	NA	NA	NA	1.899	16	9	9	6	16	5
CAPN9	NA	NA	NA	NA	NA	2.079	16	9	9	6	16	5

Supplementary Table 12

Gene	Effect size M1	Effect size M2	Effect size M3	Effect size M4	Effect size M5	Effect size M6	# Datasets M1	# Datasets M2	# Datasets M3	# Datasets M4	# Datasets M5	# Datasets M6
CYP4B1	0.85346	NA	NA	NA	NA	NA	16	9	9	6	16	5
KLK7	0.82866	NA	NA	NA	NA	NA	16	9	9	6	16	5
CFB	0.67474	NA	NA	NA	NA	NA	16	9	9	6	16	5
PTGS1	0.76954	NA	NA	NA	NA	NA	16	9	9	6	16	5
NPR1	0.64205	NA	NA	NA	NA	NA	16	9	9	6	16	5
ESR1	0.59500	NA	NA	NA	NA	NA	16	9	9	6	16	5
GMPR	0.70485	NA	NA	NA	NA	NA	16	9	9	6	16	5
WFDC2	0.58084	NA	NA	NA	NA	NA	16	9	9	6	16	5
PRKCI	0.75022	NA	NA	NA	NA	NA	16	9	9	6	16	5
GPRC5B	0.53630	NA	NA	NA	NA	NA	16	9	9	6	16	5
HTR3A	0.53231	NA	NA	NA	NA	NA	16	9	9	6	16	5
SCNN1A	0.53641	NA	NA	NA	NA	NA	16	9	9	6	16	5
UCP2	0.53961	NA	NA	NA	NA	NA	15	8	8	5	15	5
AIFM1	0.83781	NA	NA	NA	NA	NA	16	9	9	6	16	5
SLC15A2	0.56616	NA	NA	NA	NA	NA	16	9	9	6	16	5
BDH1	0.68621	NA	NA	NA	NA	NA	16	9	9	6	16	5
USP1	0.59701	NA	NA	NA	NA	NA	16	9	9	6	16	5
IRAK1	0.63721	NA	NA	NA	NA	NA	16	9	9	6	15	5
EIF2AK2	0.53633	NA	NA	NA	NA	NA	16	9	9	6	16	5
PTPRF	0.56800	NA	NA	NA	NA	NA	16	9	9	6	16	5
PCCB	0.53870	NA	NA	NA	NA	NA	16	9	9	6	16	5
PSMB2	0.54323	NA	NA	NA	NA	NA	16	9	9	6	16	5
INPPL1	0.60463	NA	NA	NA	NA	NA	16	9	9	6	16	5
AFG3L2	0.66781	NA	NA	NA	NA	NA	16	9	9	6	16	5
UCHL1	NA	0.68771	NA	NA	NA	NA	16	9	9	6	16	5
LPHN2	NA	0.57596	NA	NA	NA	NA	16	9	9	6	16	5
TOP2A	NA	0.64651	NA	NA	NA	NA	16	9	9	6	16	5
FZD2	NA	0.91584	NA	NA	NA	NA	15	8	8	5	15	5
MEST	NA	0.97047	NA	NA	NA	NA	16	9	9	6	16	5
SLC16A1	NA	0.59048	NA	NA	NA	0.79178	16	9	9	6	16	5
CDK1	NA	0.51328	NA	NA	NA	NA	16	9	9	6	16	5
PAM	NA	0.53606	NA	NA	NA	NA	16	9	9	6	16	5
CDC7	NA	0.75299	NA	NA	NA	NA	16	9	9	6	16	5
FYN	NA	0.65292	NA	NA	NA	NA	16	9	9	6	16	5
ODC1	NA	1.09200	NA	NA	NA	NA	16	9	9	6	16	5
GPR161	NA	0.58534	NA	NA	NA	NA	16	9	9	6	16	5
FKBP4	NA	0.51412	NA	NA	NA	NA	16	9	9	6	16	5
NR2F6	NA	0.71774	NA	NA	NA	NA	16	9	9	6	16	5
AURKB	NA	0.62400	NA	NA	NA	NA	16	9	9	6	16	5
ATP2B4	NA	0.58999	NA	NA	NA	NA	16	9	9	6	16	5
ACP1	NA	0.74366	NA	NA	NA	NA	16	9	9	6	16	5
ITGA7	NA	0.67419	NA	NA	NA	NA	16	9	9	6	16	5
ESPL1	NA	0.61942	NA	NA	NA	NA	16	9	9	6	16	5
CACNA1A	NA	0.53922	NA	NA	NA	NA	16	9	9	6	16	5
PRKDC	NA	0.67604	NA	NA	NA	NA	16	9	9	6	16	5
CDK2	NA	0.68650	NA	NA	NA	NA	16	9	9	6	16	5
APPL2	NA	0.74876	NA	NA	NA	1.22249	16	9	9	6	16	5
PMS1	NA	0.59421	NA	NA	NA	NA	16	9	9	6	16	5
PTK7	NA	0.56078	NA	NA	0.53094	NA	16	9	9	6	16	5
BRD3	NA	0.53750	NA	NA	NA	NA	16	9	9	6	16	5
METAP2	NA	0.52024	NA	NA	NA	NA	16	9	9	6	16	5

CHEK1	NA	0.55450	NA	NA	NA	NA	16	9	9	6	16	5
CACNA2D2	NA	0.84352	NA	NA	NA	NA	16	9	9	6	16	5
CDK4	NA	0.88190	NA	NA	NA	NA	16	9	9	6	15	5
MAP2K6	NA	0.54077	1.13222	NA	NA	NA	16	9	9	6	16	5
WEE1	NA	0.63910	NA	NA	NA	NA	16	9	9	6	16	5
NUP153	NA	0.62556	NA	NA	NA	NA	16	9	9	6	16	5
USP11	NA	0.52243	NA	NA	NA	NA	16	9	9	6	16	5
ACVR1B	NA	0.61855	NA	NA	NA	0.96034	16	9	9	6	16	5
APEX1	NA	0.67095	NA	NA	NA	NA	16	9	9	6	16	5
TOP2B	NA	0.73833	NA	NA	NA	NA	16	9	9	6	16	5
DESI2	NA	0.50960	NA	NA	NA	NA	16	9	9	6	16	5
PPOX	NA	0.51429	NA	NA	NA	NA	16	9	9	6	16	5
CSNK2A1	NA	0.73147	NA	NA	NA	NA	16	9	9	6	16	5
CAD	NA	0.89008	NA	NA	NA	NA	16	9	9	6	16	5
CAPN6	NA	0.53567	NA	NA	NA	0.98590	16	9	9	6	16	5
DDX1	NA	0.62423	NA	NA	NA	NA	16	9	9	6	16	5
TRRAP	NA	0.53272	NA	NA	NA	NA	16	9	9	6	16	5
STK25	NA	0.53173	NA	NA	NA	NA	16	9	9	6	16	5
TUBB4A	NA	0.61961	NA	NA	NA	NA	16	9	9	6	16	5
TFPI2	NA	NA	0.54521	NA	NA	NA	16	9	9	6	16	5
SERPINA1	NA	NA	0.84654	NA	NA	0.72290	16	9	9	6	16	5
FGFR3	NA	NA	0.53108	NA	NA	1.28200	16	9	9	6	16	5
GPRC5A	NA	NA	0.77953	NA	NA	1.22637	16	9	9	6	16	5
QPCT	NA	NA	0.63276	NA	NA	NA	16	9	9	6	16	5
SGK1	NA	NA	0.65113	0.82069	0.67315	NA	16	9	9	6	16	5
CA8	NA	NA	0.74296	NA	NA	NA	16	9	9	6	16	5
F2RL1	NA	NA	0.69904	NA	NA	1.78149	16	9	9	6	16	5
SPOCK1	NA	NA	0.67338	NA	0.68593	NA	16	9	9	6	16	5
SLC7A5	NA	NA	1.19619	NA	NA	NA	16	9	9	6	16	5
ADAM9	NA	NA	0.79630	NA	0.67320	0.75101	16	9	9	6	16	5
PNP	NA	NA	1.00002	NA	NA	0.60749	16	9	9	6	16	5
CTSC	NA	NA	0.72806	NA	0.55755	NA	16	9	9	6	16	5
PCSK6	NA	NA	1.10583	0.60914	NA	1.02815	16	9	9	6	16	5
PIK3R1	NA	NA	0.54225	NA	NA	NA	16	9	9	6	16	5
SLC7A1	NA	NA	0.73208	NA	NA	NA	16	9	9	6	16	5
DAPK1	NA	NA	0.71205	0.88899	NA	1.04167	16	9	9	6	16	5
SORD	NA	NA	0.65579	NA	NA	NA	16	9	9	6	16	5
CPM	NA	NA	0.70595	NA	NA	NA	16	9	9	6	16	5
DHRS7	NA	NA	1.00016	1.06327	NA	0.91480	16	9	9	6	16	5
CTSH	NA	NA	0.73613	NA	NA	0.50996	16	9	9	6	16	5
TFPI	NA	NA	0.57401	1.02165	NA	1.48675	16	9	9	6	16	5
ABCG1	NA	NA	0.55176	NA	NA	NA	16	9	9	6	16	5
TST	NA	NA	1.01318	NA	NA	1.72257	16	9	9	6	16	5
MAOA	NA	NA	1.04890	0.52091	NA	2.38577	16	9	9	6	16	5
HMGCR	NA	NA	1.20111	NA	NA	0.79607	16	9	9	6	16	5
QSOX1	NA	NA	0.81039	NA	NA	1.12175	16	9	9	6	16	5
CPD	NA	NA	0.98882	NA	NA	1.35544	16	9	9	6	16	5
GSR	NA	NA	0.93014	NA	NA	0.82734	16	9	9	6	16	5
GABRP	NA	NA	0.94710	NA	NA	0.96363	16	9	9	6	16	5
ABCC3	NA	NA	0.83178	NA	NA	2.48980	16	9	9	6	16	5
ITGA6	NA	NA	0.64725	NA	NA	1.41577	16	9	9	6	16	5
KIT	NA	NA	0.60115	1.69652	NA	0.50807	16	9	9	6	16	5
BAX	NA	NA	0.70278	NA	NA	NA	16	9	9	6	16	5
ALDH6A1	NA	NA	0.54710	NA	NA	NA	16	9	9	6	16	5
RIPK2	NA	NA	0.75149	NA	NA	NA	16	9	9	6	16	5

PTPN3	NA	NA	0.91047	NA	NA	NA	16	9	9	6	16	5
DUSP4	NA	NA	0.58823	NA	NA	0.91168	16	9	9	6	16	5
APLP2	NA	NA	0.56634	NA	NA	NA	16	9	9	6	16	5
PTGS2	NA	NA	0.88698	NA	NA	NA	16	9	9	6	16	5
FUCA1	NA	NA	0.67143	0.61670	NA	0.72303	16	9	9	6	16	5
ABCD3	NA	NA	0.56972	NA	NA	1.38135	16	9	9	6	16	5
GPR143	NA	NA	0.68059	NA	NA	NA	16	9	9	6	16	5
POR	NA	NA	0.66519	NA	NA	NA	16	9	9	6	16	5
ABCA5	NA	NA	0.66904	NA	NA	1.09297	16	9	9	6	16	5
C5AR1	NA	NA	0.67118	NA	0.78026	NA	16	9	9	6	16	5
PRKCH	NA	NA	0.51136	NA	NA	NA	16	9	9	6	16	5
GBE1	NA	NA	0.93310	NA	NA	NA	16	9	9	6	16	5
P4HB	NA	NA	0.70867	NA	NA	0.82530	16	9	9	6	16	5
PTP4A1	NA	NA	1.27558	NA	NA	1.04016	16	9	9	6	16	5
TXNDC9	NA	NA	0.61561	NA	NA	NA	16	9	9	6	16	5
RPS6KA5	NA	NA	0.64415	0.51211	NA	NA	16	9	9	6	16	5
CDK7	NA	NA	0.77868	NA	NA	NA	16	9	9	6	16	5
ERN1	NA	NA	0.69165	NA	NA	0.86467	16	9	9	6	16	5
CLCN3	NA	NA	0.64416	0.63822	NA	1.69648	16	9	9	6	16	5
ATP2A2	NA	NA	0.63467	NA	0.51413	NA	16	9	9	6	16	5
PDK3	NA	NA	0.55544	NA	NA	NA	16	9	9	6	16	5
DECR1	NA	NA	0.63680	NA	NA	NA	16	9	9	6	16	5
SLCO2A1	NA	NA	0.50160	NA	NA	NA	16	9	9	6	16	5
HSD17B4	NA	NA	0.68128	NA	NA	0.56505	16	9	9	6	16	5
DPP4	NA	NA	0.61724	NA	NA	1.78968	16	9	9	6	16	5
CYB5R3	NA	NA	0.72410	0.92222	0.50989	0.65109	16	9	9	6	16	5
KCNQ1	NA	NA	0.61120	NA	NA	1.21183	16	9	9	6	16	5
PSEN1	NA	NA	0.55100	NA	NA	NA	16	9	9	6	16	5
PTPRB	NA	NA	0.58471	1.25852	NA	1.33158	16	9	9	6	16	5
SERPINF1	NA	NA	NA	1.12684	1.43832	NA	16	9	9	6	16	5
AEBP1	NA	NA	NA	1.14443	1.54421	NA	16	9	9	6	16	5
SERPINE2	NA	NA	NA	1.00766	NA	NA	16	9	9	6	16	5
MMP2	NA	NA	NA	0.66230	1.48517	NA	16	9	9	6	16	5
CTSK	NA	NA	NA	0.85014	1.52482	NA	16	9	9	6	16	5
PDGFRA	NA	NA	NA	1.47992	0.88008	NA	16	9	9	6	16	5
DUSP1	NA	NA	NA	0.63961	0.68810	NA	16	9	9	6	16	5
COL6A2	NA	NA	NA	0.97528	1.12578	NA	16	9	9	6	16	5
COL6A3	NA	NA	NA	0.82086	1.36073	NA	16	9	9	6	16	5
C1S	NA	NA	NA	1.08453	1.18367	NA	16	9	9	6	15	5
TIMP3	NA	NA	NA	0.68107	1.27647	NA	16	9	9	6	16	5
ALDH1A1	NA	NA	NA	1.29947	NA	1.65652	16	9	9	6	16	5
NUAK1	NA	NA	NA	0.67126	1.38112	NA	16	9	9	6	16	5
NR4A2	NA	NA	NA	0.93918	NA	NA	16	9	9	6	16	5
C1R	NA	NA	NA	1.06217	1.07313	NA	16	9	9	6	16	5
MATN2	NA	NA	NA	0.54396	NA	0.91375	16	9	9	6	16	5
AR	NA	NA	NA	0.52042	NA	NA	16	9	9	6	16	5
CSTA	NA	NA	NA	0.95605	NA	NA	16	9	9	6	16	5
DPYD	NA	NA	NA	1.01231	0.73747	0.53495	16	9	9	6	16	5
HTRA1	NA	NA	NA	1.14642	0.94274	NA	16	9	9	6	16	5
ALDH1A3	NA	NA	NA	0.67067	1.06551	NA	16	9	9	6	15	5
COL6A1	NA	NA	NA	0.85545	1.03905	NA	16	9	9	6	16	5
SERPINE1	NA	NA	NA	0.55066	1.18503	NA	16	9	9	6	16	5
ENPP2	NA	NA	NA	0.99609	NA	NA	16	9	9	6	16	5
CPE	NA	NA	NA	0.64659	0.66753	NA	16	9	9	6	16	5
TRIM22	NA	NA	NA	0.65342	0.78708	NA	16	9	9	6	16	5

FZD7	NA	NA	NA	0.53962	0.53160	NA	16	9	9	6	16	5
FGL2	NA	NA	NA	0.79152	0.77056	NA	16	9	9	6	16	5
PDE4B	NA	NA	NA	0.62504	0.69361	NA	16	9	9	6	15	5
PCSK5	NA	NA	NA	0.87896	0.63378	0.69377	16	9	9	6	16	5
MAOB	NA	NA	NA	1.32154	NA	1.26153	16	9	9	6	16	5
A2M	NA	NA	NA	0.82378	0.79921	NA	16	9	9	6	16	5
MFAP4	NA	NA	NA	1.13400	0.55965	NA	15	8	8	5	15	5
ADRA2A	NA	NA	NA	0.98785	0.52642	1.44933	16	9	9	6	16	5
PRKCA	NA	NA	NA	0.56742	NA	1.37649	16	9	9	6	16	5
TGFBR2	NA	NA	NA	0.51167	0.81471	0.81981	16	9	9	6	16	5
LPAR1	NA	NA	NA	0.55525	NA	NA	16	9	9	6	16	5
BCL2	NA	NA	NA	0.97873	NA	NA	16	9	9	6	16	5
GUCY1A3	NA	NA	NA	0.87795	0.77055	NA	16	9	9	6	16	5
CFD	NA	NA	NA	1.24945	0.57594	NA	16	9	9	6	16	5
CDK14	NA	NA	NA	0.55582	0.65368	NA	16	9	9	6	16	5
NR2F2	NA	NA	NA	1.40681	NA	0.66111	16	9	9	6	15	5
EDNRA	NA	NA	NA	1.13761	1.04664	NA	16	9	9	6	16	5
TIMP1	NA	NA	NA	1.03375	NA	NA	16	9	9	6	16	5
TIMP2	NA	NA	NA	1.23957	0.73438	NA	16	9	9	6	16	5
VWF	NA	NA	NA	1.04866	NA	1.24009	16	9	9	6	16	5
CRIM1	NA	NA	NA	0.80623	NA	NA	16	9	9	6	16	5
PRKD1	NA	NA	NA	1.02215	0.61762	NA	16	9	9	6	15	5
NR4A1	NA	NA	NA	1.06600	NA	NA	16	9	9	6	16	5
PDGFRB	NA	NA	NA	1.39989	0.92194	0.68422	16	9	9	6	16	5
PRKACB	NA	NA	NA	0.98013	NA	2.07853	16	9	9	6	16	5
ENPP1	NA	NA	NA	0.98692	0.78535	NA	16	9	9	6	16	5
MAP3K5	NA	NA	NA	1.12294	NA	1.24480	16	9	9	6	16	5
AKT3	NA	NA	NA	2.47971	NA	NA	16	9	9	6	16	5
HSD11B1	NA	NA	NA	1.41594	NA	NA	16	9	9	6	16	5
DCLK1	NA	NA	NA	0.87999	NA	NA	16	9	9	6	16	5
OAT	NA	NA	NA	0.57104	NA	0.59498	16	9	9	6	16	5
DDR2	NA	NA	NA	1.26398	0.65806	NA	16	9	9	6	16	5
NR3C1	NA	NA	NA	0.64648	0.64969	NA	16	9	9	6	16	5
AXL	NA	NA	NA	0.78370	0.62918	NA	16	9	9	6	16	5
PTGER2	NA	NA	NA	0.63188	NA	NA	16	9	9	6	16	5
SERPINB6	NA	NA	NA	0.67109	NA	1.79692	16	9	9	6	16	5
NR3C2	NA	NA	NA	1.20096	NA	1.44430	16	9	9	6	16	5
LPL	NA	NA	NA	0.54316	0.54779	NA	16	9	9	6	16	5
PKD2	NA	NA	NA	1.18397	0.74363	NA	16	9	9	6	16	5
GABBR1	NA	NA	NA	0.89770	NA	NA	16	9	9	6	16	5
ITPR1	NA	NA	NA	1.93420	NA	NA	16	9	9	6	16	5
INSR	NA	NA	NA	0.89268	NA	0.88333	16	9	9	6	16	5
IDS	NA	NA	NA	0.58603	NA	NA	16	9	9	6	16	5
MAN2A1	NA	NA	NA	0.69829	0.52522	1.08912	16	9	9	6	16	5
ITGA5	NA	NA	NA	0.55510	1.11291	NA	16	9	9	6	16	5
CLK1	NA	NA	NA	0.73246	NA	NA	16	9	9	6	16	5
HSD17B6	NA	NA	NA	0.67835	0.88866	0.51720	16	9	9	6	16	5
NR4A3	NA	NA	NA	1.04277	NA	NA	16	9	9	6	16	5
RORA	NA	NA	NA	1.05369	NA	NA	16	9	9	6	16	5
COL14A1	NA	NA	NA	1.87883	NA	NA	16	9	9	6	16	5
CPQ	NA	NA	NA	1.44002	NA	NA	16	9	9	6	16	5
ADAMTS2	NA	NA	NA	0.52994	0.73344	NA	16	9	9	6	16	5
KCNJ8	NA	NA	NA	1.12568	NA	NA	16	9	9	6	16	5
TIE1	NA	NA	NA	0.84156	NA	0.50334	16	9	9	6	16	5
PIM1	NA	NA	NA	1.17526	0.50851	NA	16	9	9	6	16	5

MTMR6	NA	NA	NA	0.68504	0.52125	0.74788	16	9	9	6	16	5
SOD3	NA	NA	NA	0.50694	NA	NA	16	9	9	6	16	5
JAK1	NA	NA	NA	0.63971	0.55843	NA	16	9	9	6	16	5
SLC16A2	NA	NA	NA	0.91050	NA	0.80140	16	9	9	6	16	5
TENC1	NA	NA	NA	1.63826	NA	NA	16	9	9	6	16	5
PDK4	NA	NA	NA	0.99660	NA	0.63646	16	9	9	6	16	5
CAMKK2	NA	NA	NA	0.59549	NA	NA	16	9	9	6	16	5
NFATC1	NA	NA	NA	0.58846	NA	NA	16	9	9	6	16	5
PRCP	NA	NA	NA	0.52483	NA	NA	16	9	9	6	16	5
SLC4A3	NA	NA	NA	0.95603	NA	NA	16	9	9	6	16	5
P2RX7	NA	NA	NA	0.79053	0.59368	NA	16	9	9	6	16	5
CAPN3	NA	NA	NA	0.55714	NA	NA	16	9	9	6	16	5
EPHA5	NA	NA	NA	1.33782	NA	NA	16	9	9	6	16	5
MMP7	NA	NA	NA	NA	0.52687	NA	16	9	9	6	16	5
GPX3	NA	NA	NA	NA	0.52638	NA	16	9	9	6	16	5
KLK6	NA	NA	NA	NA	0.52069	NA	16	9	9	6	16	5
MMP11	NA	NA	NA	NA	1.38141	NA	16	9	9	6	16	5
FAP	NA	NA	NA	NA	1.75780	NA	16	9	9	6	16	5
MMP9	NA	NA	NA	NA	0.63268	NA	16	9	9	6	16	5
TNC	NA	NA	NA	NA	0.71731	NA	16	9	9	6	16	5
CYP1B1	NA	NA	NA	NA	0.91445	NA	16	9	9	6	16	5
PLAT	NA	NA	NA	NA	0.68984	0.68611	16	9	9	6	16	5
PLAU	NA	NA	NA	NA	1.50562	NA	16	9	9	6	16	5
CD163	NA	NA	NA	NA	1.00298	NA	16	9	9	6	16	5
KAL1	NA	NA	NA	NA	0.87939	NA	16	9	9	6	16	5
CXCR4	NA	NA	NA	NA	0.51406	NA	16	9	9	6	16	5
CTSS	NA	NA	NA	NA	0.63104	0.78520	16	9	9	6	16	5
CPVL	NA	NA	NA	NA	0.57771	NA	16	9	9	6	16	5
GUCY1B3	NA	NA	NA	NA	0.50643	NA	16	9	9	6	16	5
LOXL2	NA	NA	NA	NA	1.36013	NA	16	9	9	6	16	5
ALOX5AP	NA	NA	NA	NA	0.70809	NA	16	9	9	6	16	5
TNFAIP3	NA	NA	NA	NA	0.74468	NA	16	9	9	6	16	5
PPIC	NA	NA	NA	NA	0.78432	NA	16	9	9	6	16	5
VDR	NA	NA	NA	NA	0.58420	0.64729	16	9	9	6	16	5
PTPRC	NA	NA	NA	NA	0.75391	NA	16	9	9	6	16	5
GZMA	NA	NA	NA	NA	0.65851	NA	16	9	9	6	16	5
GPR183	NA	NA	NA	NA	0.75047	NA	16	9	9	6	16	5
CSF1R	NA	NA	NA	NA	0.90175	NA	16	9	9	6	16	5
CCR1	NA	NA	NA	NA	0.81316	NA	16	9	9	6	16	5
LYN	NA	NA	NA	NA	0.61465	NA	16	9	9	6	16	5
ITGB5	NA	NA	NA	NA	0.79356	NA	16	9	9	6	16	5
LGMN	NA	NA	NA	NA	0.71742	NA	16	9	9	6	16	5
ITGAV	NA	NA	NA	NA	0.86028	NA	16	9	9	6	16	5
SERPINH1	NA	NA	NA	NA	1.02375	NA	16	9	9	6	16	5
BCL2A1	NA	NA	NA	NA	0.62660	NA	15	8	8	5	15	5
C3AR1	NA	NA	NA	NA	0.82755	NA	16	9	9	6	16	5
ROR2	NA	NA	NA	NA	0.78307	NA	16	9	9	6	16	5
MAP4K4	NA	NA	NA	NA	0.64332	NA	16	9	9	6	16	5
CTSD	NA	NA	NA	NA	0.59820	NA	16	9	9	6	16	5
PIK3CD	NA	NA	NA	NA	0.71672	NA	16	9	9	6	16	5
CAST	NA	NA	NA	NA	0.53332	0.85386	15	8	8	5	15	5
FPR1	NA	NA	NA	NA	0.61644	NA	16	9	9	6	16	5
ACVR1	NA	NA	NA	NA	0.61762	NA	16	9	9	6	16	5
ADAM17	NA	NA	NA	NA	0.50793	NA	16	9	9	6	16	5
ADAM19	NA	NA	NA	NA	0.91138	NA	16	9	9	6	16	5

CCR5	NA	NA	NA	NA		0.63230	NA	16	9	9	6	16	5
SPSB1	NA	NA	NA	NA		0.69747	NA	16	9	9	6	16	5
ZMPSTE24	NA	NA	NA	NA		0.50118	NA	16	9	9	6	16	5
STK38	NA	NA	NA	NA		0.52492	0.53004	16	9	9	6	16	5
SLC2A5	NA	NA	NA	NA		0.52795	NA	16	9	9	6	16	5
F2R	NA	NA	NA	NA		0.58536	NA	16	9	9	6	16	5
DUSP5	NA	NA	NA	NA	NA		0.55117	16	9	9	6	16	5
ITGB4	NA	NA	NA	NA	NA		0.91754	16	9	9	6	16	5
ALDH2	NA	NA	NA	NA	NA		1.17245	16	9	9	6	16	5
SLC6A8	NA	NA	NA	NA	NA		1.17533	16	9	9	6	16	5
ANPEP	NA	NA	NA	NA	NA		1.51839	16	9	9	6	16	5
SERPINB1	NA	NA	NA	NA	NA		1.59629	16	9	9	6	16	5
ERBB3	NA	NA	NA	NA	NA		1.36364	16	9	9	6	16	5
TP53I3	NA	NA	NA	NA	NA		0.65611	16	9	9	6	16	5
DHRS3	NA	NA	NA	NA	NA		0.63117	16	9	9	6	16	5
CA2	NA	NA	NA	NA	NA		1.73642	16	9	9	6	16	5
MST1R	NA	NA	NA	NA	NA		2.12961	16	9	9	6	16	5
ITPR3	NA	NA	NA	NA	NA		0.77292	16	9	9	6	16	5
FDFT1	NA	NA	NA	NA	NA		0.79063	16	9	9	6	16	5
AADAC	NA	NA	NA	NA	NA		1.31235	16	9	9	6	16	5
TXN	NA	NA	NA	NA	NA		1.06214	16	9	9	6	16	5
PRPS2	NA	NA	NA	NA	NA		0.94253	16	9	9	6	16	5
FZD5	NA	NA	NA	NA	NA		1.32368	16	9	9	6	16	5
CA9	NA	NA	NA	NA	NA		1.39374	16	9	9	6	16	5
PTPRK	NA	NA	NA	NA	NA		1.32840	16	9	9	6	16	5
FBP1	NA	NA	NA	NA	NA		1.85894	16	9	9	6	16	5
CFTR	NA	NA	NA	NA	NA		1.20935	16	9	9	6	16	5
TRIM14	NA	NA	NA	NA	NA		0.58236	16	9	9	6	16	5
CD97	NA	NA	NA	NA	NA		1.16806	16	9	9	6	16	5
CASP6	NA	NA	NA	NA	NA		0.56901	16	9	9	6	16	5
SLC5A1	NA	NA	NA	NA	NA		1.90247	16	9	9	6	16	5
CAPN2	NA	NA	NA	NA	NA		0.62160	16	9	9	6	16	5
DHRS11	NA	NA	NA	NA	NA		1.98262	16	9	9	6	16	5
PRKCD	NA	NA	NA	NA	NA		0.64296	16	9	9	6	16	5
MMP1	NA	NA	NA	NA	NA		2.37611	16	9	9	6	16	5
TSPO	NA	NA	NA	NA	NA		0.77656	16	9	9	6	16	5
ERBB2	NA	NA	NA	NA	NA		1.58983	15	8	8	5	15	5
HSD11B2	NA	NA	NA	NA	NA		0.77834	16	9	9	6	16	5
SLC25A5	NA	NA	NA	NA	NA		0.72440	16	9	9	6	16	5
CYP2J2	NA	NA	NA	NA	NA		1.13372	16	9	9	6	16	5
SLCO2B1	NA	NA	NA	NA	NA		1.03705	16	9	9	6	16	5
RPS6KA3	NA	NA	NA	NA	NA		1.43920	16	9	9	6	16	5
ST14	NA	NA	NA	NA	NA		1.02491	16	9	9	6	16	5
UCHL3	NA	NA	NA	NA	NA		0.90087	16	9	9	6	16	5
EGFR	NA	NA	NA	NA	NA		0.63017	16	9	9	6	16	5
CASP4	NA	NA	NA	NA	NA		0.50062	16	9	9	6	16	5
MGST2	NA	NA	NA	NA	NA		1.40018	16	9	9	6	16	5
F12	NA	NA	NA	NA	NA		1.44928	16	9	9	6	16	5
CASP7	NA	NA	NA	NA	NA		0.91042	16	9	9	6	16	5
SRPK1	NA	NA	NA	NA	NA		0.54248	16	9	9	6	16	5
PSMA5	NA	NA	NA	NA	NA		0.55744	16	9	9	6	16	5
HMGCS1	NA	NA	NA	NA	NA		0.88238	16	9	9	6	16	5
FRK	NA	NA	NA	NA	NA		0.85078	16	9	9	6	16	5
SLC12A2	NA	NA	NA	NA	NA		1.83256	16	9	9	6	16	5
GUSB	NA	NA	NA	NA	NA		0.61161	16	9	9	6	16	5

SLK	NA	NA	NA	NA	NA	0.94688	16	9	9	6	16	5
INPP5A	NA	NA	NA	NA	NA	0.90073	16	9	9	6	16	5
PIK3C2B	NA	NA	NA	NA	NA	1.89873	16	9	9	6	16	5
CAPN9	NA	NA	NA	NA	NA	2.07889	16	9	9	6	16	5
EPHB4	NA	NA	NA	NA	NA	1.05267	16	9	9	6	16	5
ITGB6	NA	NA	NA	NA	NA	0.62233	16	9	9	6	16	5
SLC25A24	NA	NA	NA	NA	NA	0.54904	16	9	9	6	16	5
PLCG2	NA	NA	NA	NA	NA	0.53965	16	9	9	6	16	5
ROCK2	NA	NA	NA	NA	NA	1.48436	16	9	9	6	16	5
PTGER4	NA	NA	NA	NA	NA	0.88315	16	9	9	6	16	5
RDH5	NA	NA	NA	NA	NA	1.09843	16	9	9	6	16	5
ATP1A1	NA	NA	NA	NA	NA	1.35269	16	9	9	6	16	5
PSMB3	NA	NA	NA	NA	NA	0.74569	16	9	9	6	16	5
CASP10	NA	NA	NA	NA	NA	0.69652	16	9	9	6	16	5
USP48	NA	NA	NA	NA	NA	0.59569	16	9	9	6	16	5
DDC	NA	NA	NA	NA	NA	4.43655	16	9	9	6	16	5

Supplementary Table 13

Label in figures	Dataset ID	Number of clusters
1	GSE1379	3
2	GSE2034	5
3	GSE9893	5
4	GSE12093	4
5	GSE16391	3
6	GSE16446	5
7	GSE17705.JBI	4
8	GSE17705_MDACC	4
9	GSE19615	5
10	GSE20181	2
11	GSE20194	5
12	GSE22226	4
13	GSE22358	5
14	GSE25055	5
15	GSE25065.MDACC	4
16	GSE25055.USO	4
17	GSE32646	3

Supplementary Table 14

Label in figures	Dataset ID	Number of clusters
1	GSE1379	5
2	GSE2034	4
3	GSE9893	2
4	GSE12093	3
5	GSE16391	2
6	GSE16446	3
7	GSE17705.JBI	2
8	GSE17705_MDACC	1
9	GSE19615	2
10	GSE20181	2
11	GSE20194	4
12	GSE22226	5
13	GSE22358	2
14	GSE25055	2
15	GSE25065.MDACC	2
16	GSE25055.USO	2
17	GSE32646	1

Supplementary Table 15

Label in figures	Dataset ID	Number of clusters
1	GSE1379	5
2	GSE2034	3
3	GSE9893	2
4	GSE12093	3
5	GSE16391	2
6	GSE16446	2
7	GSE17705.JBI	2
8	GSE17705_MDACC	2
9	GSE19615	4
10	GSE20181	2
11	GSE20194	3
12	GSE22226	4
13	GSE22358	2
14	GSE25055	3
15	GSE25065.MDACC	2
16	GSE25055.USO	2
17	GSE32646	1

Supplementary Table 16

Label in figures	Dataset ID	Number of clusters
1	GSE1379	1
2	GSE2034	2
3	GSE9893	2
4	GSE12093	2
5	GSE16391	1
6	GSE16446	2
7	GSE17705.JBI	1
8	GSE17705_MDACC	1
9	GSE19615	2
10	GSE20181	1
11	GSE20194	2
12	GSE22226	2
13	GSE22358	2
14	GSE25055	2
15	GSE25065.MDACC	2
16	GSE25055.USO	2
17	GSE32646	1

Supplementary Table 17

Label in figu	Dataset ID	Number of clusters
1	GSE1379	3
2	GSE2034	2
3	GSE9893	2
4	GSE12093	2
5	GSE16391	10
6	GSE16446	2
7	GSE17705.JBI	8
8	GSE17705_MDACC	3
9	GSE19615	2
10	GSE20181	1
11	GSE20194	4
12	GSE22226	2
13	GSE22358	4
14	GSE25055	2
15	GSE25065.MDACC	2
16	GSE25055.USO	2
17	GSE32646	6

Supplementary Table 18

Label in figures	Dataset ID	Number of clusters
1	GSE1379	1
2	GSE2034	2
3	GSE9893	2
4	GSE12093	3
5	GSE16391	2
6	GSE16446	2
7	GSE17705.JBI	2
8	GSE17705_MDACC	4
9	GSE19615	4
10	GSE20181	4
11	GSE20194	4
12	GSE22358	3
13	GSE25055	3
14	GSE25065.MDACC	2
15	GSE25055.USO	2
16	GSE32646	4

Supplementary Table 19

Label in figures	Dataset ID	Number of clusters
1	E.MTAB.386	3
2	GSE12470	2
3	GSE13876	2
4	GSE14764	1
5	GSE17260	10
6	GSE18520	1
7	GSE19829.GPL570	5
8	GSE19829.GPL8300	6
9	GSE20565	2
10	GSE2109	2
11	GSE26193	6
12	GSE26712	1
13	GSE30161	2
14	GSE32062.GPL6480	3
15	GSE32063	2
16	GSE44104	7
17	GSE49997	2
18	GSE6008	2
19	GSE6822	7
20	GSE9891	2
21	PMID15897565	2
22	PMID17290060	9
23	PMID19318476	10
24	TCGA	3

Supplementary Table 20

Label in figures	Dataset ID	Number of clusters
1	E.MTAB.386	2
2	GSE12470	10
3	GSE13876	3
4	GSE14764	2
5	GSE17260	2
6	GSE18520	4
7	GSE19829.GPL570	10
8	GSE19829.GPL8300	1
9	GSE20565	7
10	GSE2109	6
11	GSE26193	8
12	GSE26712	2
13	GSE30161	5
14	GSE32062.GPL6480	2
15	GSE32063	5
16	GSE44104	3
17	GSE49997	4
18	GSE6008	5
19	GSE9891	7
20	PMID15897565	2
21	PMID17290060	2
22	PMID19318476	10
23	TCGA	3
