# Supplementary material for: CoINcIDE: A framework for discovery of patient subtypes across multiple datasets
Source: Genome Med. 2016 Mar 9;8:27. doi: 10.1186/s13073-016-0281-4 (PMC4784276; doi:10.1186/s13073-016-0281-4)
Supplement: Additional file 2: — Additional methods details to supplement the main Methods section. (PDF 471 kb) [file 13073_2016_281_MOESM2_ESM.pdf]

Additional file 2

Supplemental Methods

Gene Meta-Rank Algorithm

Within each dataset, all genes are ranked via the median absolute deviation (mad) in decreasing order, so that genes with the largest median absolute deviations are ranked at the top. Then, the median rank of each gene is computed across all datasets; the user selects the number of G meta-rank genes to return. It is not assumed that all datasets contain all possible subtypes, and thus not all key genes that distinguish clusters may be highly ranked using solely these G genes. To remedy this, the algorithm also takes the top N genes by mad for each dataset, called the "intra-rank" genes; the top 20 intra-rank genes from each dataset were taken for the breast and ovarian meta-rank analyses in this publication. The final number of genes in the returned set is the union of the G meta-rank and the N intra-rank genes. The user can also specify the number of missing datasets allowed per gene (we suggest a maximum of 10% of all input datasets) and the maximum fraction of top meta-ranked missing genes allowed in a dataset (we recommend 0.2). Datasets with a fraction of top-ranked genes that are missing above this latter threshold are removed from the CoINcIDE analysis.

Baseline Null Experimental Tests

To confirm that a specific CoINcIDE experiment using a set of specific edge thresholds would not occur at random on null datasets, CoINcIDE is also run using null datasets to see if there is a high probability of any spurious edges being produced. Null datasets that follow a similar correlation structure to that of the real input datasets are created using the same methods used to derive the null centroid sets for each cluster-cluster p-value; now,

each column is a null patient sample as opposed to a null centroid. Null datasets are separated into null clusters by assigning null patient samples to a cluster using the patient (column) cluster assignments from the real dataset (see main Methods for details.) These null clusters are then fed into CoINcIDE to calculate the probability of obtaining a spurious edge; we interpret this probability as the number of null edges detected in the final null network divided by the total possible number of null edges. The same user-defined thresholds are used to compute this probability as in the real CoINcIDE analysis to aide in threshold tuning. The final output probability is an average of the probabilities calculated across several iterations of null datasets; 10 iterations were used for all analyses in this publication. The spurious edge probability for all breast and ovarian real-dataset analyses, even before removing meta-clusters with less than three unique datasets, was below 1.0%.

Simulation Methods

To simulate highly distinct patient clusters, the lung200 Bhattacharjee dataset from the s4vd R package [1] was used. This dataset contains logged gene expression microarrays from four different tissue types: 20 pulmonary carcinoid, 6 small cell lung, 13 colon metastasis and 17 normal lung tissue samples. The top 200 varying genes across the entire dataset were included. Datasets were simulated that mimicked the strong patient-patient correlation within each tissue type, and conversely low patient-patient correlation between different tissue types, using Eigen decomposition [2] in R. The covariance matrix $C_{N,N}$ from the true data matrix $X_{G,N}$ with g genes in the rows and n patients in columns (Equation 1) was factorized into the matrix VDV^T. The diagonal Eigen value matrix D and the Eigen vector matrix V were multiplied to create a square orthonormal

matrix DV (Equation 1). A matrix $Z_{T,N}$ drawn from a random standard normal distribution with T genes in the rows and N patients in the columns was then multiplied by DV to achieve the final simulated data matrix $S_{T,N}$ (Equations 2 and 3 below). Using either the covariance or correlation matrix of X gives similar results.

These calculations are not dependent upon the number of t rows/genes in Z. The Eigen decomposition matrix methods were developed to purposefully capture patient-patient correlation, and not necessarily gene-gene correlation patterns. Thus, this simulated data is not ideal for testing the specific effects of differing gene set sizes. t=g=200 was chosen to match the feature set size of the original dataset.

$$C_{N,N} = \mathrm{cov}\left(X_{G,N}\right) = VDV^{T} \; [1]$$

$$Z_{T,N} \sim N(0,1) \; [2]$$

$$S_{T,N} = ZDV \; [3]$$

To simulate tissue clusters with differing number of patients, N' patients were resampled with replacement from the true tissue cluster before Eigen decomposition, where n' does not necessarily equal the original sample size N. Figure S4A in Additional File 1 illustrates the patient-patient correlation patterns of the real dataset, and Figure S4B-D in Additional File 1 display examples of simulated renderings of Pearson's patient-patient correlation matrices.

We simulated seven different sets of clustered datasets using the Eigen decomposition methods detailed earlier, with each set containing ten datasets constructed in a similar manner. Unless noted, we defined a cluster to contain all objects of the same tissue type.

All clusters contained 200 features. The first set contained datasets with four evenly sized clusters of 50 objects, one cluster per tissue type. The second set of datasets contained four clusters derived in a similar manner to set one, but we assigned each cluster random sample size ranging from one to 100 objects.

The third set contained clusters derived in a similar manner to set one, but we randomly selected the number of clusters/tissue types within each dataset to be between two and the full number of clusters four, allowing a different number of clusters across all 10 datasets. The fourth set contained clusters derived in a similar manner to set one, but we randomly selected the number of clusters within each dataset to be between one and the full number of clusters four, allowing a different number of clusters across all 10 datasets.

The fifth and sixth sets combined the different cluster sizes from set three with the number of clusters in each dataset ranging from two to four and then one to four, respectively. Finally, the seventh set contained two evenly sized clusters, with similar pulmonary carcinoid and small cell carcinoma samples, but for the second two clusters, we replaced the metastatic colon and normal lung clusters with randomly selected samples from across all four tissue types to create two highly noisy clusters for each dataset.

For each of these seven clustering simulation scenarios, we added random normal noise with a mean of zero and increasing standard deviation from 0 to 2.4 to each of the ten datasets. We simulated each set 50 times and ran CoINcIDE each time. We computed TPR and FPR values and averaged them across all 50 iterations for each set.

Preprocessing gene expression data

Whenever raw microarray data was available, gene expression datasets were consistently

normalized in R using on of two pipelines: the GeneChip Robust Multi-array Average (GCRMA) method [3] from the gcrma R package for 1-channel arrays or RMA background correction, within-array Local Regression (LOESS) normalization [4] and between-array quantile normalization from the Limma R package for 2-channel arrays. All resulting gene expression values are in logged form. Normalization scripts for each breast cancer dataset are available at the Github repository https://github.com/kplaney/curatedBreastCancer. There were no labeled batches within a single study, but samples collected on different platforms within the same study were normalized as separate datasets. If the raw datasets were not available, only datasets with similar normalization schemes as previously described were included in the analysis. For duplicated gene symbols linked to different probes in an array, the probe linked to that specific gene with the highest variance across all samples was kept. Samples from the same patient were filtered in a similar fashion to keep only one microarray per unique patient identifier. Missing values were then imputed via KNN-impute [5]. Genes with a variance below 0.001 within a specific dataset were removed from that dataset.

The ovarian datasets were processed in a similar manner as the breast cancer datasets, but by the curatedOvarianData R package authors [6]. The ovarian TCGA dataset contained several batches. To test for existence of a batch effect, the first principal component for each sample, across all genes with minimum variance of 0.001 across all samples, was used as the response variable in a linear regression analysis. The independent variable in the linear regression was the batch number; a significant p-value for the dataset number variable coefficient indicates a strong dataset-specific signal across datasets. The p-value for this analysis was below 6.13E-05, indicating that batch-specific signal (and/or noise)

did exist. We thus applied the ComBat batch correction technique from the R sva package [7]. The TCGA dataset contained only serous samples, minimizing the chance that a batch correction technique would smooth out key signaling patterns between highly distinct patient sample microarrays. The effect of ComBat on consensus clustering assignments as opposed to the clustering assignments found by the original ovarian TCGA paper [8] was minimal; the changes in membership can be seen in Figure S20 in Additional File 1.

Selection of de novo single-dataset clustering method

Clustering methods were evaluated using the breast cancer datasets with the restricted and unrestricted PAM50 gene sets. The baseline clustering methods Hartigan Wong's k-means [9] and hierarchical clustering with a Euclidean distance matrix and average linkage were tested, along with the consensus clustering [10] forms of these methods. The Gap Test [11] was used to select the number of clusters for the baseline clustering methods and four methods were tested to select the number of clusters for the consensus clusterings: the Proportion of Ambiguous Clusters (PAC) score [12], the PAC score rounded to the nearest hundredth, the mean consensus and the consensus fraction (described in more detail below). We used consensus clustering with from 1 to K clusters [10]. Consensus clustering produces a heatmap for each K number of clusters tested (Figure S1 in Additional File 1.)

Various methods have been published on how to select K using consensus clustering, but unlike the PAC score, many earlier methods will report greater than one cluster for randomly generated unimodal data [12]. The PAC score is calculated from the consensus cumulative distribution function (CDF) (Figure S2 in Additional File 1). A PAC score of

near zero is desirable which corresponds to a step-like CDF function; the optimal number of K clusters is the K with the lowest PAC score. All methods were tested using 500 permutation or resampling iterations. Consensus clustering was run using Bioconductor's ConsensusClusterPlus package, which implements the Monti et al. method [10]. The Gap test was run using the principal component method for creating simulated datasets [11] from the R cluster package. We used breast cancer data with immunohistochemistry (IHC) ER and HER2 status and/or commercial Pam50 platform results as established ground truth to evaluate all algorithms and determined Hartigan Wong's k-means [9] consensus clustering with 1 random start and with 90% resampling of samples for each of the 500 iterations with a rounded PAC score to be the optimal single-dataset clustering method. See Table S2 in Additional File 2 for detailed results. Thus, these settings were used for all de novo clusterings presented in this paper.

Survival Analyses

Survival analyses were conducted for the experiments using real datasets, i.e. the breast and ovarian cancer gene expression experiments. The breast cancer datasets had predominantly binary outcomes measurements. Thus, to evaluate the prognostic significance of the breast cancer subtypes CoINcIDE discovered, a generalized linear model was used to evaluate the prognostic significance of patients' meta-cluster status correlated with treatment and pathological complete response (pCR) and Relapse- or Disease-Free Survival (RFS,DFS). pCR is defined as the absence of disease upon surgical removal of a tumor if a tumor was given pre-surgery (neoadjuvant) treatment. Residual Cancer Burden (RCB) was not measured in enough of the datasets to be used in survival analyses [13]. RFS was defined as any recurrence of the disease, local or

metastatic, but does not include death due to the disease, while DFS includes both recurrence and death due to the disease [14].

Only binary values were available for these three variables for the majority of the breast cancer datasets, and thus continuous survival analyses were not conducted. We ensured by reading the original publication attached to each dataset that the RFS and DFS values were calculated using patients that were followed for a minimum of 3 years (most were followed at least 5 years.)

Survival analyses were also conducted for three supervised analyses by using the PAM50 centroid classification before any transformation, after gene-wise Batch Mean Centering (BMC) and after ComBat (i.e. a patient's PAM50 classification was used as its subtype.) Finally, survival analyses were also conducted on the subtypes derived from consensus clustering the concatenated datasets using first non-transformed data, then using BMC and then using ComBat.

A chi-squared contrast test was used to calculate a p-value quantifying the significance in improved outcomes predictions when patient meta-clusters status was added to a baseline logistic regression model with only patients' treatment information. Three binary treatment variables used indicated whether a patient had chemotherapy, an anti-ER (estrogen) therapy and/or an anti-HER2 therapy. The AUC for each combined treatment information plus meta-cluster status model was derived from ROC curves using the R pROC package. Table S8 summarizes these results for all breast cancer analyses.

Continuous right censored survival times were available in the majority of the ovarian cancer datasets, and thus Cox proportional hazards models from the survival R package were used to determine if the CoINcIDE subtype status for each ovarian patient

significantly stratified patients by overall survival [15]. We used Kaplan-Meier curves in combination with the log rank test to visualize survival analysis between CoINcIDE subtypes. The ovarian datasets did not have consistent treatment information, and thus this was not included in any of the survival analyses.

Meta effect size analysis for gene expression subtypes

A supervised gene meta-analysis of the clusters within each CoINcIDE subtype/meta-cluster was conducted to determine if certain genes are significantly under or overexpressed within a certain meta-cluster. The supervised labels were the meta-cluster membership of each sample: for each meta-cluster analysis, samples in cluster A that are in a certain meta-cluster are assigned a label of 1. Samples within the same dataset as cluster A, but not within cluster A, are assigned a label of 0. Then, within each CoINcIDE meta-cluster, for each individual cluster, the effect size of each gene was computed using Hedge's g effect size [16]. The effect size for this gene across all clusters in a meta-cluster was summarized via a weighted average; effect sizes for each cluster were weighted inversely by the standard deviation of the gene expression values for that gene in that specific cluster. If a dataset clustering run created two very similar clusters that were presumably too granular, they may be included in the same meta-cluster. In this case, CoINcIDE treats these two clusters from the same dataset as one cluster. Finally, genes within a CoINcIDE subtype that passed a minimum summary effect size threshold of 0.5 and existed in the Druggable Genome [17] were reported to provide potentially novel druggable hypothesis for each subtype.

Functional Enrichment Analysis

Genes within a CoINcIDE subtype that passed a minimum summary effect size threshold

of 0.5 were selected for further analysis to specifically explore gene overexpression patterns. These genes are used as inputs for a functional enrichment analysis. The Broad Institute databases were used as reference gene lists [18] including the C4/CGN Cancer Gene Neighborhoods, C4/CM Cancer modules, C6 Oncogenic signatures, C7/Immunological Signatures and C3/TFT Transcription Factor Targets databases. Next, the hypergeometric test was used on the filtered effect size-derived gene lists for each CoINcIDE subtype to determine which reference gene lists were enriched for that specific meta-cluster [19]. The total number of background genes for each experiment was computed by taking the union of all unique genes across all reference gene lists and the input gene list. All final gene list p-values were FDR corrected [20] to account for the total number of reference gene lists being tested. Gene lists with q-values of less than or equal to 0.05 that were unique to a specific CoINcIDE subtype and not shared between subtypes were reported.

Batch Transformation Details

For gene-wise batch mean centering, within each dataset, the mean of each gene across all samples was removed from each sample-gene value. ComBat was run using the R sva package. The same batch detection method detailed in the above ovarian cancer dataset processing section was employed to test for dataset-specific effects. The first principal component for each sample, across all 35 PAM50 restricted genes, was used as the response variable in a linear regression analysis. The independent variable in the linear regression was the dataset number; the p-value for this analysis was 0.055, indicating that dataset-specific signal (and/or noise) did exist.

1. Bhattacharjee A, Richards WG, Staunton J, Li C, Monti S, Vasa P, Ladd C, Beheshti J, Bueno R, Gillette M, Loda M, Weber G, Mark EJ, Lander ES, Wong W, Johnson BE, Golub TR, Sugarbaker DJ, Meyerson M: **Classification of human lung carcinomas by mRNA expression profiling reveals distinct adenocarcinoma subclasses.** *Proc Natl Acad Sci U S A* 2001, **98**:13790–5.

2. Flury B: *A First Course in Multivariate Statistics*. New York: Springer-Verlag; 1997.

3. Wu Z, Irizarry R, Gentleman R, Murillo FM, Spencer F: **A Model Based Background Adjustment for Oligonucleotide Expression Arrays**. *Journal of the American Statistical Association* 2004:909–917.

4. Cleveland WS, Devlin SJ: **Locally Weighted Regression: An Approach to Regression Analysis by Local Fitting**. *J Am Stat Assoc* 1988, **83**:596–610.

5. Troyanskaya O, Cantor M, Sherlock G, Brown P, Hastie T, Tibshirani R, Botstein D, Altman RB: **Missing value estimation methods for DNA microarrays.** *Bioinformatics* 2001, **17**:520–5.

6. Ganzfried BF, Riester M, Haibe-Kains B, Risch T, Tyekucheva S, Jazic I, Wang XV, Ahmadifar M, Birrer MJ, Parmigiani G, Huttenhower C, Waldron L: **curatedOvarianData: clinically annotated data for the ovarian cancer transcriptome.** *Database (Oxford)* 2013, **2013**:bat013.

7. Johnson WE, Li C, Rabinovic A: **Adjusting batch effects in microarray expression data using empirical Bayes methods.** *Biostatistics* 2007, **8**:118–27.

8. Cancer T, Atlas G: **Integrated genomic analyses of ovarian carcinoma.** *Nature* 2011, **474**:609–15.

9. Hartigan J: *Clustering Algorithms.* John Wiley & Sons, Inc.; 1975.

10. Monti S, Tamayo P, Mesirov J, Golub T: **Consensus Clustering: A Resampling-Based Method for Class Discovery and Visualization of Gene Expression Microarray Data**. *Mach Learn* 2003, **52**:91–118.

11. Tibshirani R, Walther G, Hastie T: **Estimating the number of clusters in a data set via the gap statistic**. *J R Stat Soc* 2000, **63**:411–423.

12. Şenbabaoğlu Y, Michailidis G, Li JZ: **Critical limitations of consensus clustering in class discovery.** *Sci Rep* 2014, **4**:6207.

13. Symmans WF, Peintinger F, Hatzis C, Rajan R, Kuerer H, Valero V, Assad L, Poniecka A, Hennessy B, Green M, Buzdar AU, Singletary SE, Hortobagyi GN, Pusztai L: **Measurement of residual breast cancer burden to predict survival after neoadjuvant chemotherapy.** *J Clin Oncol* 2007, **25**:4414–22.

14. Chua YJ, Sargent D, Cunningham D: **Definition of disease-free survival: this is my truth-show me yours.** *Ann Oncol* 2005, **16**:1719–21.

15. Elandt-Johnson RC, Johnson NL: *Survival Models and Data Analysis*. John Wiley & Sons; 1999.

16. Hedges L V.: **Distribution Theory for Glass's Estimator of Effect size and**

Related Estimators. *J Educ Behav Stat* 1981, **6**:107–128.

17. Hopkins AL, Groom CR: **The druggable genome.** *Nat Rev Drug Discov* 2002, **1**:727–30.

18. **Broad Institute MSigDB Collections** [http://www.broadinstitute.org/gsea/msigdb/collections.jsp]

19. Rivals I, Personnaz L, Taing L, Potier M-C: **Enrichment or depletion of a GO category within a class of genes: which test?** *Bioinformatics* 2007, **23**:401–7.

20. Benjamini, Y., Hochberg Y: **Controlling the false discovery rate: a practice and powerful approach to multiple testing**. *J R Stat Soc Ser B (Statistical Methodol* 1995, **57**:289–300.

1. Bhattacharjee A, Richards WG, Staunton J, Li C, Monti S, Vasa P, Ladd C, Beheshti J, Bueno R, Gillette M, Loda M, Weber G, Mark EJ, Lander ES, Wong W, Johnson BE, Golub TR, Sugarbaker DJ, Meyerson M: **Classification of human lung carcinomas by mRNA expression profiling reveals distinct adenocarcinoma subclasses.** *Proc Natl Acad Sci U S A* 2001, **98**:13790–5.

2. Flury B: *A First Course in Multivariate Statistics*. New York: Springer-Verlag; 1997.

3. Wu Z, Irizarry R, Gentleman R, Murillo FM, Spencer F: **A Model Based Background Adjustment for Oligonucleotide Expression Arrays**. *Journal of the American Statistical Association* 2004:909–917.

4. Cleveland WS, Devlin SJ: **Locally Weighted Regression: An Approach to Regression Analysis by Local Fitting.** *J Am Stat Assoc* 1988, **83**:596–610.

5. Troyanskaya O, Cantor M, Sherlock G, Brown P, Hastie T, Tibshirani R, Botstein D, Altman RB: **Missing value estimation methods for DNA microarrays.** *Bioinformatics* 2001, **17**:520–5.

6. Ganzfried BF, Riester M, Haibe-Kains B, Risch T, Tyekucheva S, Jazic I, Wang XV, Ahmadifar M, Birrer MJ, Parmigiani G, Huttenhower C, Waldron L: **curatedOvarianData: clinically annotated data for the ovarian cancer transcriptome.** *Database (Oxford)* 2013, **2013**:bat013.

7. Johnson WE, Li C, Rabinovic A: **Adjusting batch effects in microarray expression data using empirical Bayes methods.** *Biostatistics* 2007, **8**:118–27.

8. Cancer T, Atlas G: **Integrated genomic analyses of ovarian carcinoma.** *Nature* 2011, **474**:609–15.

9. Hartigan J: *Clustering Algorithms.* John Wiley & Sons, Inc.; 1975.

10. Monti S, Tamayo P, Mesirov J, Golub T: **Consensus Clustering: A Resampling-Based Method for Class Discovery and Visualization of Gene Expression Microarray Data**. *Mach Learn* 2003, **52**:91–118.

11. Tibshirani R, Walther G, Hastie T: **Estimating the number of clusters in a data set via the gap statistic**. *J R Stat Soc* 2000, **63**:411–423.

12. Şenbabaoğlu Y, Michailidis G, Li JZ: **Critical limitations of consensus clustering in class discovery.** *Sci Rep* 2014, **4**:6207.

13. Symmans WF, Peintinger F, Hatzis C, Rajan R, Kuerer H, Valero V, Assad L, Poniecka A, Hennessy B, Green M, Buzdar AU, Singletary SE, Hortobagyi GN, Pusztai L: **Measurement of residual breast cancer burden to predict survival after neoadjuvant chemotherapy.** *J Clin Oncol* 2007, **25**:4414–22.

14. Chua YJ, Sargent D, Cunningham D: **Definition of disease-free survival: this is my truth-show me yours.** *Ann Oncol* 2005, **16**:1719–21.

15. Elandt-Johnson RC, Johnson NL: *Survival Models and Data Analysis*. John Wiley & Sons; 1999.

16. Hedges L V.: **Distribution Theory for Glass's Estimator of Effect size and Related Estimators**. *J Educ Behav Stat* 1981, **6**:107–128.

17. Hopkins AL, Groom CR: **The druggable genome.** *Nat Rev Drug Discov* 2002, **1**:727–30.

18. **Broad Institute MSigDB Collections** [http://www.broadinstitute.org/gsea/msigdb/collections.jsp]

19. Rivals I, Personnaz L, Taing L, Potier M-C: **Enrichment or depletion of a GO category within a class of genes: which test?** *Bioinformatics* 2007, **23**:401–7.

20. Benjamini, Y., Hochberg Y: **Controlling the false discovery rate: a practice and powerful approach to multiple testing.** *J R Stat Soc Ser B (Statistical Methodol* 1995, **57**:289–300.
