# Supplementary material for: CoINcIDE: A framework for discovery of patient subtypes across multiple datasets
Source: Genome Med. 2016 Mar 9;8:27. doi: 10.1186/s13073-016-0281-4 (PMC4784276; doi:10.1186/s13073-016-0281-4)

Additional file 3

Supplementary Figures

Supplementary Figure 1: An example consensus heatmap of the clustered consensus matrix using breast cancer dataset GSE2034 using k-means consensus clustering with the restricted PAM50 gene with K=3 clusters. The dendogram represents the final patient clusters as determined by hierarchical clustering with average linkage applied to the consensus matrix (the 500 clustering iterations that produced this consensus matrix were run using the CoINcIDE single-dataset clustering algorithm, k-means consensus clustering.) The solid blue color along the diagonal represents consensus matrix values close to 1, or patient pairs who were placed in the same cluster 100% of the time. Cluster one contains patients that were sometimes assigned to cluster 2 and cluster 3, as denoted by the mixed colors in the bar below the dendogram.

Supplementary Figure 2: An example consensus cumulative distribution function (CDF) using breast cancer dataset GSE2034 using k-means consensus clustering with the restricted PAM50 gene set. The PAC score selects the number of clusters that maximizes the CDF (see Supplementary Methods in Additional File 3 for details.) Clusters 2 and 3 have clear step-like functions between the number of patient-patient membership fraction values of 0 and 1; the PAC score chose a final K=3 for this analysis.

Supplementary Figure 3: Gaussian density plots of all the Pearson's correlation metric similarities between all potential cluster-cluster pairs for the CoINcIDE (A) PAM50 centroid clustering, (B) PAM50 de novo clustering, (C) ovarian short gene list de novo clustering and (D) ovarian long gene list de novo clustering analyses. These plots were used to select the minimum mean similarity metric for each CoINcIDE analysis on real data. Thresholds of 0.15, 0.25, 0.5 and 0.5 were employed, respectively. The second peak of 0.7 in the ovarian long gene list analysis (D) was also tested.

Supplementary Figure 4: Examples of the patient-patient correlation heatmaps of datasets used in the meta-cluster simulations. (A) The true Bhattacharjee dataset with four tissue clusters used to derive simulated data. (B) A high-quality simulated dataset with four tissue clusters, each with 50 samples. (C) A mixed-quality simulated dataset with clusters one and four derived from carcinoid and small cell tissues, respectively, and clusters two and three derived from a random sampling across all tissue types, each with 50 samples. (D) A high-quality simulated dataset a random number of clusters and cluster sizes; this specific example has only two tissue clusters, the first with 190 carcinoid samples, and the second with 10 colon samples.

Supplementary Figure 5: TPR (True Positive Rate) versus FPR (False Positive Rate) plots for each simulation scenario. Each dot on each sub-figure represents a different minimum mean similarity threshold, as denoted by the color key. The x-axis, or FPR, is truncated at 0.02 because no FPR values ever reached above this threshold. The y-axis, or TPR, is plotted from 0.0 to 1.0, as this captures the full range of TPR values. Each simulation has four sub-figures to visualize the TPR-FPR points at standard deviation random normal

noise levels of 0.0, 0.8, 1.6 and 2.4 (noise levels are denoted on top of the figures inside the gray bars.) Main Figure 2 contains similar plots, but at a single noise level (0.8) with each simulation scenario set side by side. (A)-(G) visualize each simulation scenario in further detail for the four aforementioned noise levels. (A) are the results for the high quality simulation scenario with an equal cluster size and equal number of clusters, (B) results for the random cluster size and equal number of clusters, (C) results for the equal cluster size and random number of clusters with a minimum of two clusters per dataset, (D) results for the equal cluster size and random number of clusters with a minimum of one cluster per dataset, (E) results for the random cluster size and random number of clusters per dataset with a minimum of two clusters per dataset, (F) results for the random cluster size and random number of clusters per dataset with a minimum of one cluster per dataset and (G) results for the equal cluster size but 50% random/noisy clusters scenario. The seventh simulation scenario (G) contains a noticeably lower TPR rate because only 50% of the clusters were true clusters in the sense that they were designed to be similar to clusters in other datasets.

Supplementary Figure 6: Simulation True Positive Rates (TPR) for each simulation scenario with increasing noise for similarity thresholds of 0.0, 0.3, 0.5 and 0.7 (levels higher than 0.7 resulted in no clusters being assigned edges, i.e. TPR=0.) The specific similarity threshold used to make a sub-figure is denoted on the gray side bar. The noise level, denoted on the x-axis, is the standard deviation of random normal noise added to each dataset. The error bars represent the standard error over 50 simulation iterations. (A)-(G) correspond to the simulation scenarios described in the same order in the above caption for Supplementary Figure 5.

Supplementary Figure 7: Breast PAM50 supervised centroid clustering network plot with dataset labels on cluster nodes. All PAM50 genes were used when available for a specific dataset. This is the same network as shown in Figure 3B, but nodes are enlarged and not scaled by size in order to clearly display the number of the dataset each cluster came from. The dataset labels highlight potential dataset-specific biases and trends.

Supplementary Figure 8: CoINcIDE PAM50 gene set de novo clustering analysis with dataset labels. This is the same network as shown in Figure 5, but nodes are not scaled to cluster size in order to clearly print the dataset number each cluster came from on each node. All PAM50 genes were used when available for a specific dataset.

Supplementary Figure 9: CoINcIDE PAM50 intersecting network results using the 35-gene PAM50 feature set. Nodes are scaled to cluster size (number of patients). (A) is the same as the main Figure 6A; it is re-plotted here for comparison with the network in (B) and re-scaled for better visualization.

Supplementary Figure 10: Breast cancer area under the curve (AUC) and Receiver Operator Curves (ROCs) for the prediction of binary pCR, RFS and DFS using the CoINcIDE PAM50 subtypes and then the unsupervised consensus clustering subtype results of the concatenated datasets with first Batch Mean Centering (BMC) and with second ComBat (A-C, D-E, and F-H, respectively). Each patient's meta-cluster status and

treatment information was included in a logistic regression model; the response variable used is printed under each figure letter. N= denotes the number of samples used to calculate the AUC. The BMC pCR model resulted in only one of the two subtypes having patients with pCR recorded, so a model incorporating differential subtype status could not be run, and thus no plot was made. (I)-(Q) summarize AUC and ROCs for supervised prediction analyses of pCR, RFS and DFS using the concatenated datasets and assigning each sample to its PAM50 centroid with no extra transformations beyond baseline dataset-specific normalization, with BMC and with ComBat (I-K, L-N, and O-Q, respectively). (A)-(Q) used the intersecting PAM50 gene set of 35 genes that was present in all datasets. Finally, CoINcIDE AUC and ROC statistics were also computed for the 50-gene, 264-gene and 2020-gene analyses (R-T, U-W and X-Z, respectively) where the genes were chosen using the meta-rank gene method (see Supplementary Methods in Additional File 3) and included no PAM50 genes.

Supplementary Figure 11: Batch mean centering and combat transformations' alterations of PAM50 subtype assignments. Breakdown of patient PAM50 centroid classifications before and after batch mean centering transformation (A) and ComBat transformation (B). The 'before' denotes the baseline normalization for each dataset. The number of meta-clusters and sample meta-cluster assignments were made after these transformations for both (A) and (B). See Figure 6B-D for a comparison of how the meta-cluster assignments, as opposed to the PAM50 classifications, changed before and after these transformations.

Supplementary Figure 12: CoINcIDE PAM50 gene set de novo network results using the full PAM50 feature set after gene-wise Batch Mean Centering was applied to each dataset. Nodes are scaled to cluster size (number of patients). (A) is the breakdown of patients within each subtype by their PAM50 classification (B) is the final CoINcIDE network with nodes (clusters) scaled by the number of patients in each cluster.

Supplementary Figure 13: Results for CoINcIDE analyses using the meta-rank gene sets of 50, 264 and 2020 genes that contained no PAM50 genes. (A), (C) and (E) summarize the CoINcIDE subtypes by their PAM50 patient classification make-up for the 50, 264 and 2020-gene analyses that contained no PAM50 genes, respectively. (B), (D) and (F) visualize the final networks (in the same respective order for each gene set) where the nodes (clusters) are unscaled with the dataset number printed on it that each cluster came from.

Supplementary Figure 14: The patient tumor histology type breakdowns by CoINcIDE subtype for the (A) short and (B) long gene list analyses.

Supplementary Figure 15: Ovarian (A) short and (B) long gene list networks. Nodes (clusters) are not scaled to size to better visualize the dataset label on each node to infer dataset-level trends. The TCGA dataset = dataset # 24 in (A) and #23 in (B), because one less dataset was used in the large gene set analysis (B) due to a low intersection of features, causing the dataset numbers to be re-numbered.

Supplementary Figure 16: Long gene list ovarian subtype breakdowns by tumor stage. Ovarian meta-clusters 1, 2 and 5are the predominantly serous meta-clusters. Gray = NA (not measured) for a patient sample. The y-axis denotes the number of samples.

Supplementary Figure 17: Long gene list ovarian network using 0.7 similarity threshold. A minimum mean similarity metric threshold of 0.7 was used to create this network, as opposed to the 0.5 threshold used earlier, because there were density peaks at both 0.5 and 0.7 (Figure S3D). The predominantly serous meta-clusters that closely match the serous meta-clusters in Figure 7C are meta-clusters 2, 3 and 4. (A) Breakdown of patient tumor histology type by CoINcIDE subtype. (B) Final meta-cluster network with nodes (clusters) scaled to cluster size. The TCGA dataset = dataset #23.

Supplementary Figure 18: Long gene list ovarian subtypes Kaplan Meier 5-year cutoff survival curves using 0.7 similarity threshold. A minimum mean similarity metric threshold of 0.7 was used to create this network, as opposed to the 0.5 threshold used in Figure 7D. The log-rank test p-value was 3.46E-04 (and 2.52E-04 for overall survival).

Supplementary Figure 19: Long gene list ovarian subtypes with (A) and without (B) the TCGA dataset. The numbers on the nodes are the dataset number; the TCGA dataset is #23. The meta-cluster numbers in the legend pertain to the original meta-cluster numbers from the entire serous and non-serous network in Figure 7C. The black circles are laid over the three TCGA serous meta-cluster nodes for clearer identification. The edges in both (A) and (B) are weighted by their mean similarity.

Supplementary Figure 20: Ovarian TCGA cluster membership. cKmeans 200 and cKmeans are the short (240) and long (2014) gene list ovarian k-means consensus clustering used in this publication; cNMF and cHclust are the TCGA ovarian clustering reports using Non-Negative Matrix Factorization (NMF) and consensus hierarchical clustering, respectively (reports were downloaded from http://gdac.broadinstitute.org/ using the dois doi:10.7908/C1BR8R71 and doi:10.7908/C17080H0). Each clustering analysis produced three clusters; each column is a patient and the colors designate membership in one of the three clusters. Vertical changes in color indicate that a patient was not placed in the same cluster for all clustering analyses.

Supplementary Figure 1

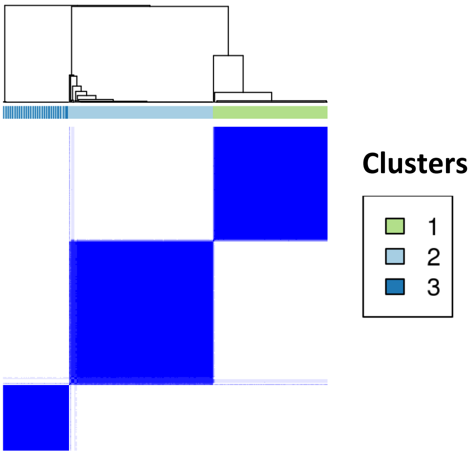

Supplementary Figure 2

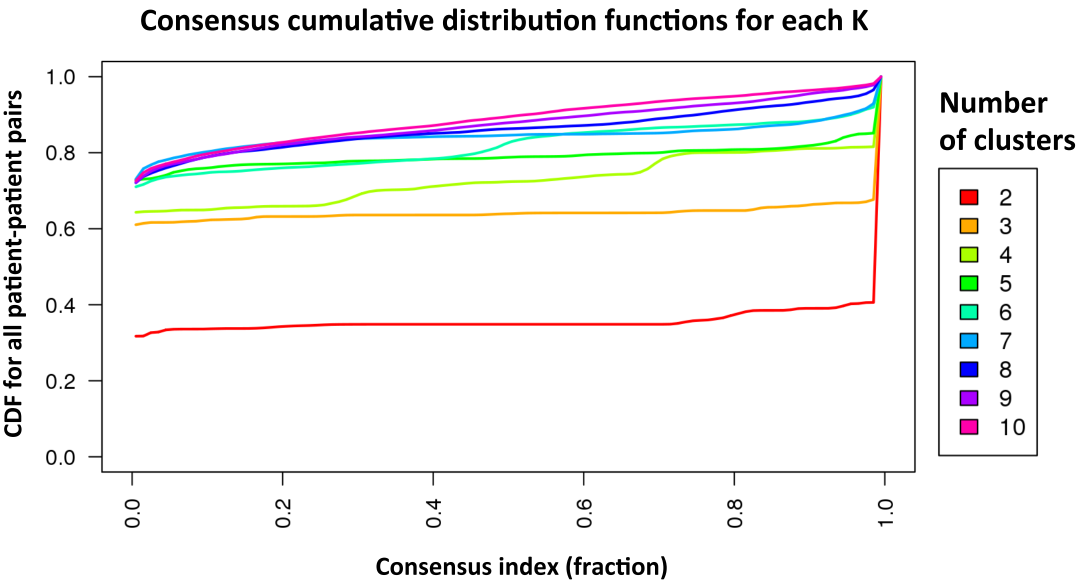

Supplementary Figure 3

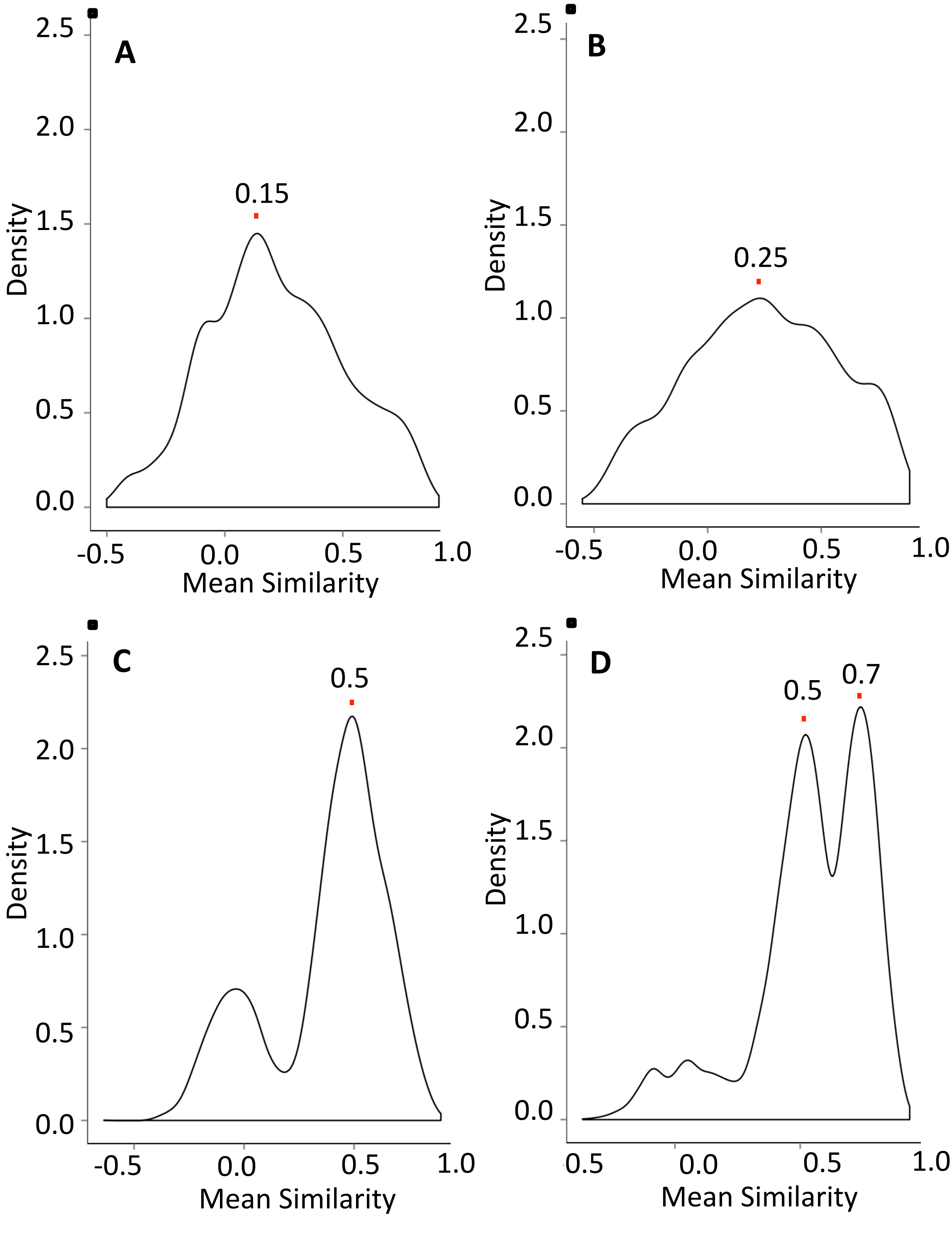

Supplementary Figure 4

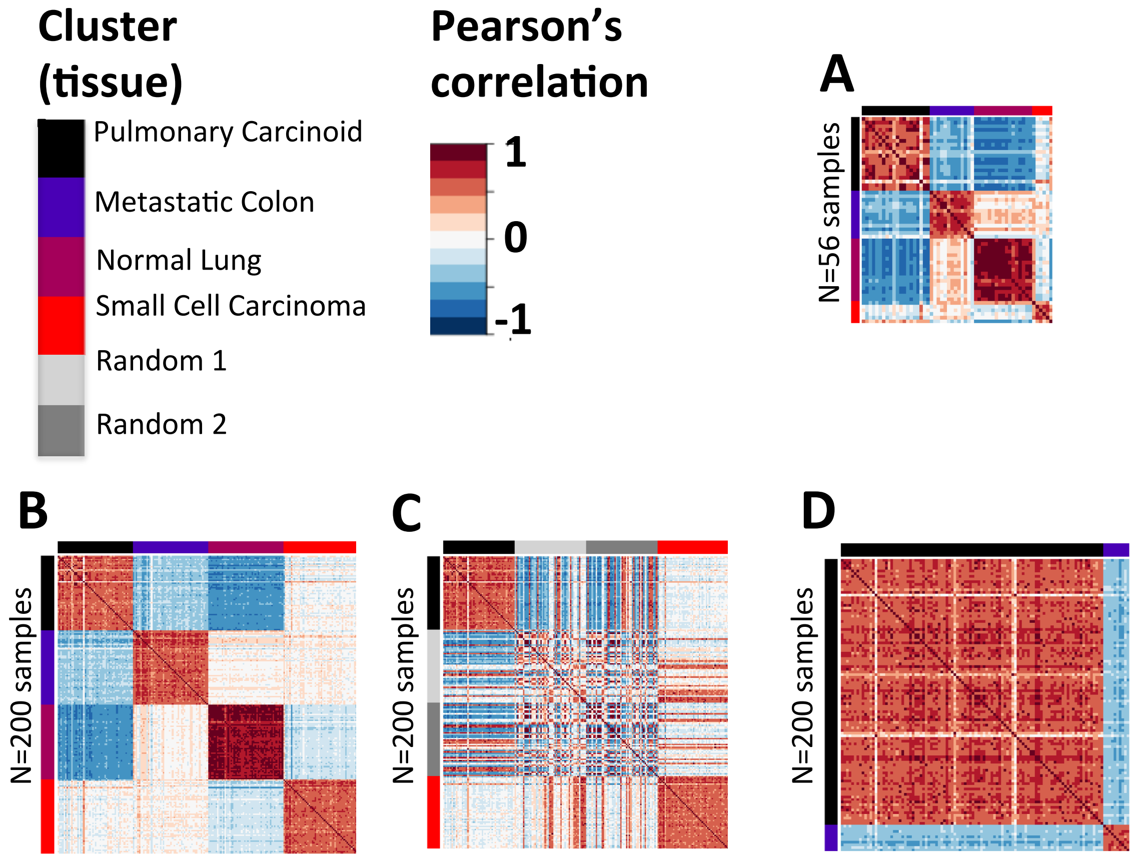

Supplementary Figure 5A (equal cluster size + equal # clusters)

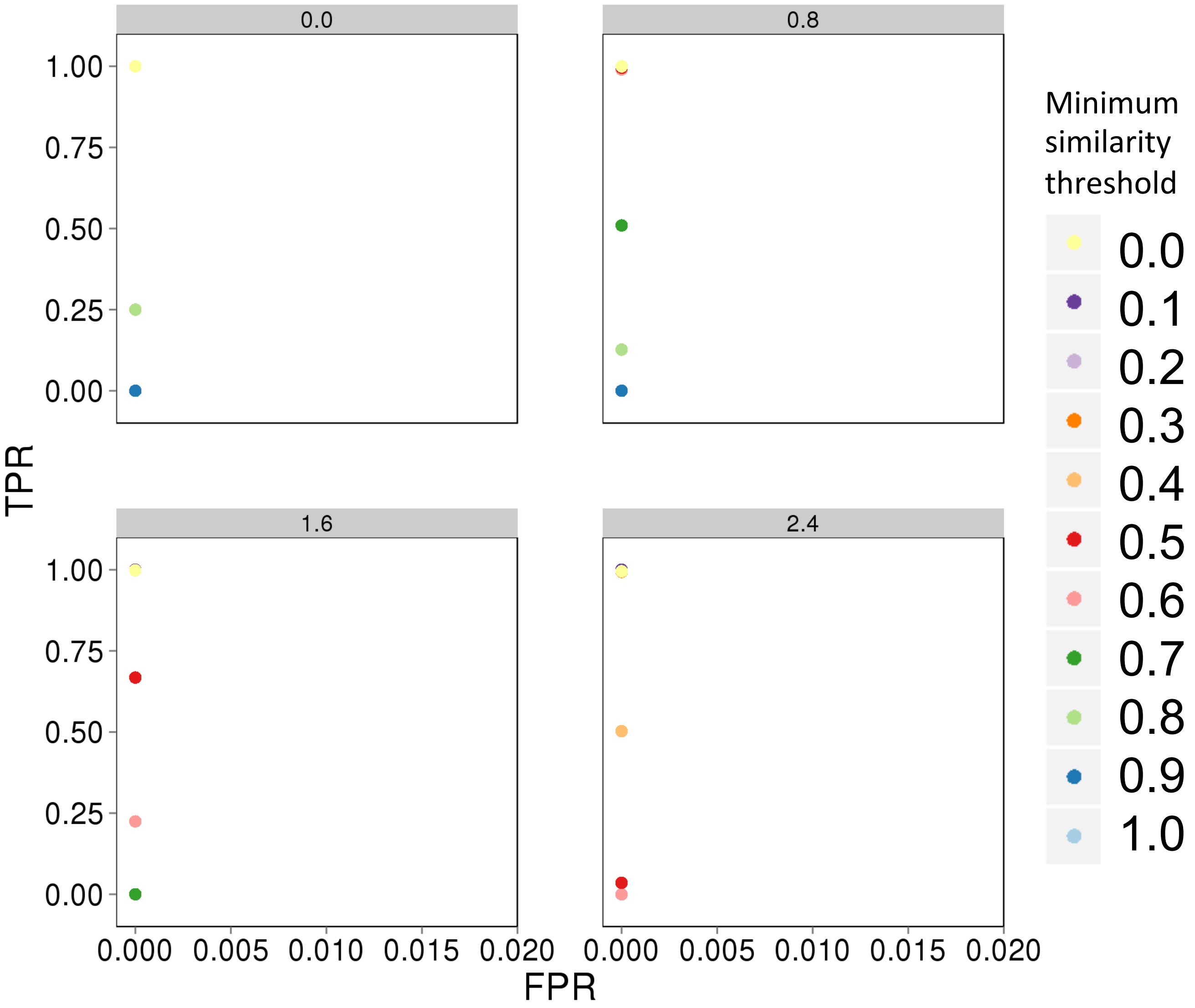

Supplementary Figure 5B (random cluster size + equal # clusters)

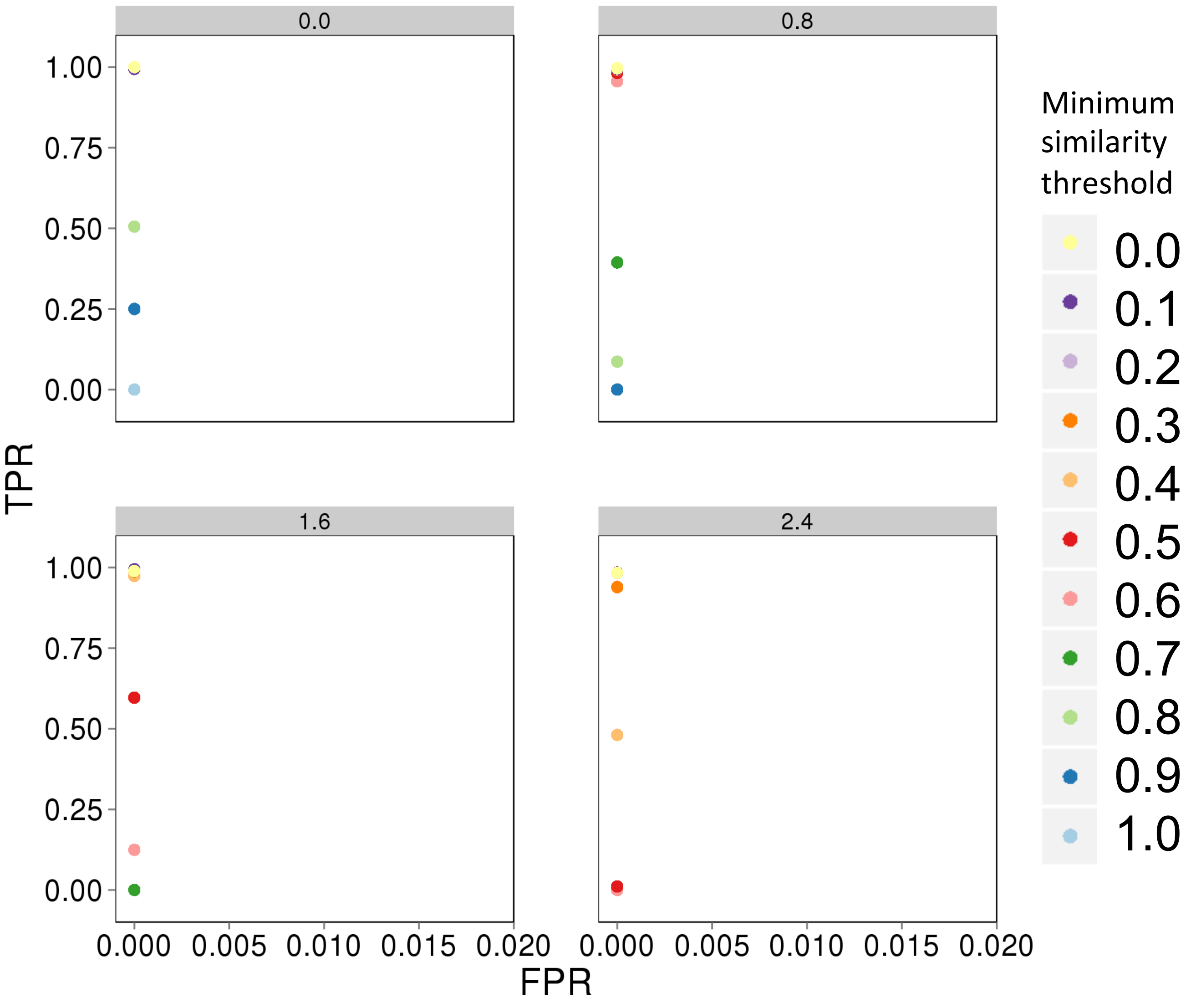

Supplementary Figure 5C (equal cluster size + unequal # clusters (2-4))

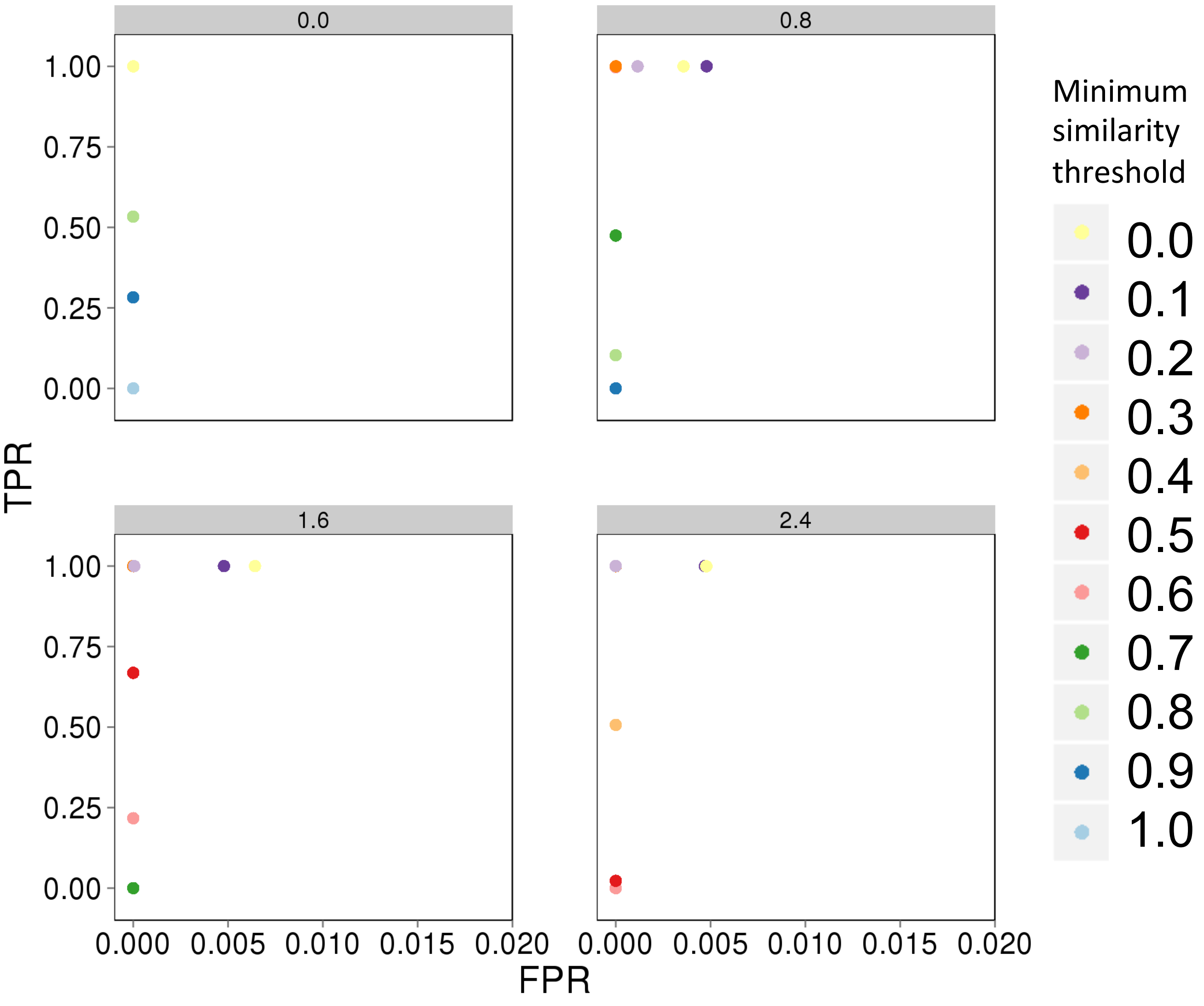

Supplementary Figure 5D (equal cluster size + unequal # clusters (1-4))

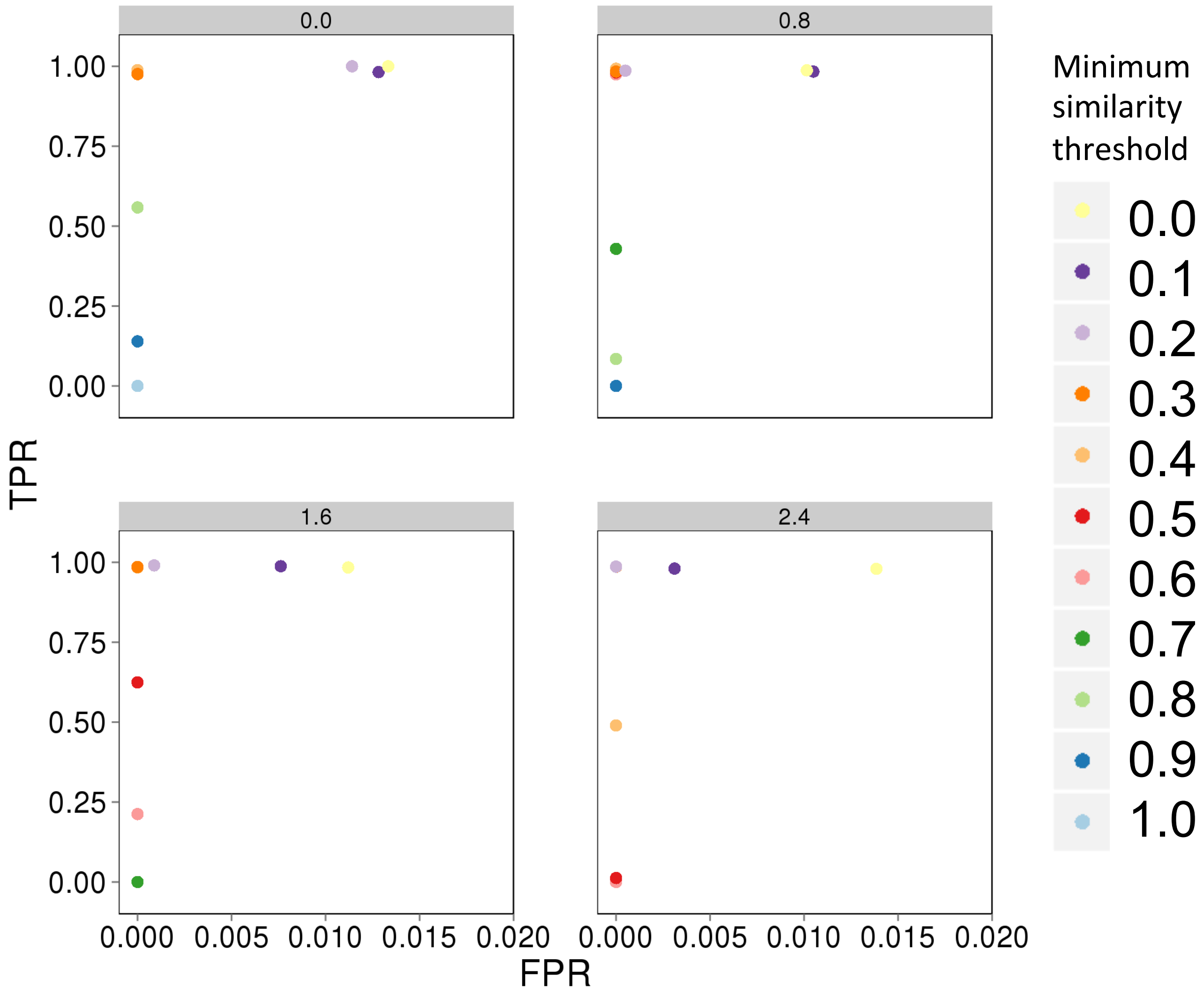

Supplementary Figure 5E (unequal cluster size + unequal # clusters (2-4))

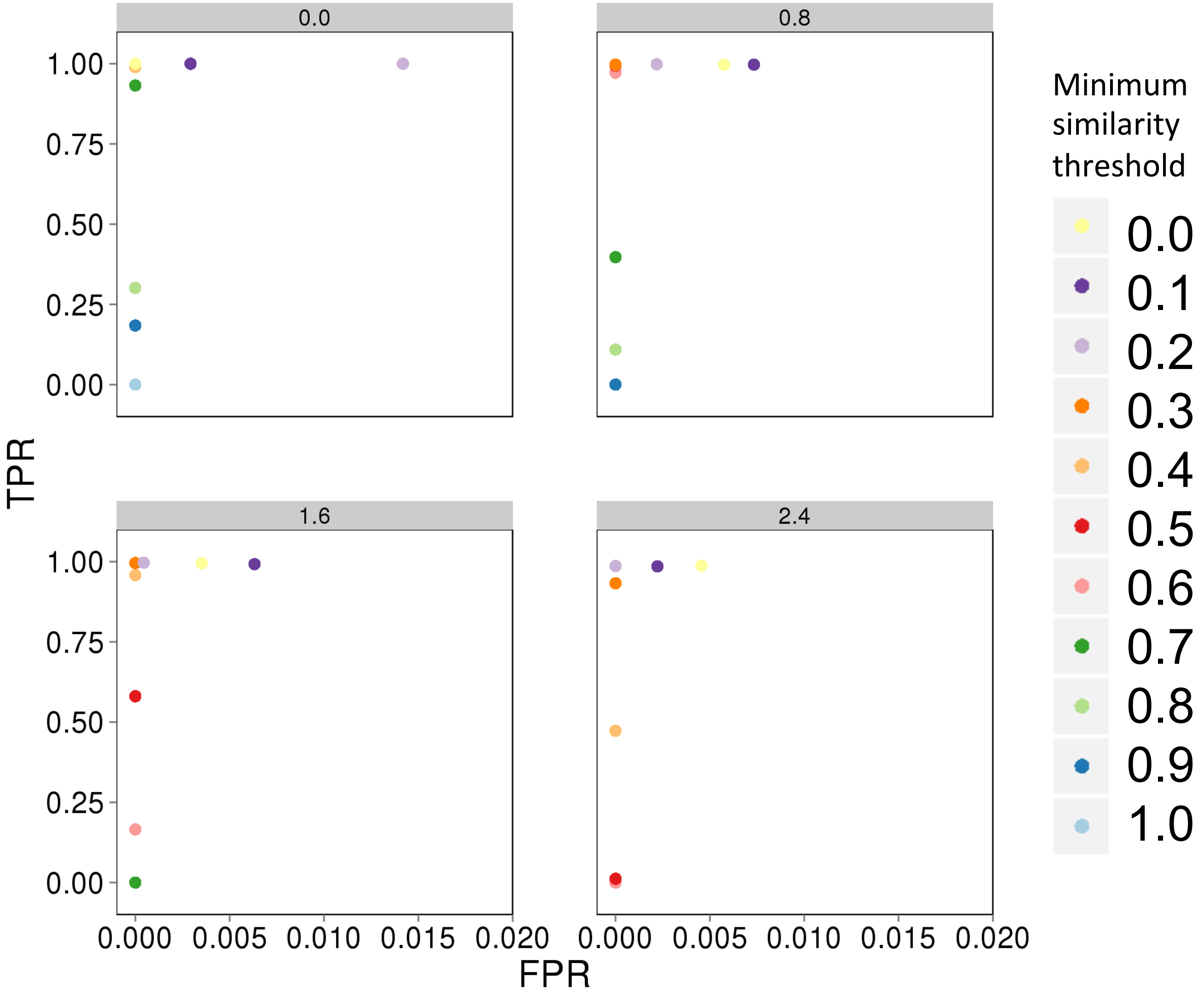

Supplementary Figure 5F (unequal cluster size + unequal # clusters (1-4))

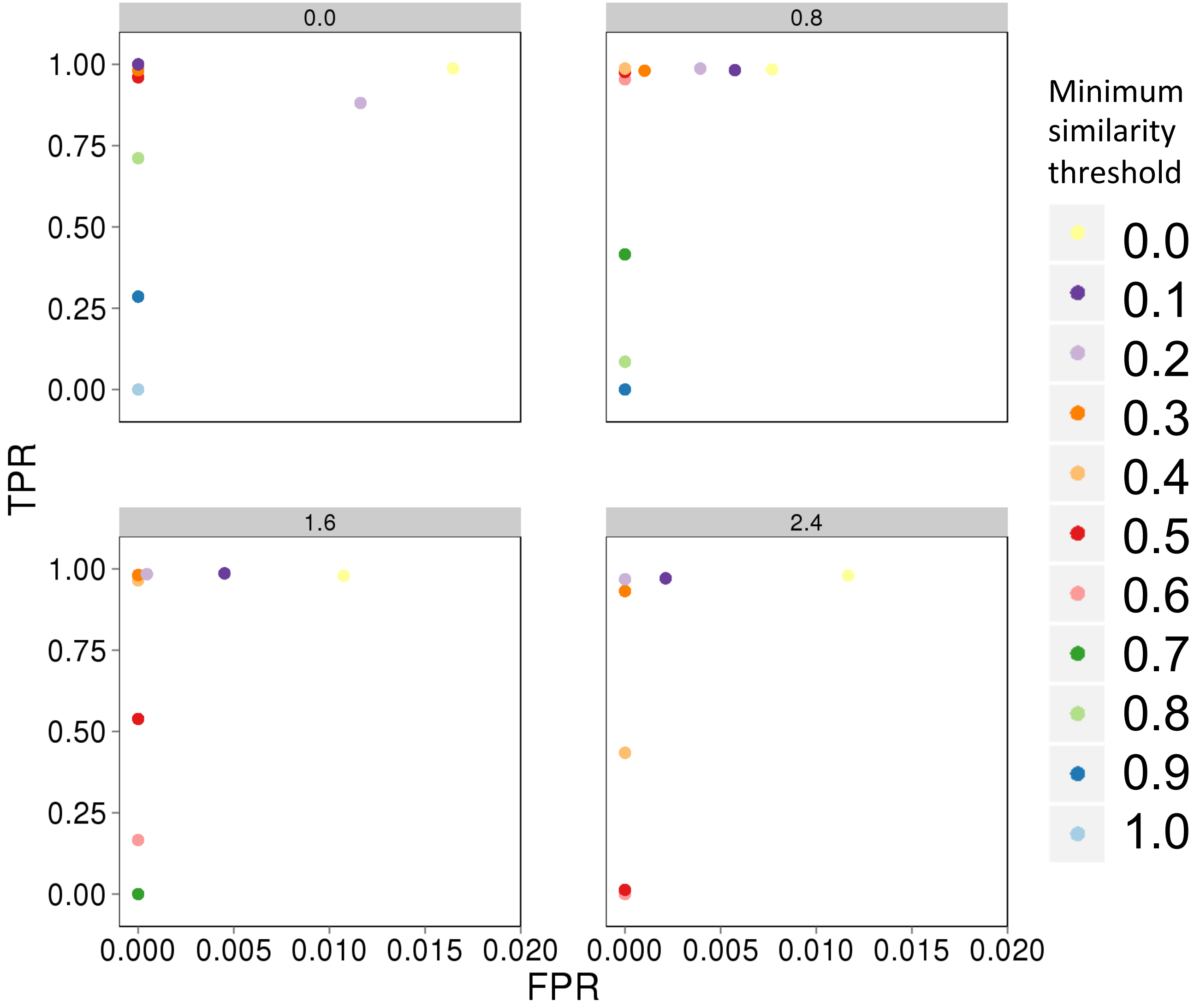

Supplementary Figure 5G (50% high quality, 50% random noise clusters)

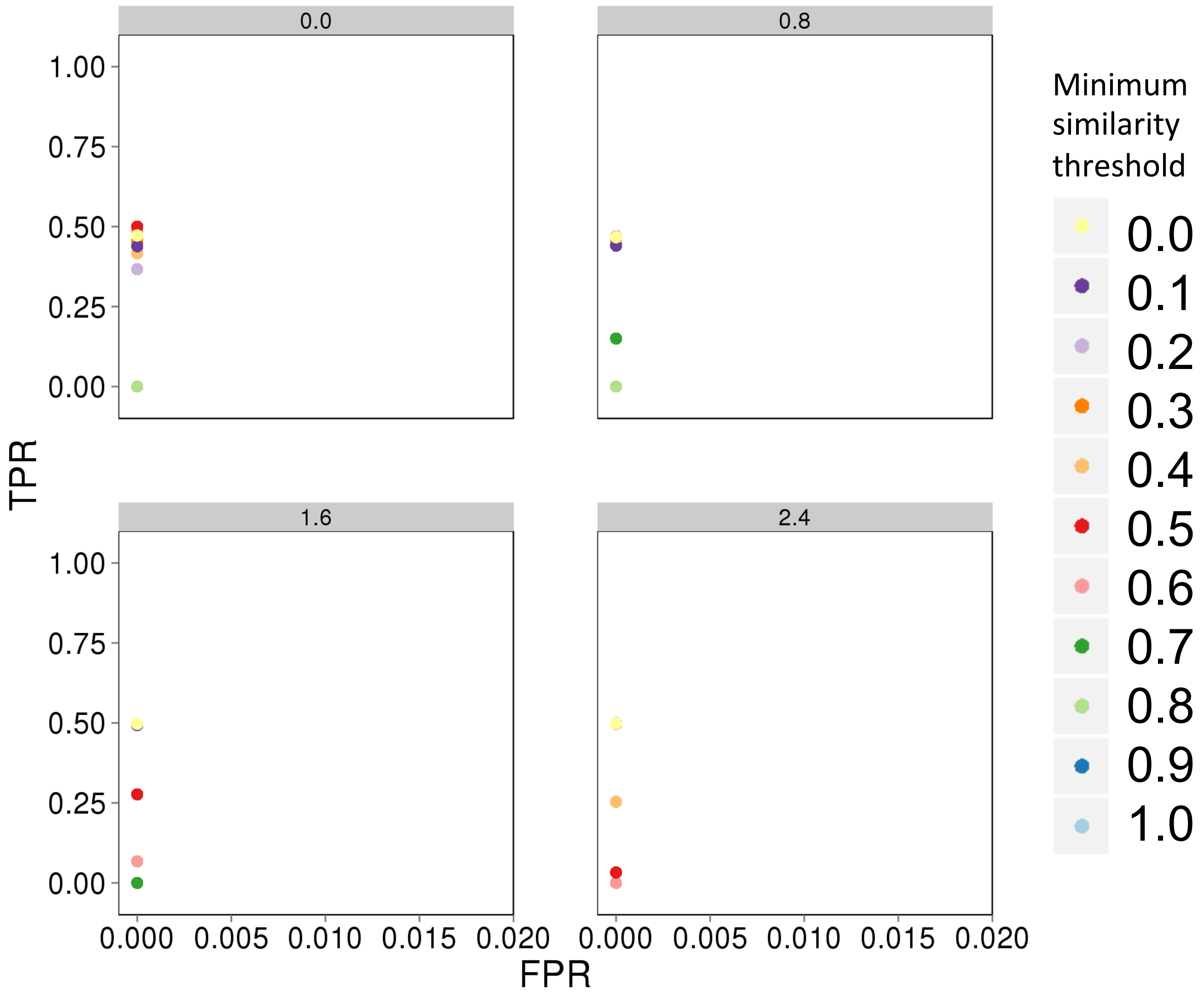

Supplementary Figure 6A (equal cluster size + equal # clusters)

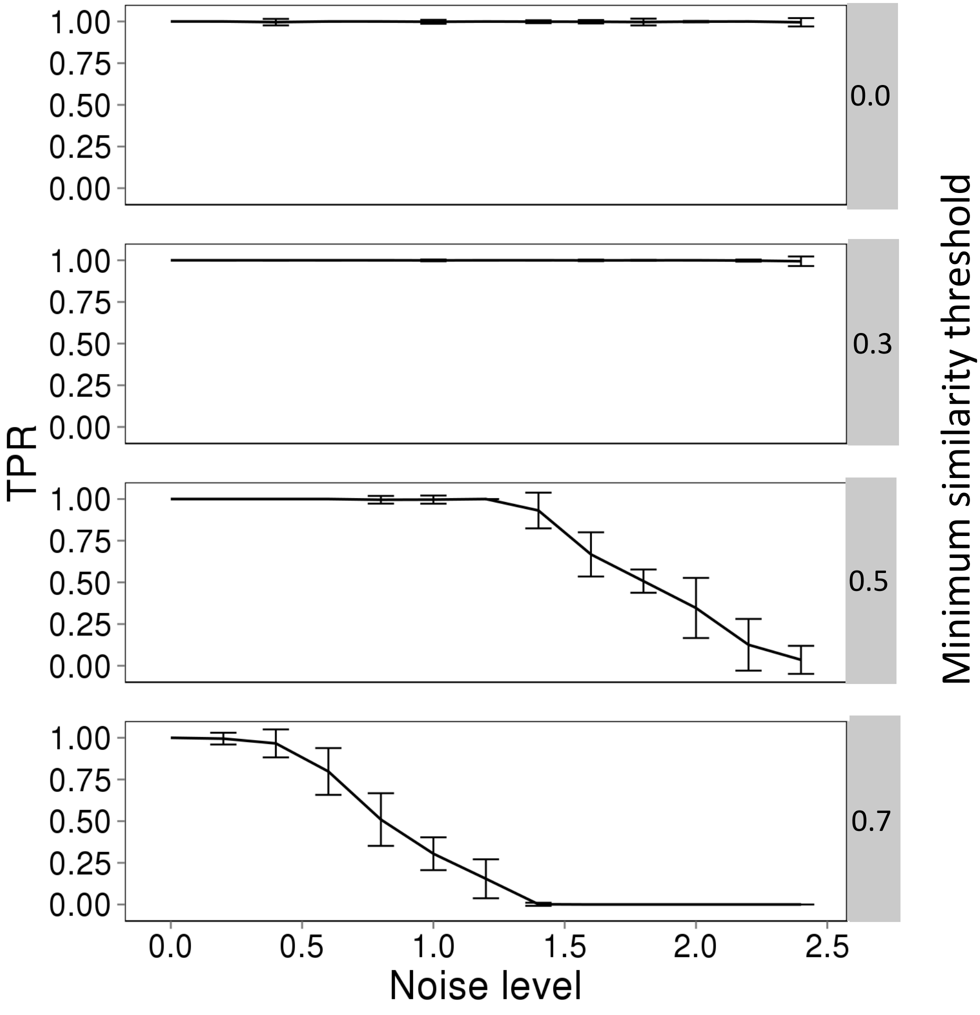

Supplementary Figure 6B (random cluster size + equal # clusters)

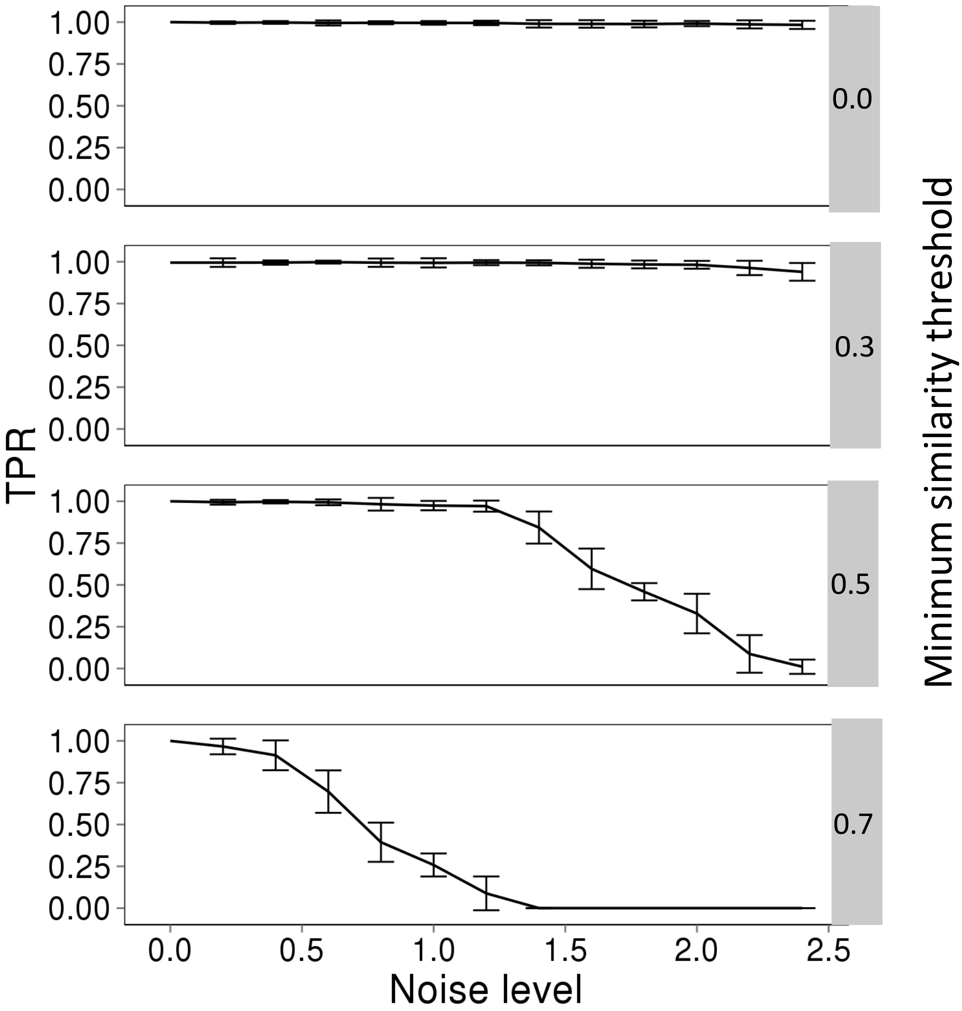

Supplementary Figure 6C
(equal cluster size + unequal # clusters (2-4))

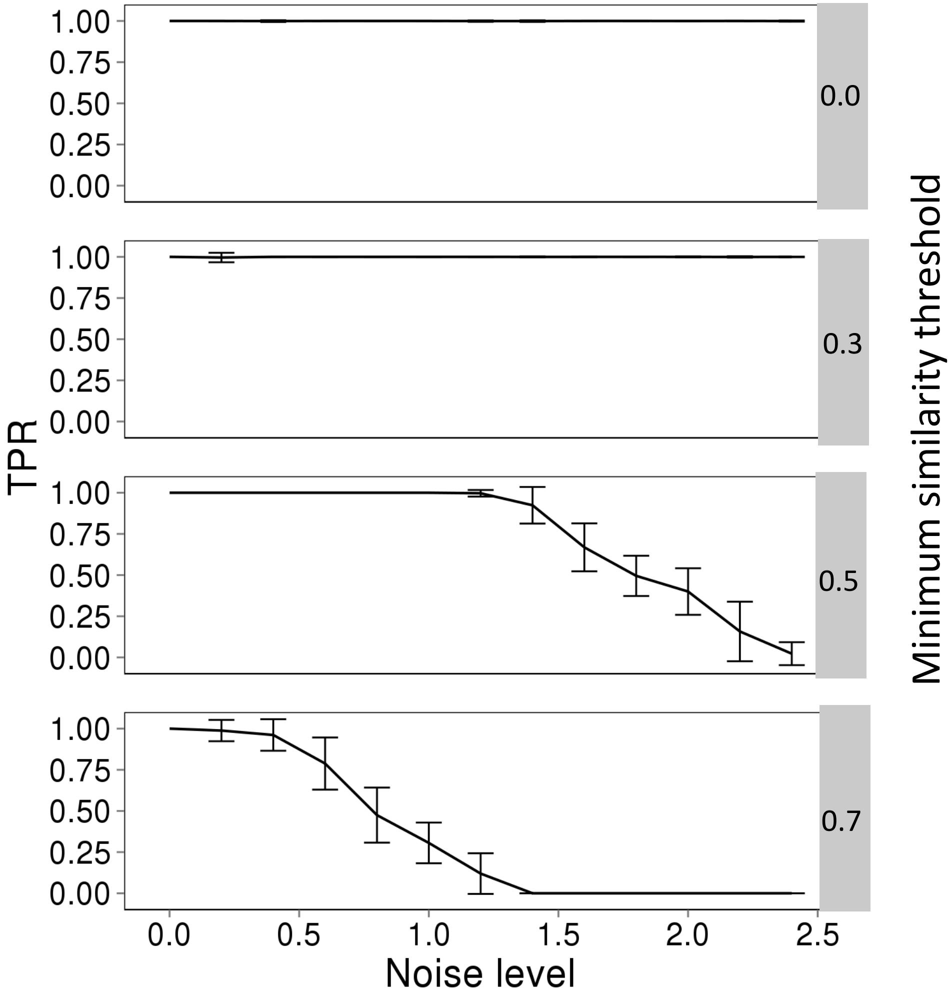

Supplementary Figure 6D
(equal cluster size + unequal # clusters (1-4))

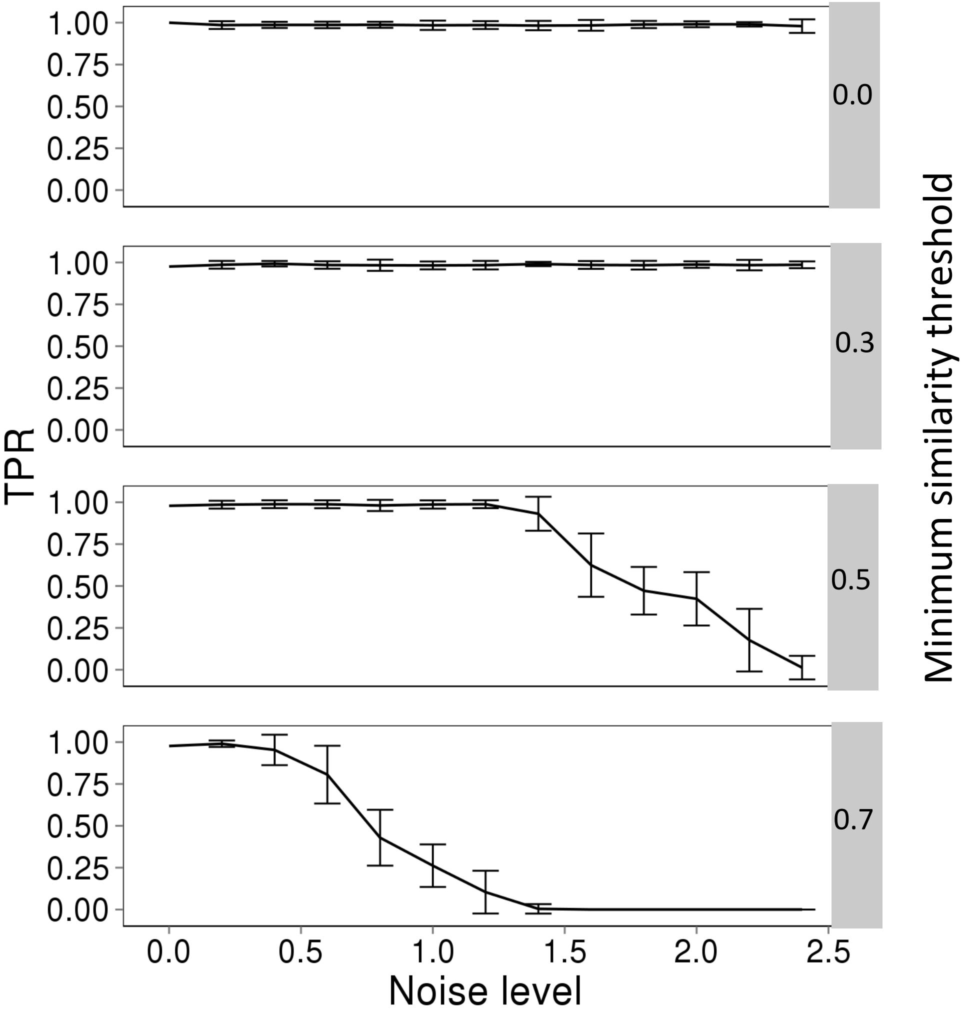

Supplementary Figure 6E
(unequal cluster size + unequal # clusters (2-4))

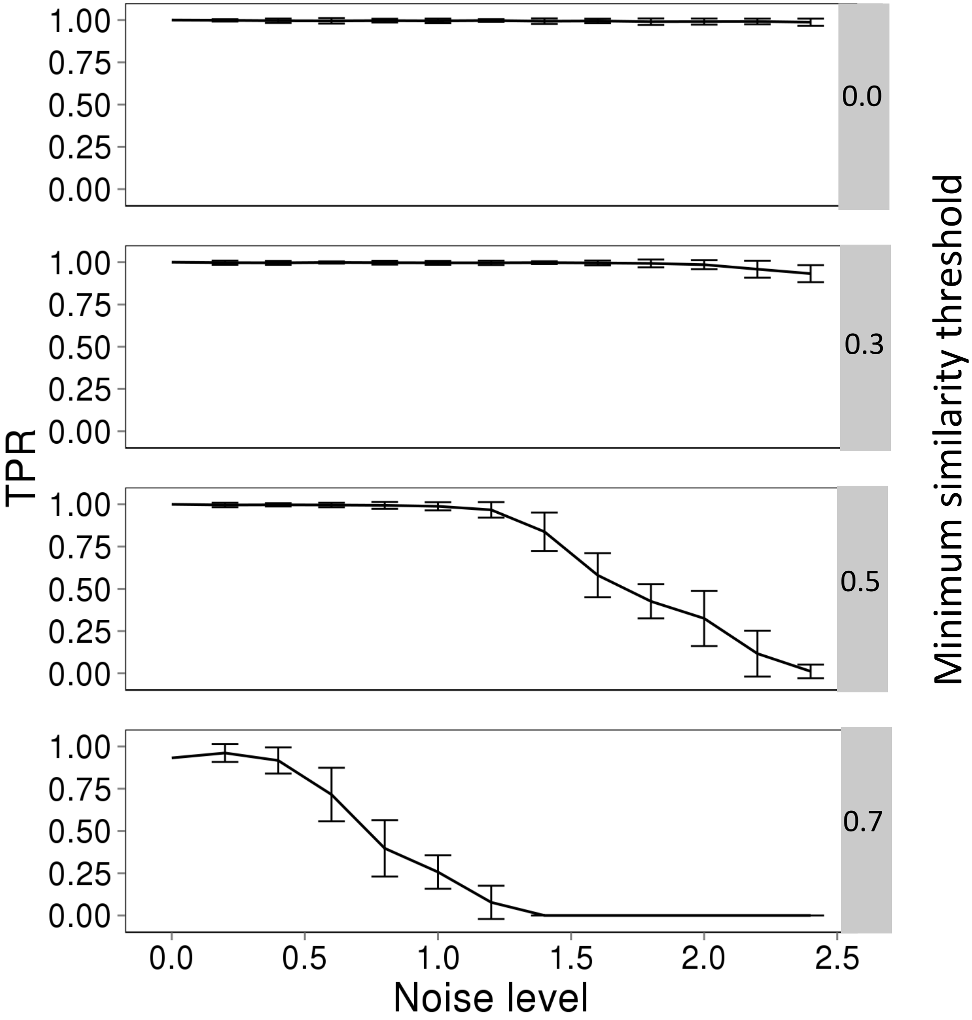

Supplementary Figure 6F
(unequal cluster size + unequal # clusters (1-4))

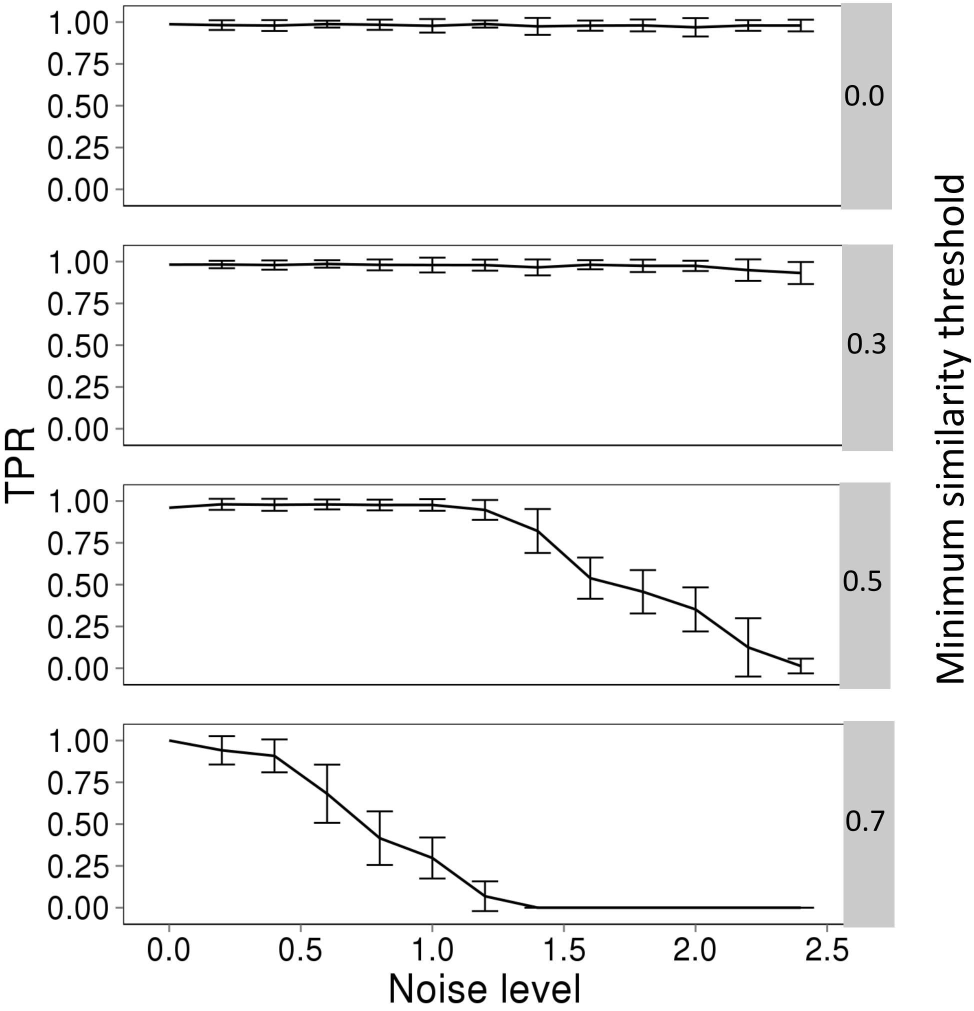

Supplementary Figure 6G
(50% high quality, 50% random noise clusters)

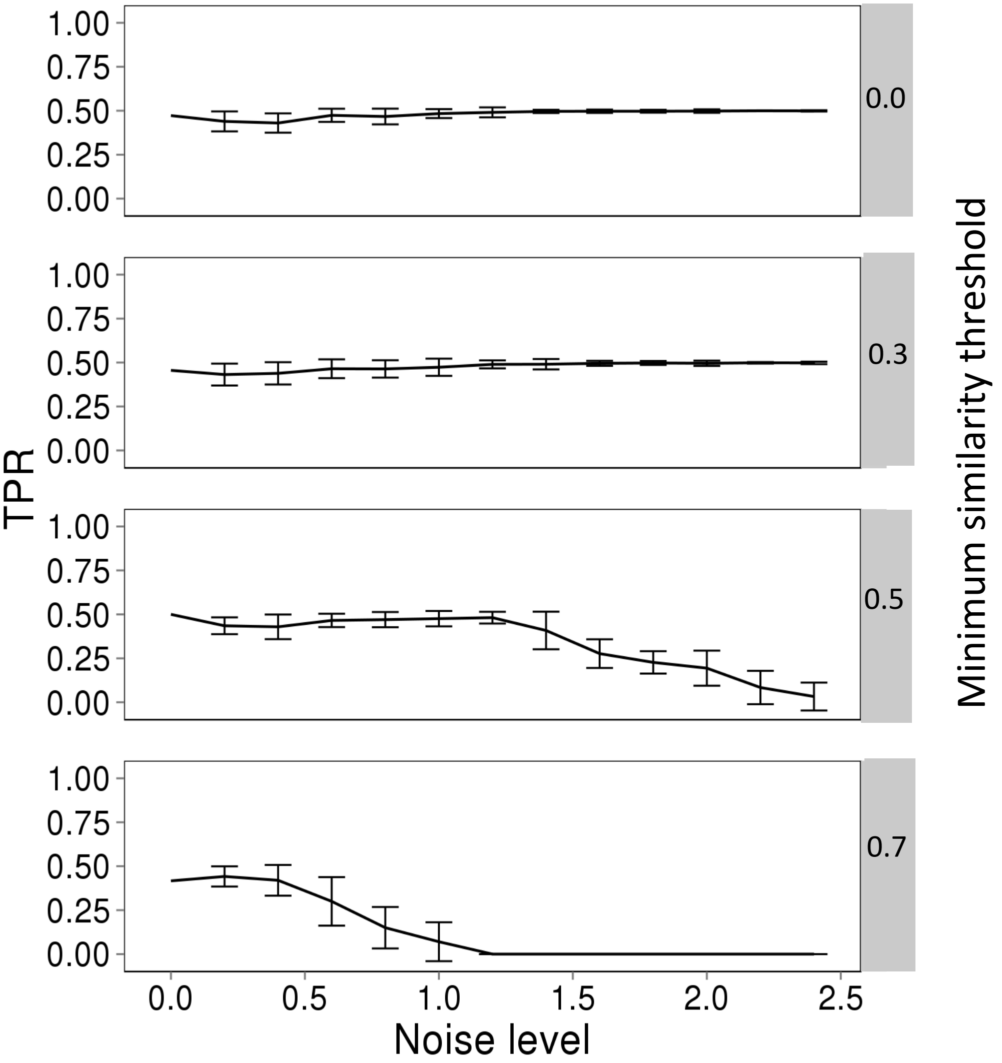

Supplementary Figure 7

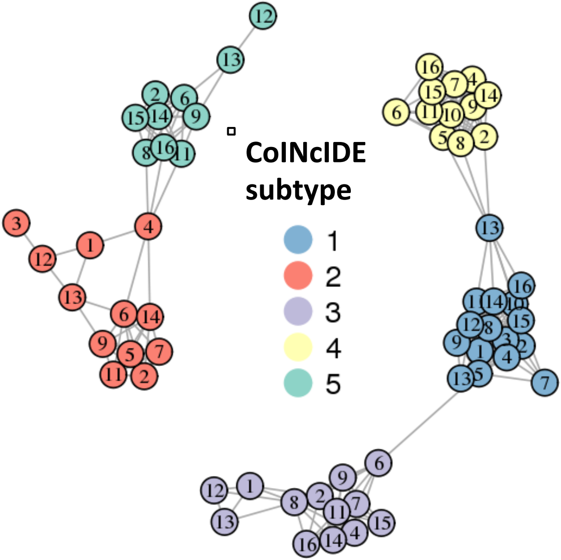

Supplementary Figure 8

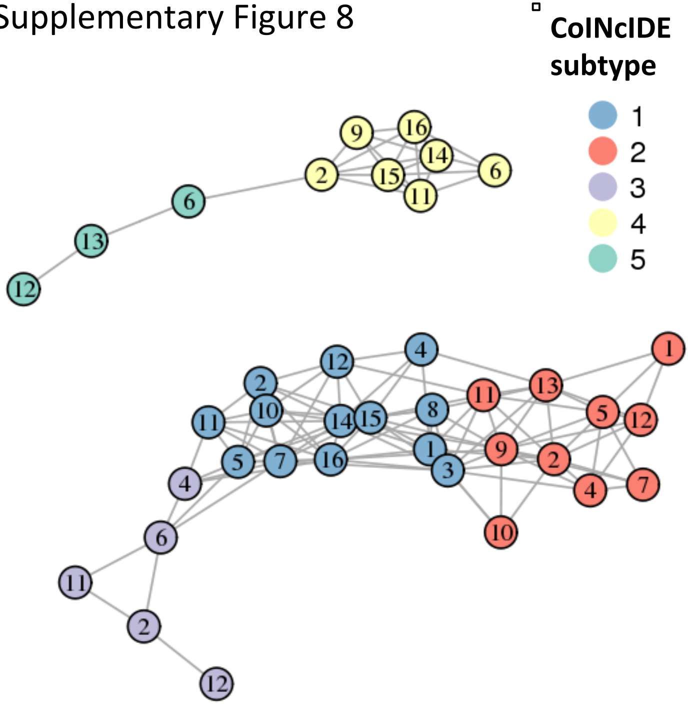

Supplementary Figure 9

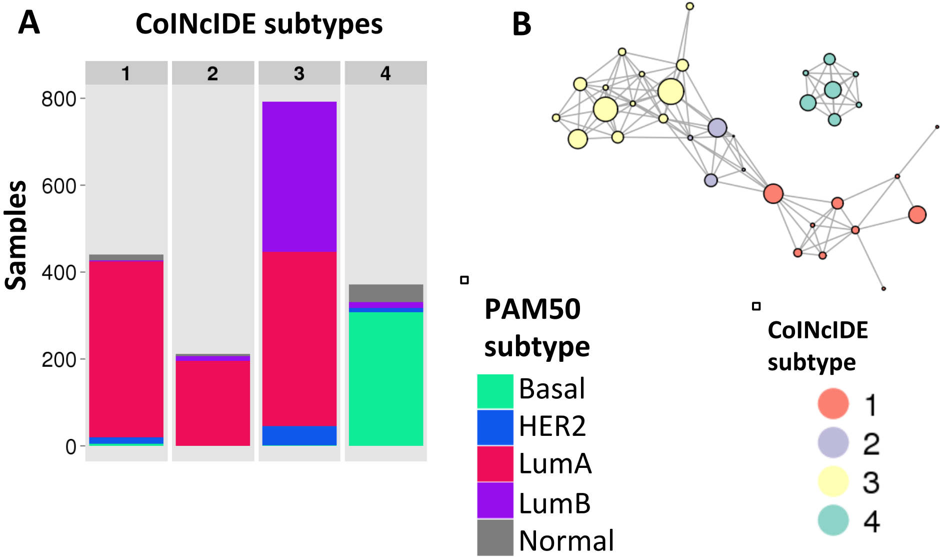

Supplementary Figure 10

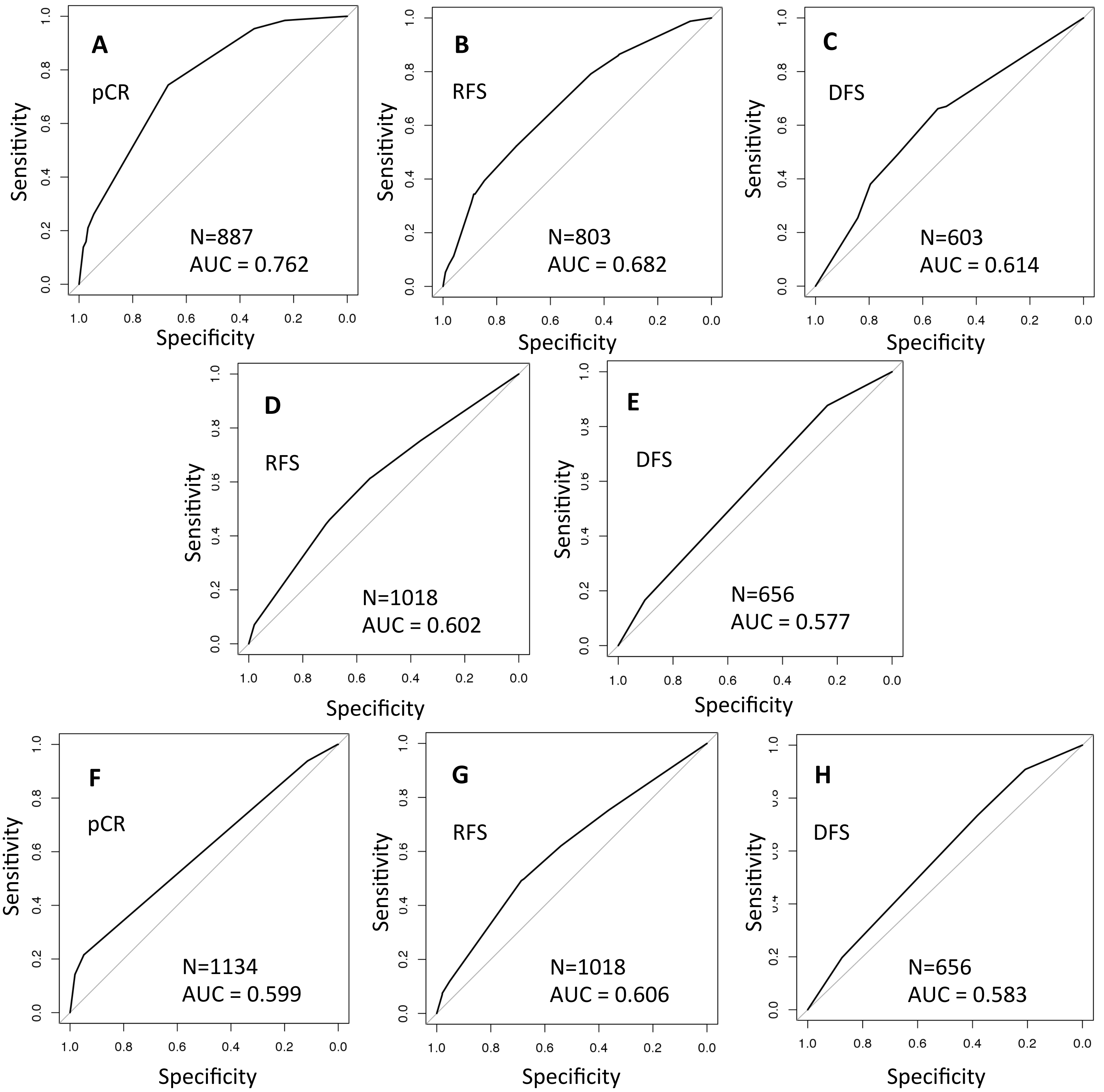

Supplementary Figure S10 continued

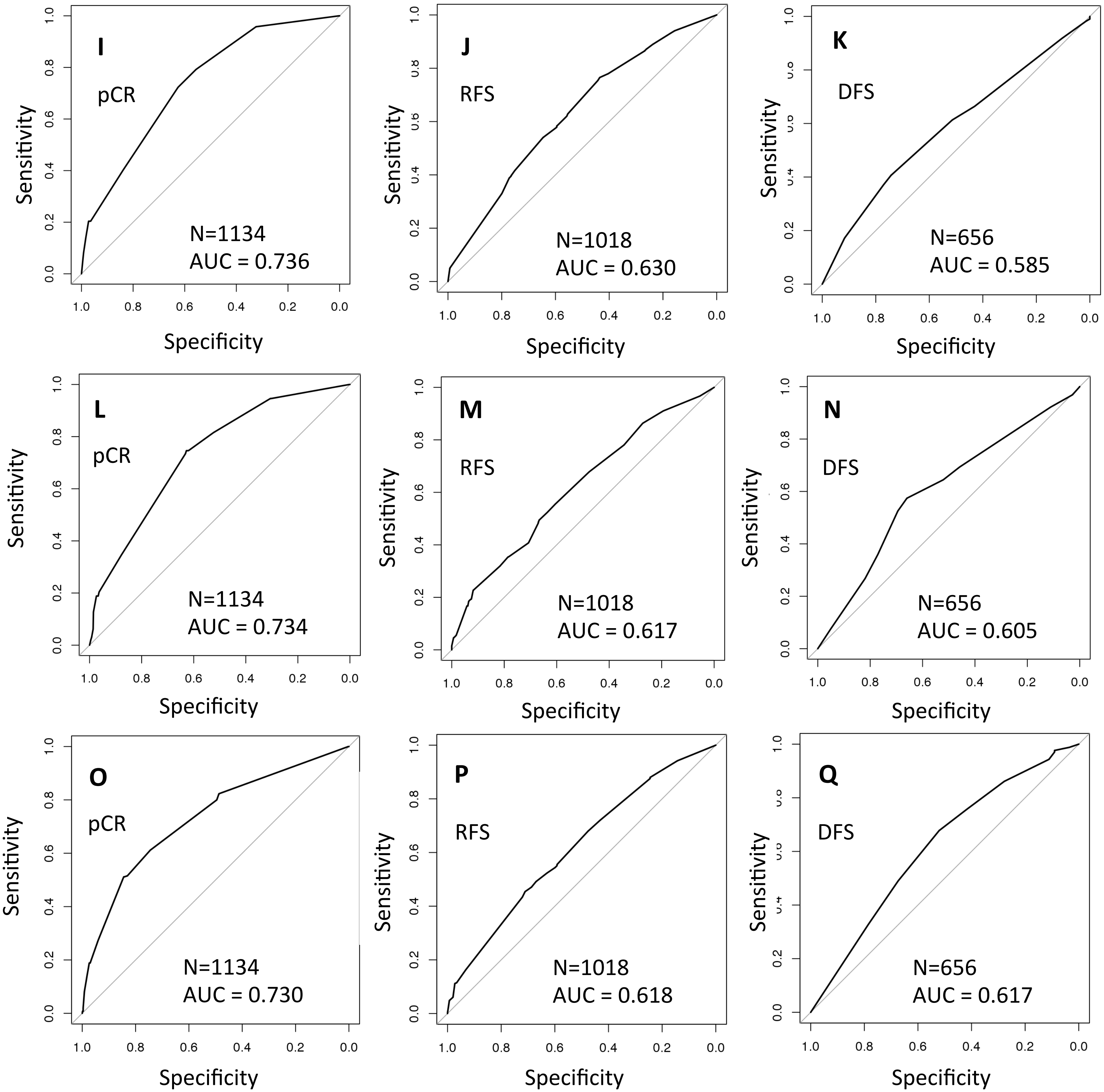

Supplementary Figure 10 continued

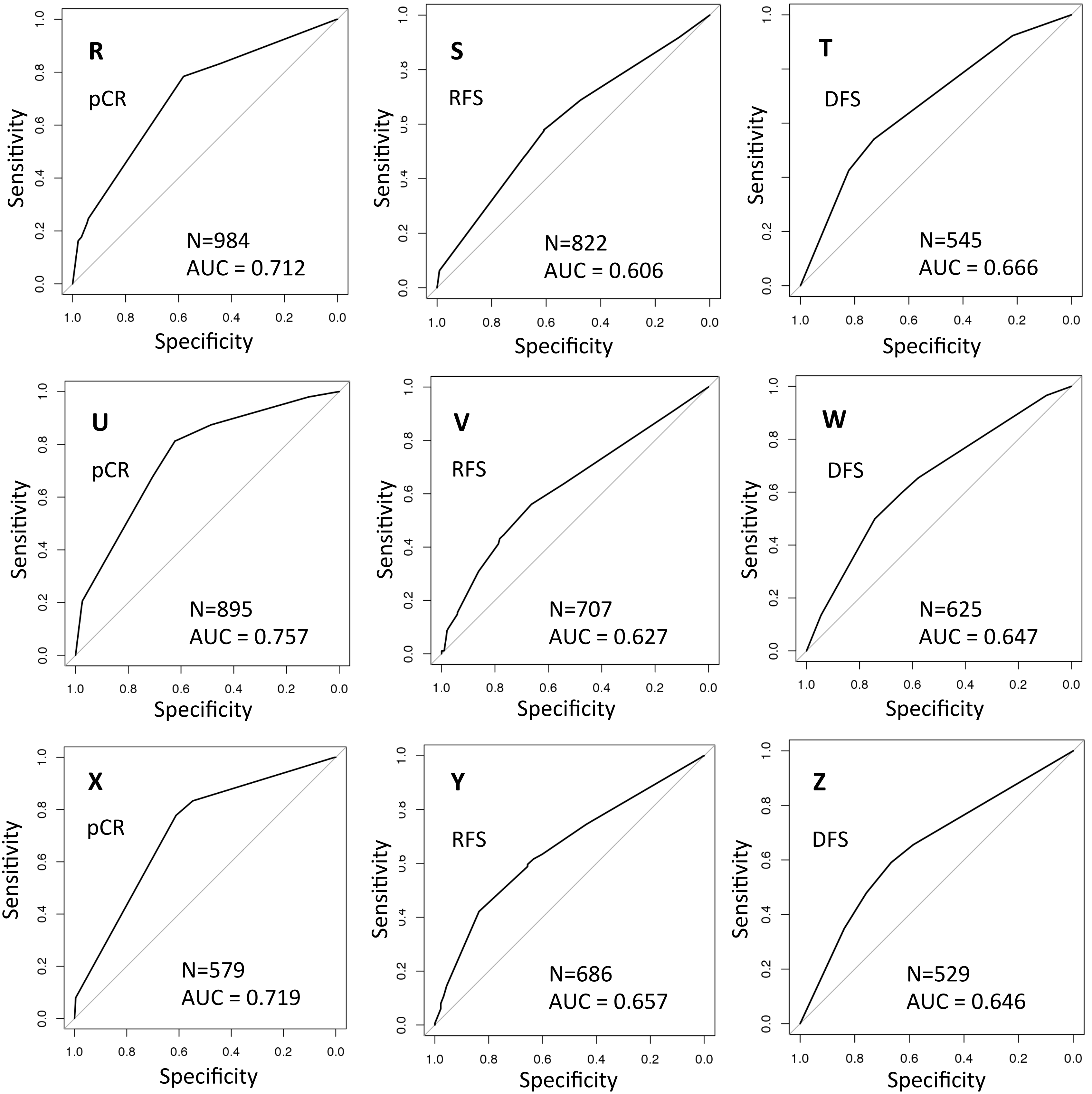

Supplementary Figure 11

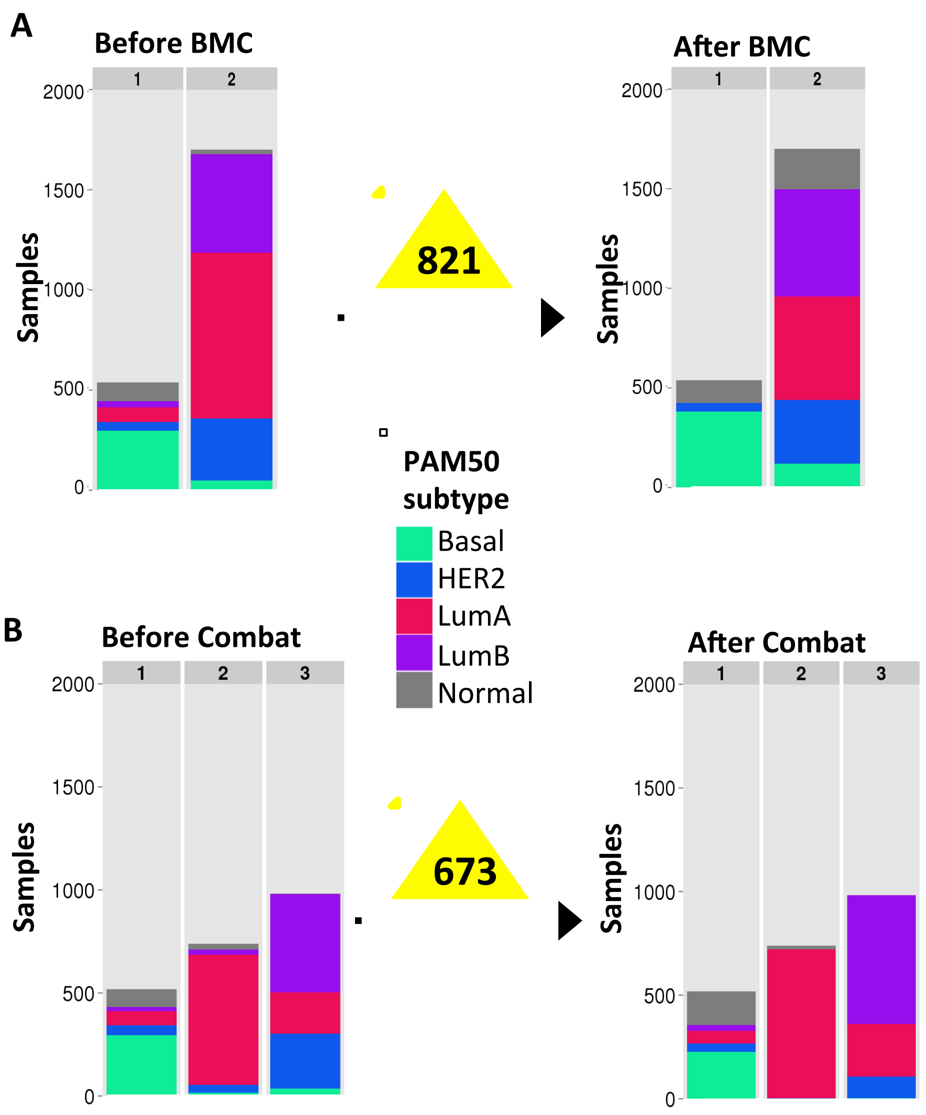

Supplementary Figure 12

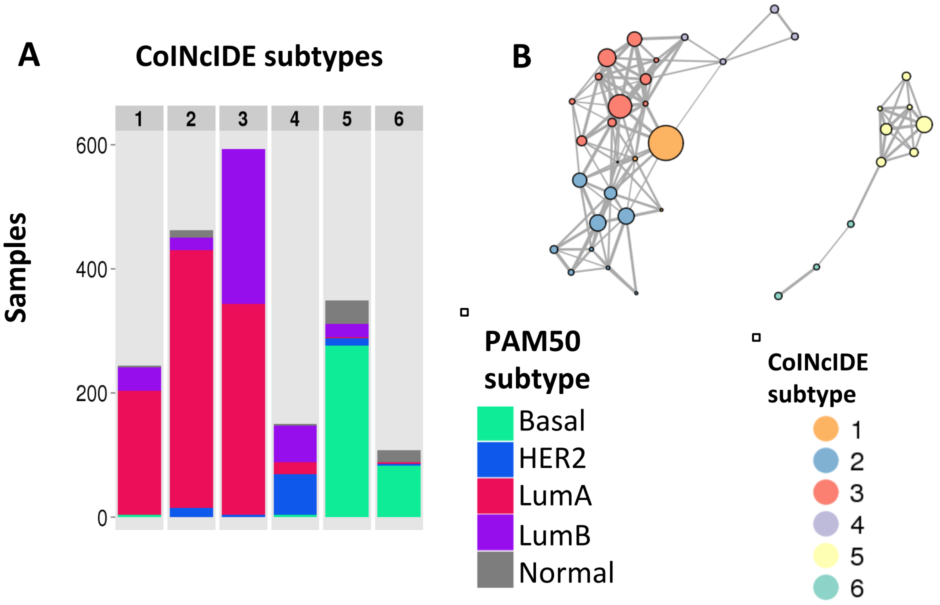

Supplementary Figure 13

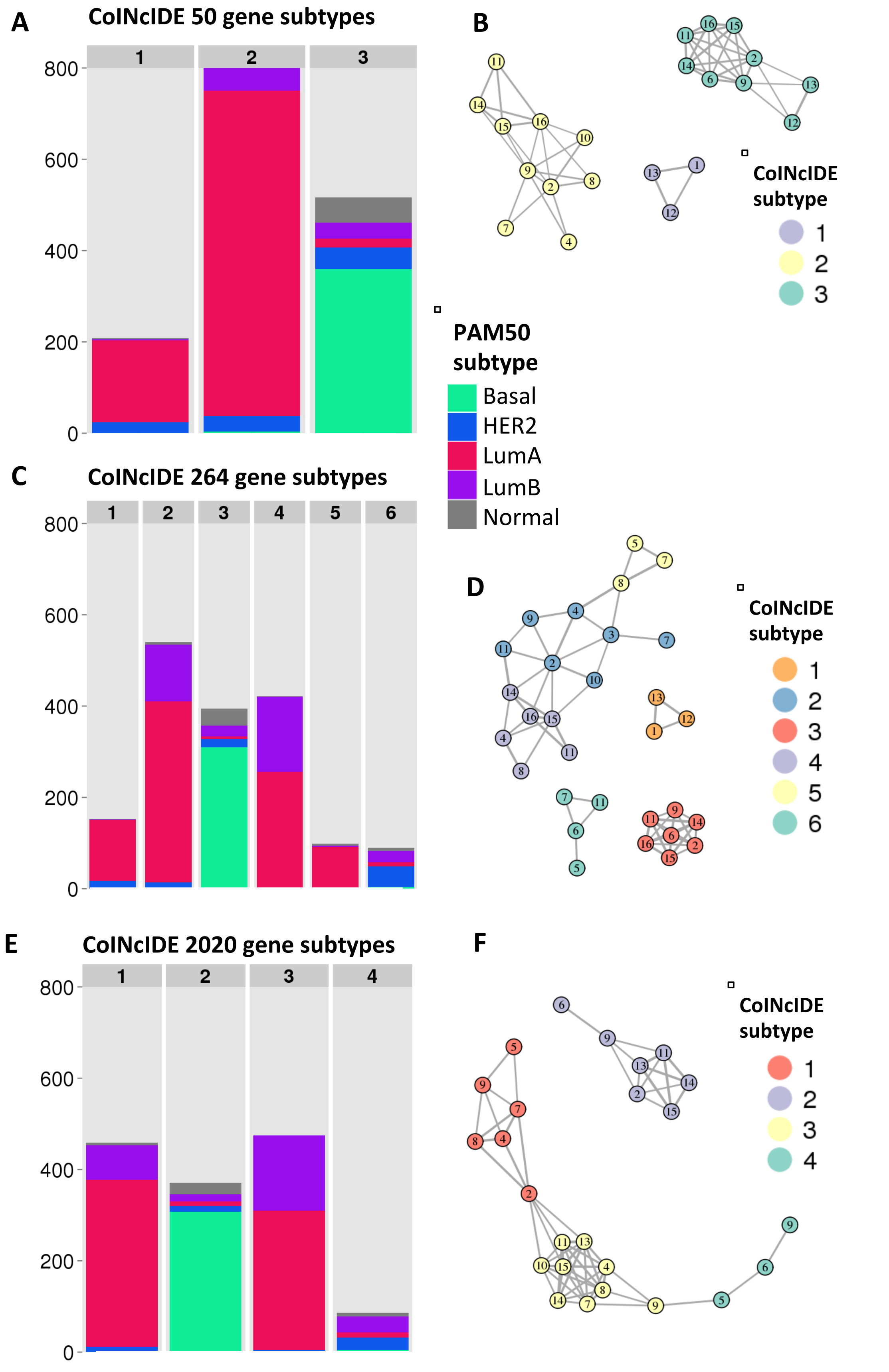

Supplementary Figure 14

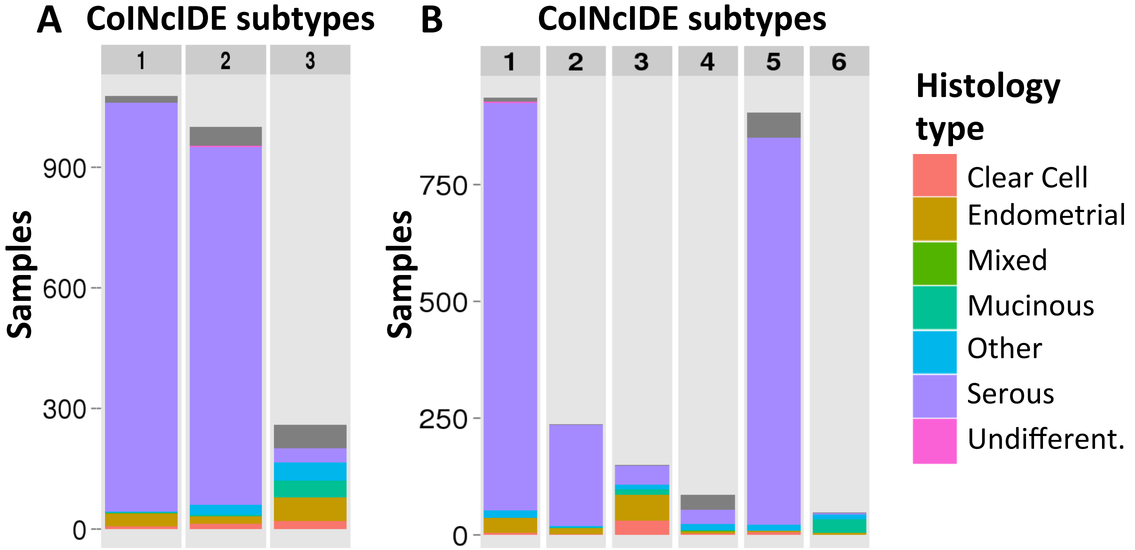

Supplementary Figure 15

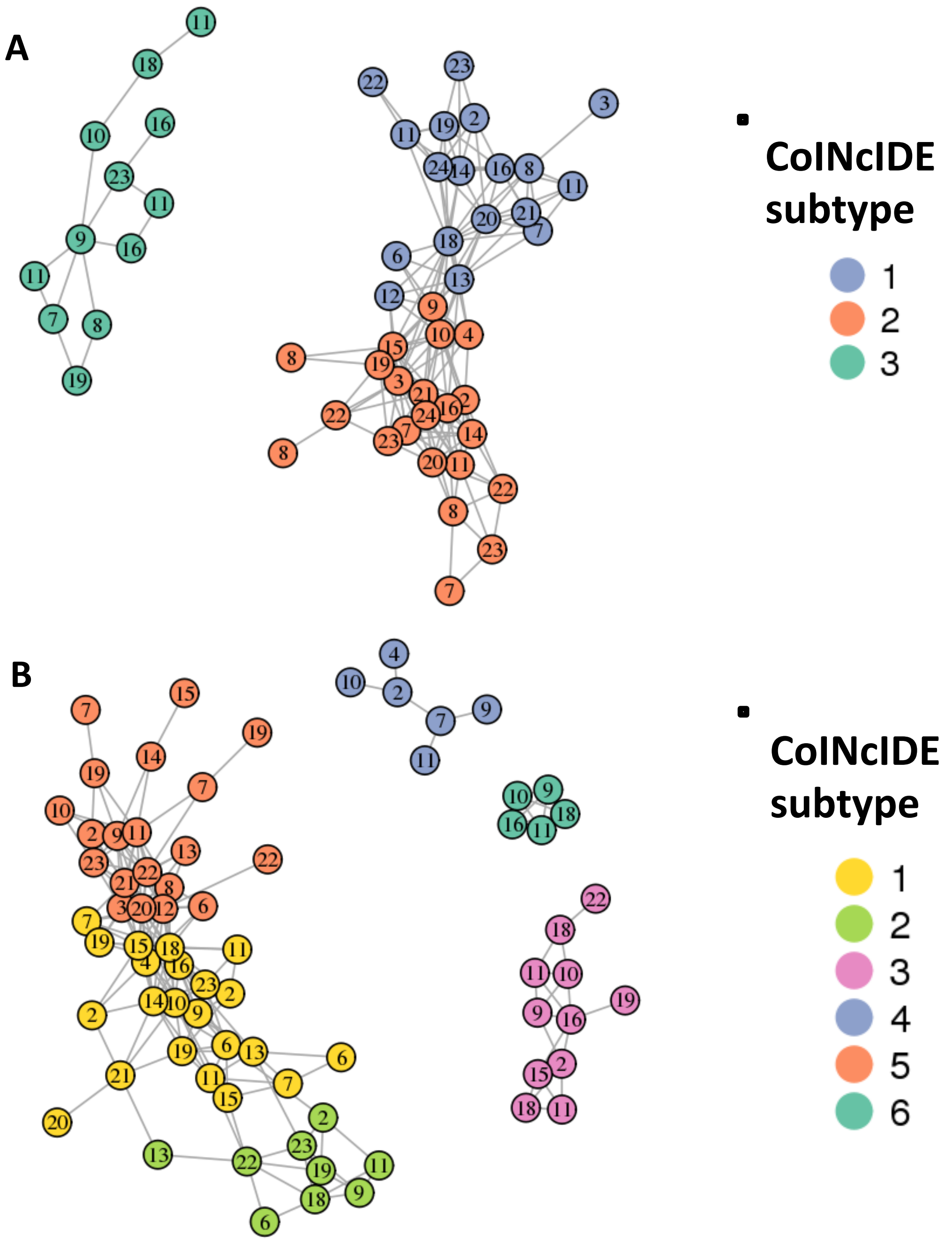

Supplementary Figure 16

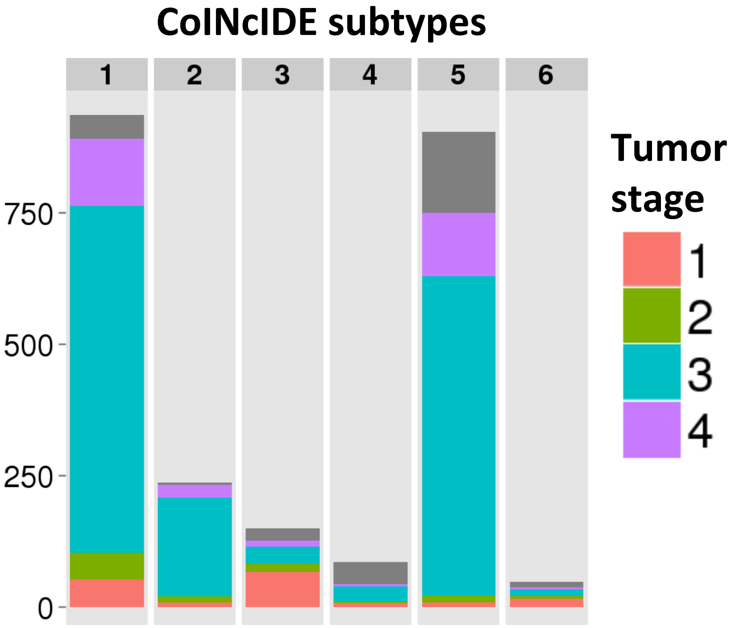

Supplementary Figure 17

A

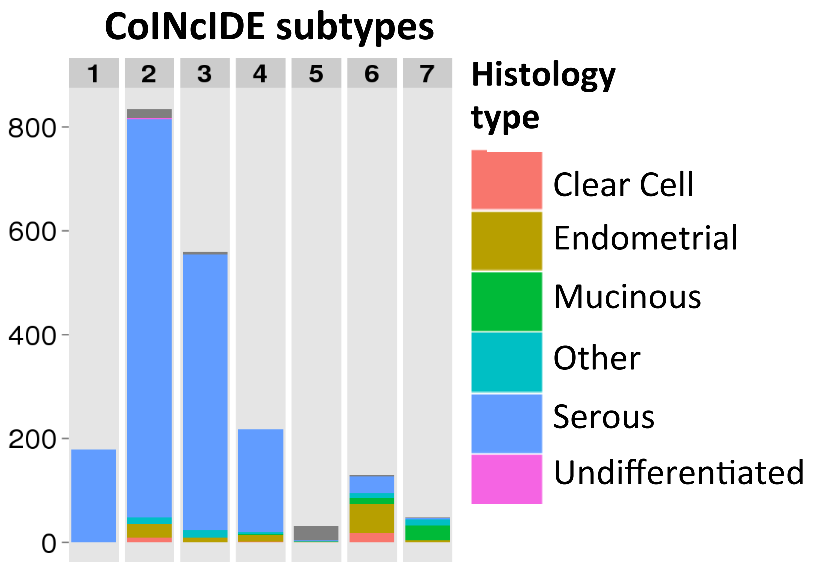

B

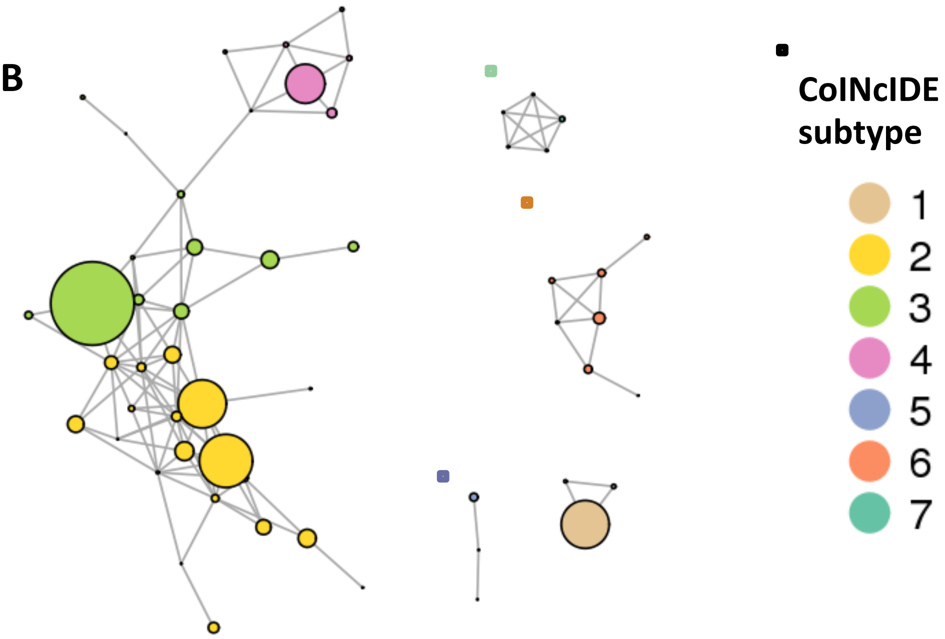

Supplementary Figure 18

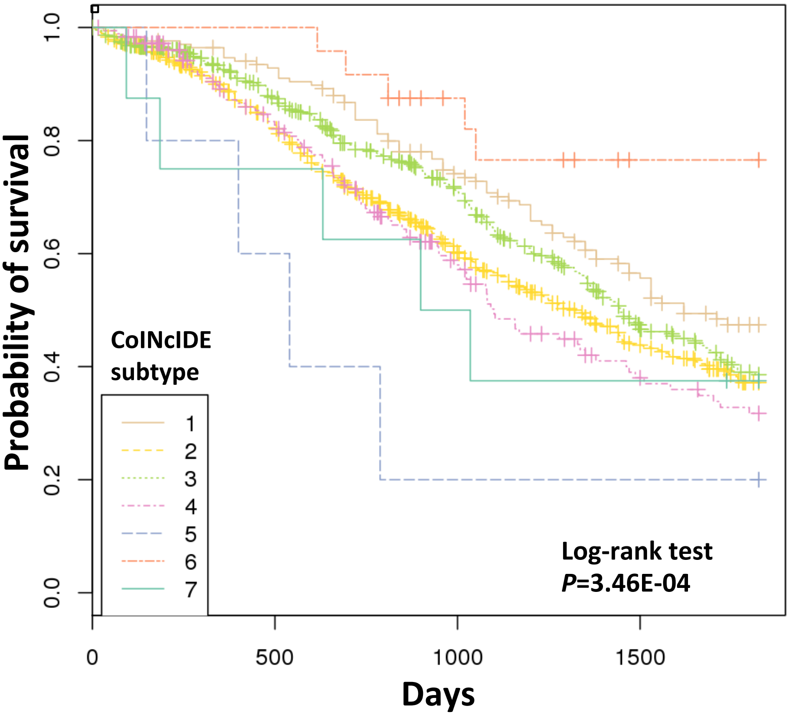

Supplementary Figure 19

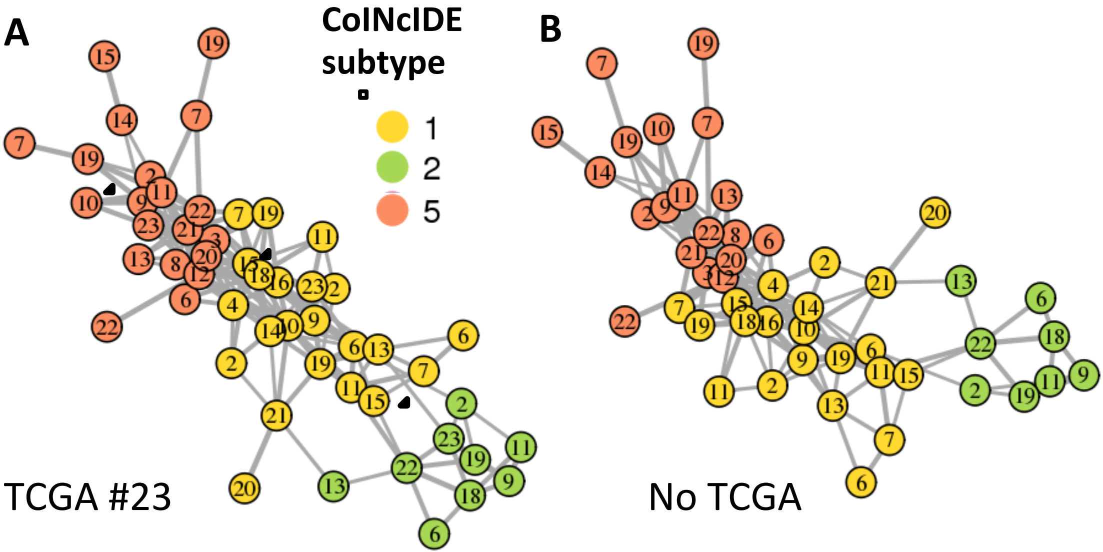

Supplementary Figure 20

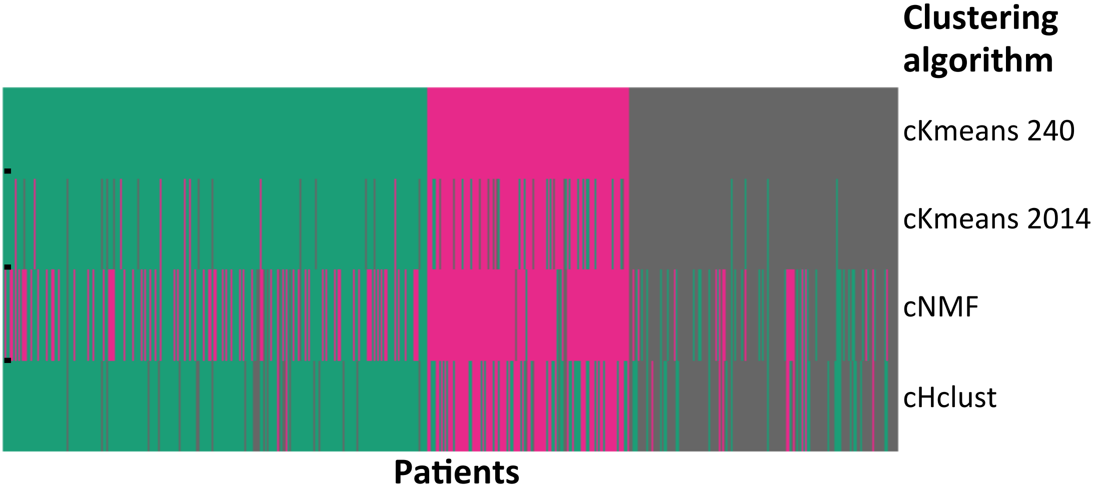

Supplement: Additional file 3: — All supplementary figures (labeled with the prefix ‘S’ in the main manuscript) and their corresponding legends. (PDF 7043 kb) [file 13073_2016_281_MOESM3_ESM.pdf]
